# Pharmaceutical screen identifies novel target processes for activation of autophagy with a broad translational potential

**DOI:** 10.1038/ncomms9620

**Published:** 2015-10-27

**Authors:** Santosh Chauhan, Zahra Ahmed, Steven B. Bradfute, John Arko-Mensah, Michael A. Mandell, Seong Won Choi, Tomonori Kimura, Fabien Blanchet, Anna Waller, Michal H. Mudd, Shanya Jiang, Larry Sklar, Graham S. Timmins, Nicole Maphis, Kiran Bhaskar, Vincent Piguet, Vojo Deretic

**Affiliations:** 1 Department of Molecular Genetics and Microbiology, School of Medicine, University of New Mexico Health Sciences Center, 915 Camino de Salud, NE, Albuquerque, New Mexico 87131, USA; 2 Cardiff Institute of Infection & Immunity, Cardiff University, School of Medicine, Henry Wellcome Building, Heath Park CF14 4XN, Cardiff, UK; 3 Department of Pathology, School of Medicine, University of New Mexico Health Sciences Center, 915 Camino de Salud, NE, Albuquerque, New Mexico 87131, USA; 4 College of Pharmacy, University of New Mexico Health Sciences Center, 915 Camino de Salud, NE, Albuquerque, New Mexico 87131, USA; 5 Department of Neurology, School of Medicine, University of New Mexico Health Sciences Center, 915 Camino de Salud, NE, Albuquerque, New Mexico 87131, USA; 6 Present address: Institute of Life Sciences, Bhubaneshwar, Odisa, India

## Abstract

Autophagy is a conserved homeostatic process active in all human cells and affecting a spectrum of diseases. Here we use a pharmaceutical screen to discover new mechanisms for activation of autophagy. We identify a subset of pharmaceuticals inducing autophagic flux with effects in diverse cellular systems modelling specific stages of several human diseases such as HIV transmission and hyperphosphorylated tau accumulation in Alzheimer’s disease. One drug, flubendazole, is a potent inducer of autophagy initiation and flux by affecting acetylated and dynamic microtubules in a reciprocal way. Disruption of dynamic microtubules by flubendazole results in mTOR deactivation and dissociation from lysosomes leading to TFEB (transcription factor EB) nuclear translocation and activation of autophagy. By inducing microtubule acetylation, flubendazole activates JNK1 leading to Bcl-2 phosphorylation, causing release of Beclin1 from Bcl-2-Beclin1 complexes for autophagy induction, thus uncovering a new approach to inducing autophagic flux that may be applicable in disease treatment.

## Abstract (web summary)

Autophagy is a homeostatic process that could be a potential drug target in the treatment of disease. Here the authors identify in a pharmaceutical screen flubendazole as an inducer of autophagy initiation and flux by affecting microtubules, mTOR, TFEB and Beclin 1 activity.

## Graphical abstract

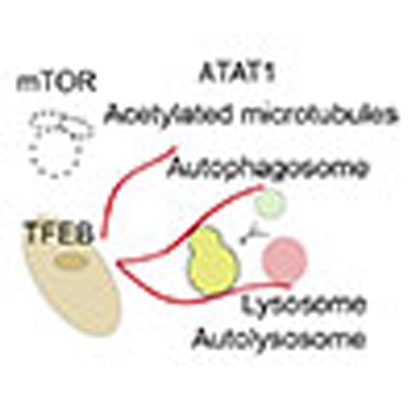

The autophagy pathway, defined by a set of autophagy factors^1^, plays a broad homeostatic role in cleansing the cellular interior by removing potentially toxic protein aggregates, defunct or surplus organelles^2^,^3^, and invading microbes as well as exogenous and endogenous agonists of inflammation^4^. Autophagy furthermore functions in cellular metabolism and cell death and survival^5^,^6^. Genetic and mechanistic studies have implicated autophagy in human health and disease, including cancer^7^, neurodegeneration^8^, aging-associated disorders and conditions^3^,^9^, and inflammatory and infectious diseases^4^.

Autophagy has a broad potential for pharmacological intervention in human diseases^3^,^10^, but no treatments have been implemented thus far using autophagy as a target process. Because of its significance in cancer^7^, autophagy has been employed in clinical trials^3^,^11^, through the use of rapamycin (autophagy activator through mTOR inhibition) and hydroxychloroquine, a generalized acidotropic neutralizer of the lysosomal organelles, and consequently also an inhibitor of autophagy. These trials have yielded mixed outcomes with additional results pointing to possible personalized or otherwise tailored applications^11^. Another approach has been to pursue design of membrane-permeant peptides that can activate^12^ or inhibit^13^ autophagy by targeting specific autophagy regulators, (for example, Beclin 1).

Several screens of small molecule libraries have been conducted for compounds that modulate autophagy^3^,^14^,^15^. These screens, in some cases monitoring only the changes in intracellular LC3 puncta as screen readouts, have yielded an overwhelmingly large number of drugs affecting autophagy^10^. An increase in LC3 puncta, while representing a convenient marker of autophagy^16^,^17^, can result either from induction of initiation or from a block of autophagic maturation since LC3 is consumed in autolysosomes^18^. Here, we discriminated against false positives by a second tier of autophagy maturation assays. Of many compounds that presented as hits in the initial imaging-based screen, only very few passed the more rigorous secondary assays for functional induction of the complete autophagy pathway including flux. The most consistent inducer in this smaller subset, flubendazole, showed effects in diverse biological output assays enabling cells to clear diverse disease-promoting agents through autophagy. Most importantly, by dissecting flubendazole mechanism of action in autophagy induction, we uncovered a novel dual-role of microtubules, which, when affected simultaneously by this drug, resulted in induction of the complete autophagy pathway.

## Results

### Identification of autophagy-modulating drugs

**Figure 1:**
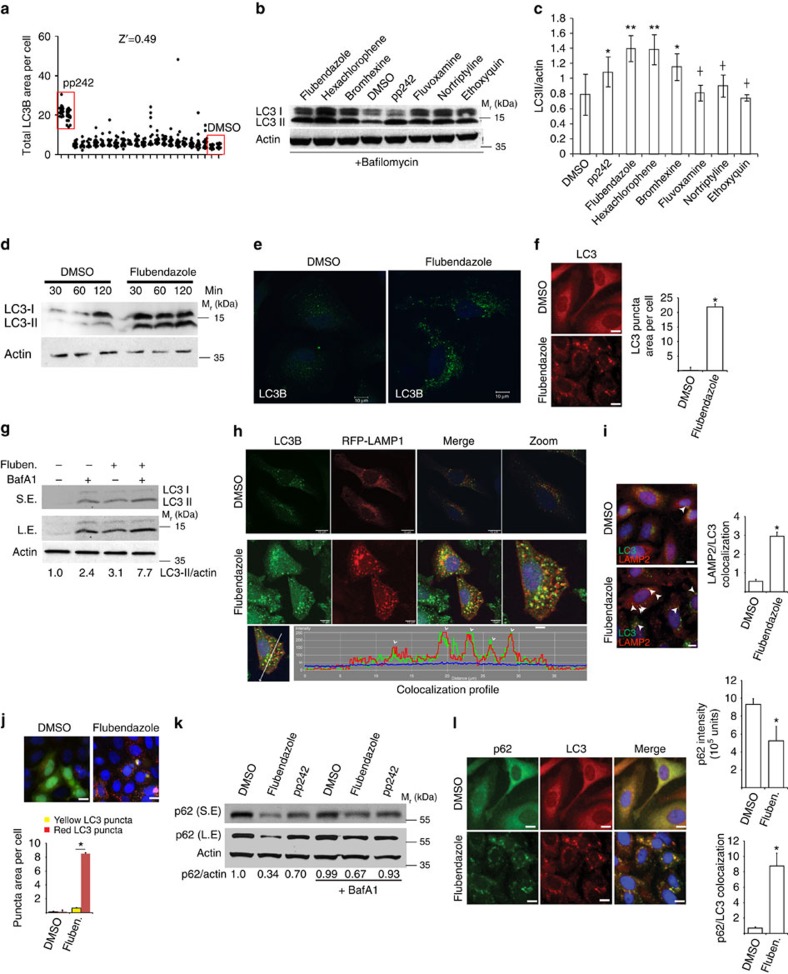
Screen of autophagy-modulating drugs. (**a**) Graph, example (384-plate) from screens of Prestwick, Spectrum and Johns Hopkins libraries (10 μM, 4 h) for autophagy-modulating drugs; high-content (HC) image analysis of LC3B puncta in HeLa cells stably expressing mRFP-GFP-LC3B. Data, total area of LC3B puncta per cell. DMSO, solvent control; pp242, positive control. (**b**) LC3B Immunoblot (HeLa lysates; Bafilomycin A1 and drugs used as indicated); see Supplementary Fig. 1. (**c**) Densitometric analysis; mean±s.e.; *P<0.05 (ANOVA), *n*=3. ANOVA, one way analysis of variance. (**d**) Immunoblot (DMSO or 5 μg ml^−1^ flubendazole-treated HeLa cells) with indicated antibodies. (**e**) Confocal images of HeLa (DMSO or 5 μg ml^−1^ Flubendazole, 1 h) with antibody to LC3B. (**f**) HC analysis of autophagosomes (LC3B puncta area) in HeLa cells; 1 h fludendazole or DMSO treatment. (**g**) Immunoblot analysis (LC3B) of HeLa treated with bafilomycin A1 and flubendazole (S.E, Short exposure; L.E, Long exposure). (**h**) Confocal images of RFP-LAMP1 (HeLa; treated with DMSO or 5 μg ml^−1^ flubendazole for 1 h); immunofluorescence, LC3B antibody. Bottom, colocalization tracing. (**i**) HC image analysis (total area of LC3 & LAMP2 colocalizing signals) following 1 h treatment of HeLa (flubendazole or DMSO). Arrows, colocalizing puncta. (**j**) HC analysis of autophagosomes (yellow dots) or autophagolysosomes (red dots) in HeLa stably expressing mRFP-GFP-LC3B (flubendazole or DMSO for 1 h). (**k**) Autophagy flux (p62 degradation); HeLa (2 h with compounds±bafilomycin A1). (**l**) HC analysis (p62 degradation); HeLa treated with DMSO or flubendazole for 2 h. Data, mean±s.e.; Statistics, Student’s *t-*test. *P< 0.05; **P< 0.01. fluben, flubendazole. Scale bars, 10 μm.

We used high-content imaging of LC3B puncta, a cell biological marker of autophagy^16^,^17^,^19^, combined with a stringent secondary assay of LC3B lipidation, also known as LC3-II conversion^19^. We carried out screens for autophagy-modulating drugs in HeLa cells stably expressing mRFP-GFP-LC3B (exemplified in Fig. 1a; summarized in Supplementary Fig. 1) and processed ranked data for presumptive hits shown in Supplementary Table 1. As a measure of autophagy modulation, two parameters were quantified based on green fluorescent protein (GFP) fluorescence in punctate profiles: the number of LC3B puncta per cell^16^ and the integrated total area of LC3B puncta per cell. The screen was carried out with dimethylsulphoxide (DMSO) solutions of compounds from three libraries: the Prestwick Chemical Library, the Microsource Spectrum 2000 library and the Johns Hopkins Library. Collectively, these libraries represent the majority of the FDA-approved drugs and those drugs that have been in human trials in Europe and Japan, apart from additional natural products and bioactive molecules. Only those compounds that in two independently executed complete library screens increased LC3 puncta by both parameters monitored, that is, LC3 puncta number and total area per cell (Supplementary Table 1), were considered further. These compounds were next tested for dose-dependent response (nM to μM range; Supplementary Fig. 2) and compared with the effects of pp242, a catalytic inhibitor of mTOR (Supplementary Fig. 2, last panel) in two independent experiments. Those drugs (a total of 80; Supplementary Fig. 1 and Supplementary Table 1) that elicited concentration-dependent autophagy response in both of the two dose-response series (Supplementary Fig. 2) were considered as autophagy modulators. Of these, 55 compounds with a wide range of structures (Supplementary Fig. 2) and known pharmacological activities (Supplementary Fig. 1 and Supplementary Table 1), have not been previously reported as autophagy regulators, whereas the balance of compounds (a total of 25) have been previously published, as indicated in Supplementary Table 1).

A subset of the compounds positive in LC3-puncta based screens and secondary assessments as above were subjected to analysis for effects on conversion of endogenous LC3 to its lipidated form LC3-II, as a measure of autophagy activation^17^,^19^. The cells were treated with compounds in the presence of bafilomycin A1 to block autophagic maturation, and compared with the effects of pp242, as exemplified in Fig. 1b,c. Treatment with bafilomycin A1 prevents autolysosomal degradation of LC3-II, thus permitting quantification of autophagy induction by measuring LC3-II/actin levels^17^,^19^. Only 30% of compounds passed this test relative to pp242, and reached a *P* value (analysis of variance) of <0.05 (Supplementary Table 2). The majority of these compounds increased LC3-II conversion in the presence of bafilomycin A1, consistent with being autophagy inducers. Nevertheless, three of the compounds (emetine, ethoxyquin and GBR12909) showed negative effects on the LC3-II levels in the LC3-II conversion assay in the presence of bafilomycin A1, and thus acted as inhibitors of autophagy induction. However, since they caused increase in LC3B puncta they also blocked autophagic maturation, and should be considered as novel inhibitors of the whole autophagic pathway. Significantly, among the best and most consistent inducers of autophagy in all assays was flubendazole (Fig. 1, Supplementary Fig. 2, and Supplementary Tables 1 and 2) an anti-helminthic drug and a novel inducer of autophagy identified here.

### Flubendazole activates autophagy and autophagic flux

Flubendazole increased total cellular LC3 levels as determined by immunoblotting (Fig. 1d), immunofluorescence (Fig. 1e) and high-content microscopy (Fig. 1f). This was not due to a block in the autophagic flux because flubendazole caused increased LC3-II conversion in the presence of bafilomycin A1 (Fig. 1b,c,g). Furthermore, flubendazole-induced LC3B^+^ autophagic organelles were positive for the lysosomal markers LAMP1 and LAMP2 (Fig. 1h,i). The colocalization between flubendazole-induced autophagic organelles and LAMP1 (Fig. 1h) and a separately carried out high-content analysis of colocalization with LAMP2 (Fig. 1i) indicated that flubendazole-induced autophagic flux. This was further confirmed using the tandem mRFP-GFP-LC3B probe, which showed an increase both in yellow puncta (RFP^+^ GFP^+^; representing early autophagic organelles) and red puncta (RFP^+^ GFP^-^, representing autolysosomes) after flubendazole treatment (Fig. 1j)^18^. Moreover, when p62/sequestosome 1 (p62), a marker for autophagic degradation^20^, was monitored (Fig. 1k,l), it was degraded on flubendazole treatment. This effect was abrogated in the presence of the autophagic maturation inhibitor, bafilomycin A1, as determined by immunoblotting (Fig. 1k). LC3-p62 colocalization increased in flubendazole-treated cells and the total p62 cellular content was reduced (Fig. 1l). Thus, flubendazole is a newly identified potent inducer of autophagy and autophagic flux.

### Flubendazole affects microtubules and displaces mTOR

**Figure 2:**
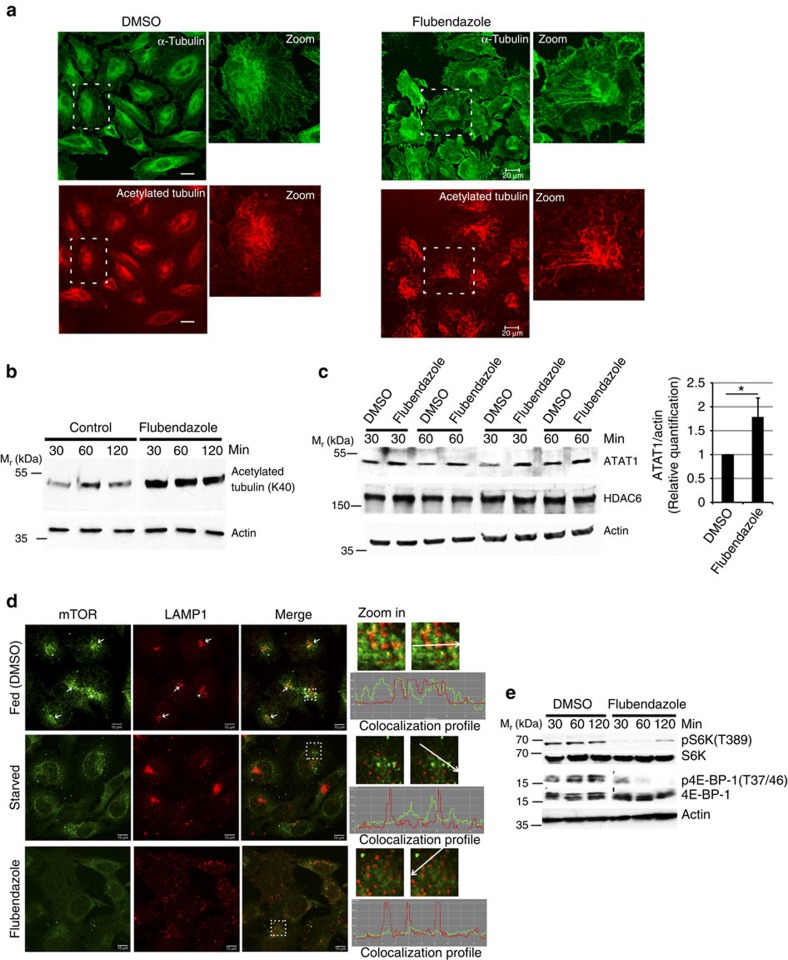
Flubendazole reciprocally controls dynamic and acetylated microtubules and affects mTOR. (**a**) Confocal images of HeLa cells treated with DMSO or flubendazole (1 h, 5 μg ml^−1^); immunofluorescence with antibody to acetylated tubulin and α-tubulin. Antibodies against α-tubulin (Abcam, ab6161; green fluorescence) and acetylated tubulin Sigma (clone 6-11B-1; red fluorescence) were imaged with constant (per fluorophore) excitation, emission, pinhole and exposure time. Scale bars, 20 μm. (**b**) Immunoblot analysis of acetylated tubulin (antibody: Sigma clone 6-11B-1) levels in lysates from HeLa cells treated with ethanol or 5 μg ml^−1^ flubendazole in ethanol (see Supplementary Fig. 3b for DMSO as a solvent). (**c**) Immunoblot analysis of ATAT1 and HDAC6 levels in lysates from HeLa cells treated with DMSO or 5 μg ml^−1^ flubendazole (in DMSO) for different durations as indicated. Graph, quantification from three different experiments; mean±s.e.; Student’s *t-*test. *P<0.05. (**d**) Confocal images of HeLa cells starved (1 h) or treated with DMSO or flubendazole (1 h, 5 μg ml^−1^) and immunostained with antibodies to mTORC1 and LAMP1. Dashed squares, fields with tracings for colocalization analysis. Fed, full medium control where mTOR localizes on lysosomes, versus starvation or flubendazole treatment. (**e**) HeLa cells treated with DMSO or flubendazole (5 μg ml^−1^) were lysed and subjected to immunoblotting with indicated. Scale bars, 20 μm (**a**) and 10 μm (**d**).

Next, we examined the mode of action of flubendazole. We observed that flubendazole can disrupt the regular microtubules (Fig. 2a, Supplementary Fig. 3a). This was in keeping with flubendazole being a member of the benzimidazole family of drugs that affect tubulin polymerization^21^. However, in variance with the other common microtubule depolymerizing agents (nocodazole, vinblastine and vincristine; Supplementary Table 3), flubendazole strongly increased acetylated lysine-40 (K-40) tubulins (Fig. 2a,b, Supplementary Fig. 3a,b), a modification known to maintain stability of microtubules even under depolymerization conditions^22^,^23^. Note that acetylated tubulin in flubendazole-treated cells stained only partially with α-tubulin antibody, most likely due to the competition for the antibody of depolymerized α-tubulin released from dynamic microtubules in the cytosol (Fig. 2a). Two enzymes, ATAT1 (αTAT1/MEC-17) acetyltransferase and HDAC6 deacetylase, among others, regulate acetylation levels of α-tubulin^24^,^25^. ATAT1 is a major α-tubulin acetyltransferase whereas HDAC6 is a main microtubule deacetylase. We tested the effect of flubendazole on ATAT1 and HDAC6 levels and found that flubendazole increased the total amount of ATAT1 without affecting HDAC6 levels (Fig. 2c). Increased ATAT1 enzyme could be an underlying reason for enhanced acetylated tubulins in flubendazole-treated cells. Indeed, when we knocked down ATAT1, the acetylated tubulins in flubendazole-treated cells were strongly reduced (Supplementary Fig. 3c). Thus, flubendazole has a dual action—it depolymerizes dynamic microtubules while stabilizing acetylated microtubules.

The Ser/Thr protein kinase mTOR is localized on the lysosomal organelles and its activity is dependent on both nutritional status and lysosomal function^26^,^27^. Starvation, a classical inducer of autophagy, displaces mTOR from lysosome linked to its inactivation^26^,^27^. Treatment of HeLa cells with flubendazole displaced mTOR from lysosomes (Fig. 2d) and inactivated mTOR as indicated by the inhibition of phosphorylation of mTOR downstream effectors, S6 kinase and 4EBP-1 (Fig. 2e). This effect of flubendazole on mTOR signalling could be explained as a result of disruption of dynamic microtubules. Microtubules are important for lysosome movement and their disruption can cause lysosomal stress or abnormal lysosomal function^28^, which can lead to mTOR displacement from lysosome membrane and its inactivation as observed here (Fig. 2d,e). Inactivation of mTOR signalling on flubendazole treatment of the cells was accompanied by attenuated Akt phosphorylation (Supplementary Fig. 4a), which upstream of mTOR contributes to mTOR activation. Localization of Akt on microtubules maintains its activity, while disruption of microtubules reduce activating Akt phosphorylation^29^. Thus, the disruption of microtubules by flubendazole plays an important role in inactivating mTOR signalling therefore leading to autophagy induction.

### Flubendazole activates TFEB and lysosome biogenesis

**Figure 3:**
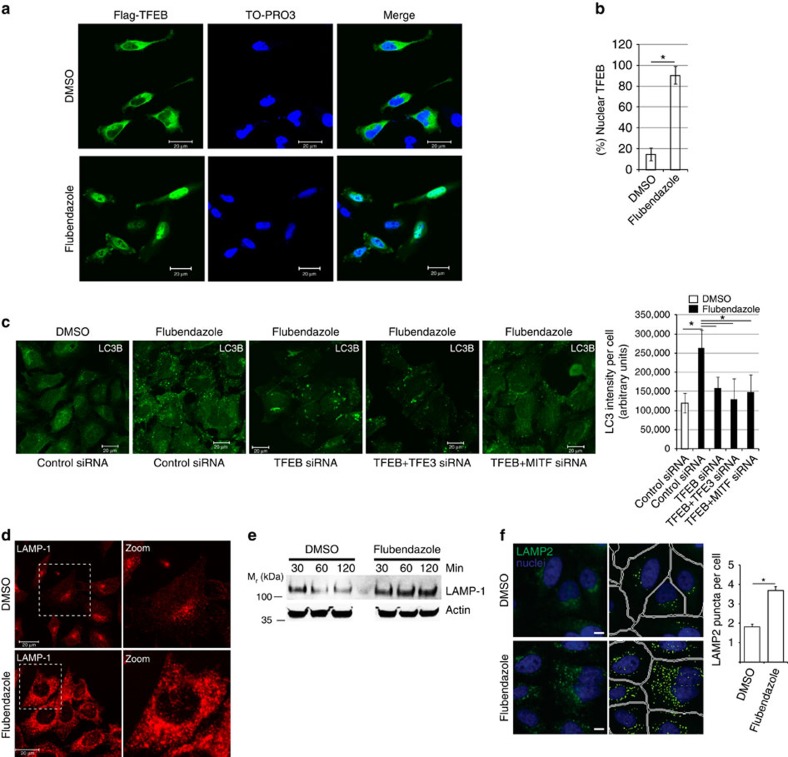
Flubendazole activates TFEB nuclear translocation to induce autophagy. (**a**) Confocal images of FLAG-TFEB transfected HeLa cells treated with DMSO or flubendazole (2 h, 5 μg ml^−1^) and subjected to nuclear stain (TO-PRO3) and Flag immunostaining (Flag-TFEB). Scale bars, 20 μm. (**b**) Graph shows percentage of cells with nuclear TFEB±s.d. (*n*=3; >100 cells from 20 fields total). *P<0.05 (Student’s *t*-test). (**c**) Confocal images of HeLa cells knocked down for TFEB and other TFE/MiTF family members as indicated were treated with DMSO or flubendazole (1 h, 5 μg ml^−1^) and immunostained for LC3B. Graph, LC3 intensity per cell±s.d. (∼30 cells per condition was analysed using ImageJ, *n*=3); ANOVA (one way analysis of variance) was used to test for statistical significance: *P<0.05. Scale bars, 20 μm. (**d**) Confocal images of HeLa cells treated with DMSO or flubendazole (1 h, 5 μg ml^−1^) and immunostained for LAMP1. (**e**) Immunoblotting analysis of LAMP1 content in DMSO or 5 μg ml^−1^ fubendazole-treated HeLa cells. (**f**) High-content analysis of the abundance of lysosomes in HeLa cells treated with flubendazole or DMSO for 1 h. Statistics, mean±s.e.; Student’s *t-*test. *P< 0.05 (*n*>3). Scale bars, 10 μm.

Activity of TFEB, a key positive regulator of autophagy and lysosome biogenesis, is regulated by the status of mTOR on lysosomes^30^,^31^,^32^,^33^. Under fed conditions, on lysosomal membranes, mTOR phosphorylates TFEB and inhibits its activity, whereas under starvation conditions or lysosomal stress, inhibition of mTOR leads to TFEB activation and its nuclear translocation^31^,^32^. Since flubendazole inactivated mTOR signalling, we next tested whether flubendazole influenced TFEB by monitoring its nuclear translocation and found that the percentage of nuclei showing TFEB positivity was greatly increased in flubendazole-treated cells (Fig. 3a,b). Flubendazole increased LC3 levels (both LC3-I and LC3-II; Supplementary Fig. 4b–d). The increase in LC3-I and LC3-II levels was reduced by knocking down TFEB. Thus, TFEB is important for flubendazole-induced increase in LC3 levels, in keeping with it being a regulator of *MAP1LC3B* expression^33^.

Flubendazole increased LC3-II/actin (Fig. 1b–e and Supplementary Fig. 4c) and LC3B puncta/cell (Fig. 1f,g and Fig. 3c), which are classical measures of autophagy^17^. As in the case of LC3-II/actin levels (Supplementary Fig. 4c), the increase in LC3 puncta in flubendazole-treated cells also depended on TFEB (Fig. 3c). A double knockdown of TFEB with TFE3 or MITF (additional members of the MiT/TFE family of regulators^34^) in the same cells did not further reduce LC3 puncta elicited by flubendazole (Fig. 3c), although the altered morphology of LC3 profiles associated with TFEB knockdown (consistent with a recent report^34^) was corrected by a concomitant knockdown of MITF but not TFE3 (Fig. 3c, compare three rightmost panels). Thus, TFEB is important for flubendazole-induced autophagy.

Next, we analysed the effect of flubendazole on lysosome biogenesis by monitoring the amount of LAMP1, a marker of lysosomes. The results showed an increase in total amounts of LAMP1 by immunofluorescence and immunoblotting following flubendazole treatment (Fig. 3d,e). LAMP2 was also increased in flubendazole-treated cells, assessed by high-content microscopy and analysis (Fig. 3f). These data show that flubendazole treatment increases lysosome abundance in cells. Consistent with the flubendazole-mediated activation of TFEB, the transcription of TFEB target genes, *LAMP1* and *HEXA* was modestly increased at 1 h following flubendazole treatment (Supplementary Fig. 4e). This effect was abrogated by a TFEB alone (or in combination with MITF) knockdowns (Supplementary Fig. 4f). However, TFEB (alone or in combination with other MiT/TEF factors) knockdowns did not reduce protein levels of LAMP1 induced by 1 h of flubendazole treatment (Supplementary Fig. 4b,g). This suggests additional levels of expression control besides transcription at which flubendazole acted on LAMP1. The existence of additional levels of control were exemplified by reduced levels of flubendazole-induced LAMP1 by brefeldin A (Supplementary Fig. 5a); brefeldin A prevents activation of ARFs within the biosynthetic pathway affecting transport of lysosomal proteins through the secretory pathway, which normally might be limited by the retrograde transport along microtubules from the Golgi to the endoplasmic reticulum (ER)^35^. However, additional mechanisms outside of *de novo* mRNA synthesis are likely involved.

Taken together, these data indicate that flubendazole induces autophagic initiation by inactivating mTOR but also promotes lysosomal and autolysosomal maturation.

### Flubendazole releases Bcl-2 from Beclin 1

**Figure 4:**
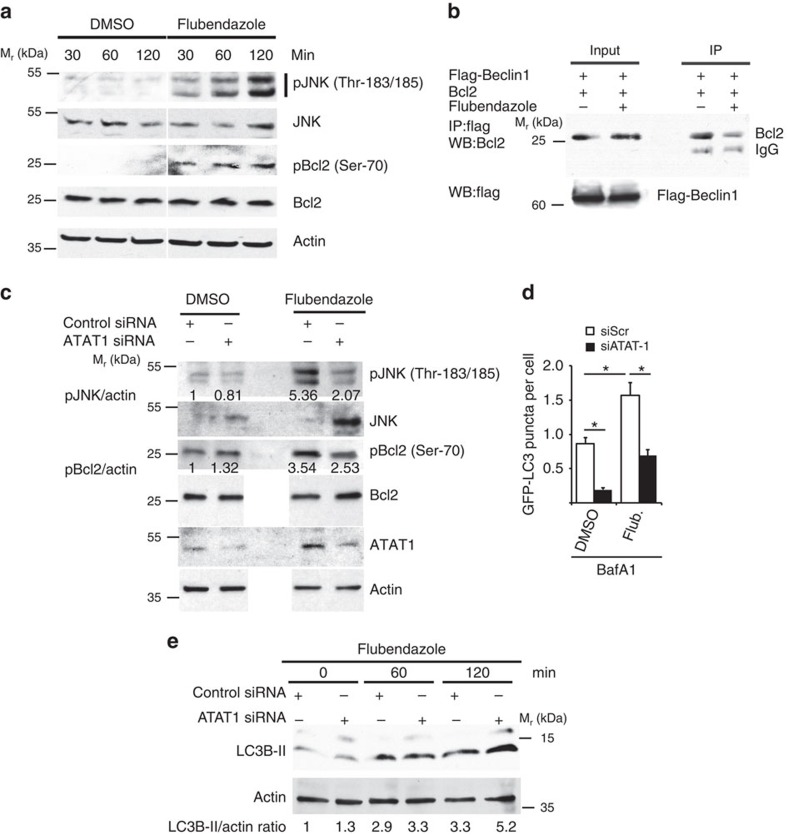
Flubendazole activates JNK1 and Bcl-2 and relieves Beclin 1 from negative regulation. (**a**) HeLa cells treated with DMSO or flubendazole (5 μg ml^−1^) were lysed and subjected to immunoblotting. (**b**) Coimmunoprecipitation analysis of interaction between Beclin 1 and Bcl-2 (HEK293T lysates) in cells treated with DMSO or 5 μg ml^−1^ flubendazole for 2 h. (**c**) HeLa cells knocked down for ATAT1 and treated with DMSO or 5 μg ml^−1^ flubendazole (for 2 h) were lysed and subjected to immunoblotting. (**d**) High-content analysis of the abundance of LC3 puncta in HeLa cells knockdown for ATAT1 or control cells treated with flubendazole or DMSO for 45 min. Flub, flubendazole; statistics, mean±s.e.; Student’s *t-*test. *P<0.05 (*n*=3). (**e**) HeLa cells subjected to control (scrambled siRNA) or ATAT1 siRNA were induced with flubendazole for the indicated times, in the absence of other autophagic flux inhibitors, and LC3-II levels were determined by immunoblotting.

Starvation-induced acetylation (K-40) of microtubules increases the recruitment of molecular motors kinesin-1 and JIP-1 (JNK-interacting protein 1) to microtubules leading to c-Jun N-terminal kinase-1 (JNK1) phosphorylation and activation^36^,^37^. Activation of JNK1 has been shown to lead to Bcl-2 phosphorylation causing release of Beclin 1 from Bcl-2-Beclin 1 complexes, resulting in autophagy induction^38^. Since flubendazole increased acetylation of microtubules (Fig. 2a,b, Supplementary Fig. 3a), we tested whether flubendazole affected JNK1-Bcl-2 phosphorylation and Beclin 1 activation. Treatment with flubendazole enhanced phosphorylation of JNK1 and Bcl-2 (Fig. 4a). In coimmunoprecipitation assays, flubendazole reduced Bcl-2 levels in Beclin 1 complexes (Fig. 4b) indicating release of Beclin 1 from its inhibitor Bcl-2 (ref. 38), allowing for Beclin 1 activation and autophagy initiation. Previous work^39^ has indicated that ATAT1 acetylase is required for efficient initiation of autophagy. Consequently, when we knocked down ATAT1, this prevented efficient JNK and Bcl-2 phosphorylation in response to flubendazole (Fig. 4c), and inhibited LC3 puncta induction in flubendazole-treated cells revealed by quantifying GFP-LC3 puncta in the presence of bafilomycin A1 (Fig. 4d).

In contrast to other microtubule depolymerizing drugs, which can increase early autophagosomal organelles but are not associated with efficient autophagic flux (nocodazole^40^,^41^; taxol^41^; vinblastine^40^,^41^; and vincristine^28^; Supplementary Table 3), flubendazole robustly induces autophagic maturation (Fig. 1g–k). Flubendazole treatment noticeably increases the acetylated microtubules (Fig. 2a,b, Supplementary Fig. 3a–c), which are important not only for autophagosome biogenesis but may also assist fusion of autophagosomes with lysosomes^36^,^37^,^41^. Accordingly, acetylated microtubules colocalized with autophagosomes even in flubendazole-treated cells (Supplementary Fig. 5b). Furthermore, ATAT1, which increased on flubendazole treatment (Fig. 2c), appeared to be required for efficient maturation/flux as suggested by accumulation of LC3-II in cells knocked down for ATAT1 on flubendazole treatment (Fig. 4e). Thus, by affecting the microtubules in a unique manner, flubendazole promotes both autophagy initiation and maturation, leading to induction of autophagic flux. Having established flubendazole’s mode of action in autophagy, we next tested the autophagy-modulating properties of flubendazole in relevant cellular systems modelling specific stages of several human diseases.

### Flubendazole blocks HIV-1 Dendritic cell to T-cell transfer

**Figure 5:**
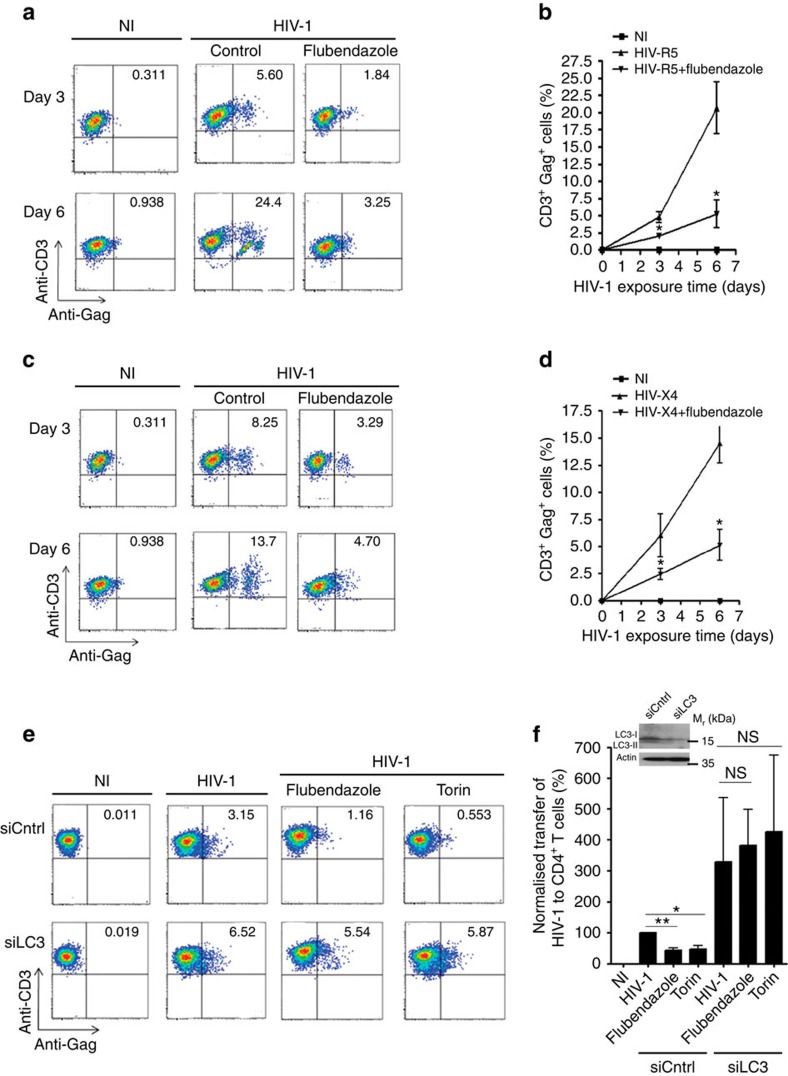
Flubendazole inhibits DC to CD4^+^ T-cell transmission of HIV-1. (**a**) Representative flow cytometry analysis of DC-CD4^+^ T-cell-mediated HIV-1 (R5) transfer (intracellular staining with anti HIV-Gag), 3 and 6 days after treatment with flubendazole. (**b**) Quanitification of cytometry analysis of DC-CD4^+^ T-cell-mediated HIV-1 (R5) transfer (as in **a**); mean±s.d. (*n*=3). (**c**) Representative fluorescence-activated cell sorting analysis for of DC-CD4^+^ T-cell-mediated HIV-1 (X4) transfer (intracellular staining with anti HIV-Gag), 3 and 6 days after treatment with flubendazole. (**d**) Quantification of DC-CD4^+^ T-cell-mediated HIV-1(X4) transfer (as in **c**); mean±s.d. (*n*=3). (**e**) siCtrl (control siRNA) DCs or siLC3 (LC3 siRNA) DCs were exposed overnight to HIV-1 (X4) after pre-treatment with flubendazole or mTOR inhibitor Torin (1 μM) acting as control. DC-CD4^+^ T cell HIV-1 transfer (intracellular HIV-1 Gag staining) normalized to 100% DC-CD4^+^ T+HIV-1. (**f**) Quantitation of data illustrated in panel (**e**); mean±s.d. (*n*=3). Inset: lysates of DCs transfected with siCtrl or transfected with siLC3 were immunoblotted with anti-LC3. LC3-I and LC3-II are indicated by arrows. Typical downregulation of LC3 expression in DCs with siLC3 ranged from 60 to 80%. Statistics: mean±s.e.; Student’s *t*-test or analysis of variance. *P< 0.05; **P< 0.01.

Autophagy may counter HIV infection^12^,^42^,^43^,^44^. We have previously reported that autophagy can block transfer of HIV-1 to CD4^+^ T cells^43^, a key step during early stages of infection with HIV^45^,^46^,^47^,^48^. We tested flubendazole for effects on HIV in a cellular model of infection (DC to T-cell transfer of HIV-1). Immature dendritic cells (iDCs) were treated with flubendazole and then infected with HIV-1. The infected iDCs were incubated with autologous CD4^+^ T cells, and DC to T-cell transfer of HIV-1 was measured by staining of CD4^+^ T cells with anti-Gag antibody. Flubendazole reduced DC to T-cell transfer of R5-tropic HIV-1 at multiple time points postinfection (Fig. 5a,b). Flubendazole was also effective in blocking DC to T-cell transfer of an X4-tropic HIV-1 (Fig. 5c,d).

To establish that flubendazole decreased DC-CD4^+^ T-cell-mediated HIV-1 transfer through autophagy, we studied effects of LC3 knockdown (Fig. 5e,f) using the previously reported reagents and approaches^43^. An efficient downregulation of LC3 expression of 60–80% with siRNA directed against LC3 was observed when compared with control siRNA-treated DCs (Fig. 5f). Knockdown of LC3 in DCs abrogated the inhibitory effects of flubendazole and Torin 1 (a selective ATP-competitive inhibitor of mTOR used as a positive control^49^ on HIV-1 transfer from DCs to T cells (Fig. 5e,f). Thus, flubendazole limits DC to T-cell transfer of HIV-1 in an autophagy-dependent manner.

**Figure 6:**
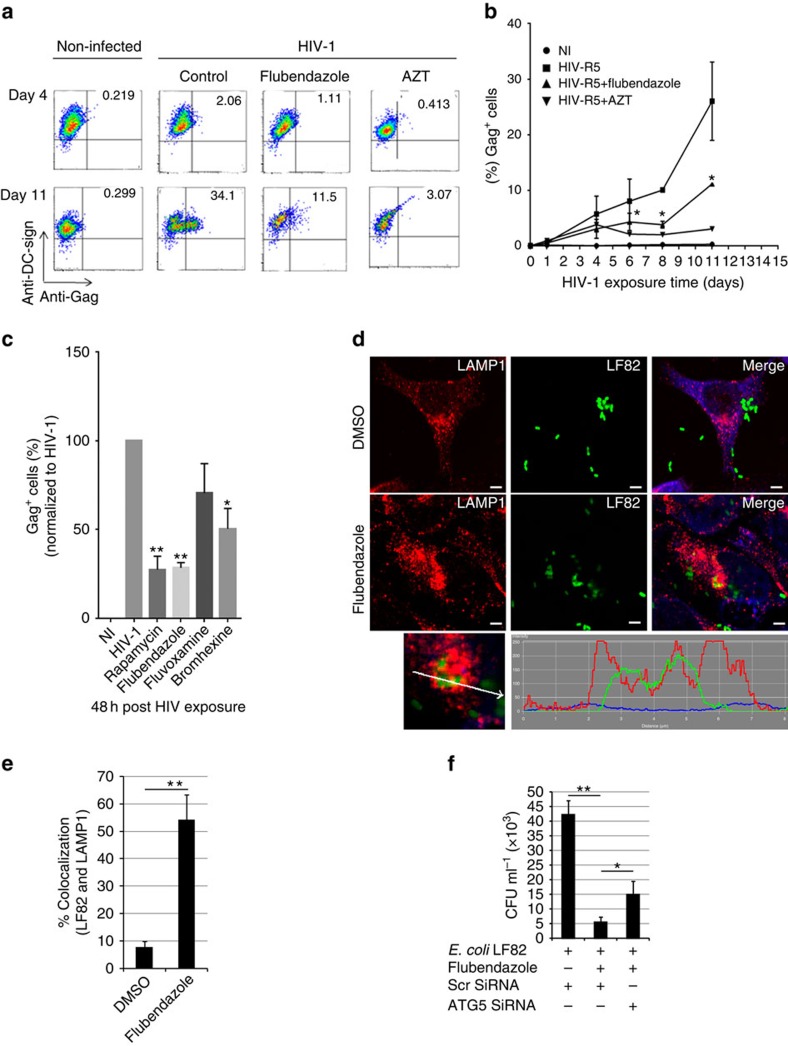
Flubendazole affects HIV-1 infection of DC and microbial killing. (**a**) Flow cytometry analysis of HIV-1 Gag levels in iDCs on day 4 and day 11 with (triangles) or without (squares) flubendazole or with AZT (inverted triangles) treatment. (**b**) Data (from three different donors), mean±s.d. (*n*=3). (**c**) HIV-1 R5 infected (p24, 250 ng) DCs; intracellular Gag levels at 48 h postinfection in DCs treated with flubendazole (2 μM), fluvoxamine (2 μM), bromhexine (20 μM), niclosamide (0.2 μM) or rapamycin (5 μM) as a control. Values normalized to DC+ HIV-1 infection at 100%. Data, mean±s.d. (*n*=3). (**d**) Confocal images showing the localization of adherent invasive *E.coli* LF82 (green) relative to anti-LAMP1 staining (red) in flubendazole-treated or untreated HeLa cells. (**e**) Percentage of intracellular *E. coli* LF82 (green) colocalized with lysosome marker protein LAMP1 (red) in absence or presence of flubendazole from 30 microscopic fields for each of control or treated cells±s.e. Student’s unpaired *t*-test was used to test for statistical significance *P<0.05. (**f**) Effects of flubendazole treatment (30 min, 5 μg ml^−1^) on intracellular survival of *E. coli* LF82 (multiplicity of infection (MOI) 1:20) at 1 h postinfection in scrambled or ATG5 siRNA transfected THP-1 cells. Results are expressed as mean±s.e. of CFU per ml. ANOVA was used to test for statistical significance. *P<0.05; **P< 0.01. Scale bars, 5 μm. CFU, colony-forming units; ANOVA, one way analysis of variance.

A subset of additional autophagy-inducing drugs from the screen was tested for efficacy against HIV-1 transfer from DC to T cells (Supplementary Fig. 6a–c). Flubendazole and rapamycin (included as a positive control) showed inhibitory effects on DC to T-cell transfer of HIV-1, whereas other autophagy inducers showed a trend but reached no statistical significance (Supplementary Fig. 6a–c). Flubendazole also decreased infection of DCs with the virus comparable to the effects of AZT (Fig. 6a,b). When compared with other autophagy-inducing drugs, flubendazole showed the strongest inhibition of DC infection with HIV-1, on par with that of rapamycin (Fig. 6c). Thus, the effect of flubendazole on DC to T-cell transfer can be explained in part by the effects of flubendazole on DC infection with HIV-1. However, flubendazole did not have a generalized effect on HIV-1 yields: although AZT blocked viral expression in a reporter cell line, flubendazole did not (Supplementary Fig. 6d). When bromhexine, another agonist of autophagy and autophagy flux identified in our screen (based on multiple assays; Fig. 1c, Supplementary Tables 1 and 2), was tested in the same assay, it too inhibited DC infection with HIV-1 (Fig. 6c).

### Flubendazole in autophagy of other microbiological targets

We tested whether flubendazole can promote clearance of other targets. Crohn’s disease (CD) is a chronic inflammatory syndrome linked through genome-wide association studies with autophagy^50^,^51^. The human population polymorphisms affecting autophagy^50^ influence its effector outputs including antimicrobial defence in multiple diseases^4^,^52^,^53^. Adhesive-invasive *Escherichia coli* (AIEC) LF82 has been associated with CD (ref. 54). When HeLa cells were infected with AIEC LF82, flubendazole treatment promoted delivery of *E. coli* to the lysosomal compartments, as revealed by LAMP1 colocalization (Fig. 6d,e; Supplementary Fig. 7a). Further, flubendazole treatment eliminated intracellular bacteria in an ATG5-dependent manner (Fig. 6f). This was not due to uptake differences, since the cells were first infected, treated with gentamycin to kill extracellular bacteria, and only then treated with flubendazole. Flubendazole had no effect on viability of AIEC LF82 when added to *E. coli* culture media (Supplementary Fig. 7b). Thus, flubendazole can clear diverse infectious agents through autophagy.

### Flubendazole and bromhexine can clear pathological tau

**Figure 7:**
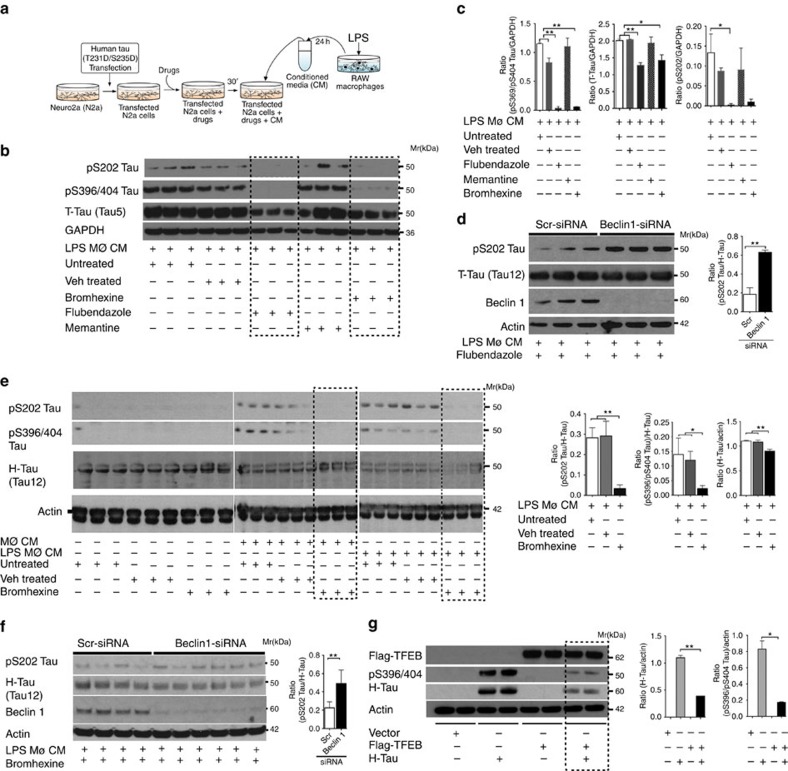
Flubendazole and bromhexine block inflammation-induced tau hyperphosphorylation. (**a–c**) N2a neuroblastoma cells were transfected with human tau (H-Tau) carrying T231D/S235D disease-associated phosphomimetic mutations and treated with CM (culture supernatant from RAW 264.7 macrophages activated with LPS) for 24 h in the presence or absence of autophagy agonists. Immunoblots for different forms of tau and phosphorylated states of tau: pS202 Tau, tau phosphorylated at Ser-202; pS396/404 Tau, tau hyperphosphorylated at Ser-396 and Ser-404 sites; H-Tau, transfected human tau (detected by Tau12 antibody); T-Tau (total, that is, mouse and human, tau detected by Tau5 antibody). (**d**) Analysis of phosphorylated tau levels in N2a cells knockdown for Beclin1 and treated with flubendazole. (**e**) Treatments as in **b**; lanes and blots as indicated (including human Tau revelaed with Tau12 antibody). (**f**) Analysis of phosphorylated tau levels in N2a cells knockdown for Beclin1 and treated with bromhexine. (**g**) Analysis of hyperphosphorylated (pS396/404) tau levels in cells cotransfected with mutant H-Tau (T231D/S235D) and Flag-TFEB in N2a cells. TFEB transfection was detected using anti-Flag antibody. Data, mean±s.e. (*n*=3); *P<0.05; **P<0.01; ANOVA Tukey’s multiple comparison *post hoc* test or unpaired *t-*test. ANOVA, analysis of variance.

We tested whether flubendazole can induce autophagy in another cell type and promote clearance of non-microbiological substrates. Autophagy has been linked with clearance of tau aggregates in models of Alzheimer’s disease (AD)^55^,^56^. We used neural cells and a two-pronged induction of tau aggregation (Fig. 7a). First, a phosphomimetic mutant (T231D/S235D) of human tau was overexpressed in Neuro2a (N2a) murine neuroblastoma cells. Second, inflammatory signals were provided to accentuate further tau phosphorylation (occurring on S202 and S396/S404), which in turn promotes further aggregation^57^. The inflammatory trigger was exposure of neural cells to conditioned medium (CM) from lipopolysaccharide (LPS)-activated macrophages (Fig. 7a). CM induced tau phosphorylation S202 and S396/S404 sites in N2a cells compared with untreated cells (Fig. 7b,c). Notably, N2a cells treated with LPS-stimulated macrophage CM showed a higher level of tau phosphorylation (Fig. 7b,c). Addition of flubendazole cleared hyperphosphorylated (pS396/S404) tau (Fig. 7b,c). The reduced tau in flubendazole-treated cells was not due to reduced cell viability or increased plasma membrane permeability measured by trypan blue exclusion (Supplementary Fig. 8a) or LDH release (Supplementary Fig. 8b). Flubendazole-induced clearance of hyperphosphorylated tau required Beclin 1 (Fig. 7d). These data indicate that flubendazole, a newly identified autophagy agonist, helps ameliorate pathological tau in this model system.

Bromhexine, another autophagy-inducing drug introduced in previous sections, was tested in parallel with flubendazole. N2a cells treated with bromhexine showed marked reduction in hyperphosphorylated (pS202 and pS396/S404) tau (Fig. 7b,c). Both flubendazole and bromhexine reduced the total levels of tau (Fig. 7b,c; detected by Tau5 antibody). The reduction in phosphorylated tau and total tau levels was statistically significant in flubendazole or bromhexine-treated N2a cells (Fig. 7c). Memantine, another central nervous-system active pharmaceutical (a glutamate receptor antagonist and a drug used in AD) identified by LC3 puncta as an inducer of autophagy (Supplementary Fig. 2), but not passing all secondary tests (Supplementary Table 2), did not clear hyperphosphorylated tau and did not reduce total tau (Fig. 7b,c).

Bromhexine cleared tau phosphorylated on S202 (Fig. 7b,c). Moreover, bromhexine cleared human tau, detected specifically using Tau 12 antibody recognizing only human tau in the overexpressing N2a cells (Fig. 7e). The reduced tau in bromhexine-treated cells was not due to increase in cell death or plasma membrane permeability (Supplementary Fig. 8c,d). Next, we followed the effect of bromhexine superimposed on a time course of CM-dependent induction of tau hyperphosphorylation (Supplementary Fig. 8). Bromhexine showed consistent and statistically significant increase in phospho-tau clearance over the time course (Supplementary Fig. 8e–i). To test whether bromhexine-induced clearance of tau was through autophagy, we knocked down Beclin 1 in N2a cells, and this prevented phospho-tau and human tau clearance (Fig. 7f). In addition to the above mechanistic analysis with Beclin 1 knockdowns, overexpression of TFEB^33^ resulted in clearance of phosphorylated and human tau (Fig. 7g), in keeping with recently published data^56^. Although TFEB did not reduce total tau (Supplementary Fig. 8j), its overexpression cleared human hyperphosphorylated tau in N2a cells (Fig. 7g), thus validating our model system as responsive to autophagy-based clearance of tau. In conclusion, induction of autophagy with flubendazole, bromhexine or TFEB expression can lead to reduced pathological tau in N2a cells.

## Discussion

**Figure 8:**
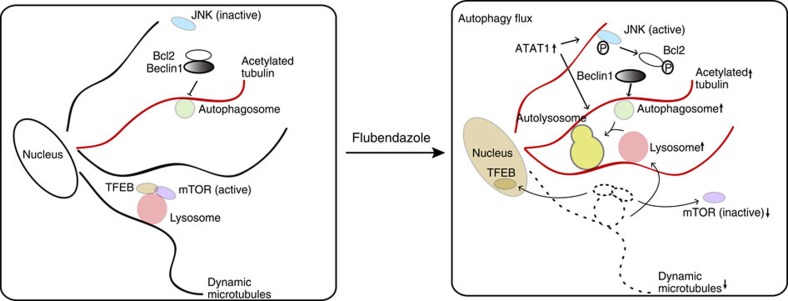
Model of flubendazole-induced autophagy and autophagy flux. Schematic on the left: under basal conditions, mTOR residing on lysosomes keeps TFEB inactive in the cytoplasm. Bcl-2 interacts with and blocks Beclin 1 activity. Both of these processes suppress autophagosome biogenesis. Schematic on the right: Treatment with flubendazole disrupts dynamic microtubules leading to mTOR displacement from lysosomes and its inactivation; mTOR inactivation causes nuclear translocation of TFEB. In parallel, flubendazole increases cellular ATAT levels and acetylated microtubules, which (**a**) promotes phosphorylation of Bcl-2 by JNK1 to disinhibit Beclin 1 and (**b**) provides tracks for transport of autophagosomes and lysosomes promoting autolysosome formation. These flubendazole-initiated processes collectively induce autophagy and promote conversion to autolysosomes thus activating the entire autophagic pathway from its initiation to its maturation stages.

Our data show that flubendazole, a compound that passed all stages of our screen for inducers of autophagy among pharmaceuticals, activates autophagy and autophagy flux by affecting multiple points of the autophagic pathway. This includes Beclin 1, mTOR and TFEB, as well as ATAT1 as an effector of flubendazole action that promotes both induction and maturation of autophagosomes dependent on the above factors (Fig. 8). Our findings suggest that it is the concerted action of these seemingly disparate effects of flubendazole, with many of them being anchored on microtubules, which engage the principal regulators of autophagy and bring about strong activation of autophagy and autophagic flux (Fig. 8). TFEB is one of the above regulators of autophagy affected by flubendazole, and may participate in the action of another drug, bromhexine, as discussed below. The coordinated activation of multiple autophagy regulatory systems was found to be the underlying cause for strong activation of autophagy and autophagic flux observed with flubendazole in follow-up mechanistic assays and biological outputs with diverse substrates.

The autophagy pathway^1^ has a broad disease-treatment potential that is yet to be realized. Numerous screens of small molecule libraries have been conducted for compounds that modulate autophagy^3^,^10^,^14^,^15^,^58^,^59^,^60^,^61^. These screens have been carried out in many cases with the aim of repurposing existing drugs. We have used here the known drugs to uncover new signalling pathways that can be further exploited in autophagy drug targeting and discovery. Our screen has identified flubendazole as one of the more potent autophagy inducers in our hands. The study of its mechanism of autophagy induction uncovered that its potency involved simultaneous induction of several known systems converging on the autophagy pathway. This coordinated induction of autophagy and autophagic flux was based on flubendazole’s unique effects on microtubules that both promoted disruption of dynamic microtubules and at the same time stabilized acetylated microtubules. This not only promoted induction of autophagy but also caused effective maturation of autophagic organelles, thus activating a complete autophagic pathway from its initiation to maturation. This property was associated with clearance of diverse substrates in diverse cell types or blocking disease-associated processes *in vitro*: interruption of a key process of HIV transmission, the transfer of the virus from DCs to T lymphocytes, reduction in HIV infection of DCs, elimination of CD-associated invasive bacteria from infected epithelial cells, and removal of a pathological form of tau from inflamed neural cells. The effects of microtubule dynamics on autophagy^36^,^37^, a somewhat less studied relationship in the mainstream studies of autophagy, should receive much more attention in future autophagy drug development. Furthermore, regulation of acetyltransferases, such as ATAT1 studied here, along with deacetylating enzymes deserves further attention given that very recent studies indicate that deacetylation and acetylation of the core autophagy proteins Beclin 1 and LC3 significantly modulates autophagy^62^,^63^.

In addition to flubendazole, bromhexine was found to induce effective clearance of an important autophagic substrate, hyperphosphorylated tau^64^. This property of bromhexine is likely related to its effects (or the action of its metabolite ambroxol) on trafficking and activity of the lysosomal enzymes glucocerebrosidase or glucosylceramidase to the lysosome^66^. Ambroxol has recently been linked to TFEB activation^67^, and thus this is the likely pathway acting on bromhexine stimulation. The two major hits in our study affect the lysosomal system and engage TFEB activation, and thus both already provide autophagy-inducing drugs that promote TFEB activation and may be candidates for repurposing in diseases where this is advantageous.

The therapeutic promise of autophagy in a variety of human diseases^3^,^4^,^7^,^8^,^10^,^11^,^15^ beckons for quick development of treatments by repurposing the existing drugs. However, caution must be taken, and expectations for therapeutic index, that is, benefits versus drug safety, need to be taken into account when initiating and interpreting clinical trials aimed at using existing drug for repurposing. For example, the known low toxicity of flubendazole in several studies^68^ (unless combined with a potent cytotoxic drug^21^), suggest that it could be considered for further development. Our data indicate that activation of the autophagolysosomal system via TFEB- and/or MiT/TFE-inducing drugs with pharmacologies revealed in our study will be a productive direction in customizing the next generation of more tailored autophagy drugs.

## Methods

### Screening of small molecule libraries

Screening of small molecule libraries for their autophagic regulatory capacities was conducted by using a CellomicsArrayScan to quantitate LC3-GFP/red fluorescent protein (RFP) puncta in HeLa cells. HeLa cells stably transduced with LC3-GFP/RFP tandem construct generate fluorescent autophagic puncta, which are either green/yellow (early autophagosomes) or red (late autophagosomes fused with lysosomes, which degrades the GFP). By quantitating the number or total area of fluorescent puncta per cell, detection of alterations in autophagic flux and regulation were observed.

Before any actual screening, preliminary experiments were conducted. One question was which parameter was most sensitive for detecting changes in autophagy. We tested total puncta area, number, or intensity per cell for both GFP^+^ and RFP^+^ puncta after treatment with pp242, an mTOR inhibitor and known inducer of autophagy. GFP^+^ puncta area was the most distinct readout for induction of autophagy, although other parameters in both the RFP and GFP channels also showed changes. Other methods of induction of autophagy were tested for use as positive controls, including starvation and the mTOR inhibitor rapamycin. However, pp242 was consistently the stronger inducer of autophagy in this assay, and was used as a control in subsequent screens.

Using the above system, we performed two separate screens on the Prestwick Chemical Library, the Microsource Spectrum 2000 library and the Johns Hopkins library, which contain FDA-approved compounds, bioactive molecules, and natural products, and represent wide functional and chemical diversity. Cells were plated at 5,000 cells per well of 384-well plates overnight. Compounds were added at a final concentration of10 μM using BioTek robotics, incubated for 4 h at 37 °C, fixed with a total concentration of 0.1% paraformaldehyde (PFA), and placed at 4 °C overnight. Each plate had 32 DMSO-only controls and 32 pp242 controls. GFP puncta area or number per cell was than assessed as above. A hit was defined as three s.d. above or below the mean of the negative control wells on a given plate. After two separate screens, compounds that scored as hits on both screens were analysed further. Images were manually scanned for autofluorescence, precipitation or apoptosis, and these false positives were removed from further analysis. Dose responses for each putative inducer compound were compared with the potent inducer of autophagy pp242. Two separate dose-response (in nM–μM range) experiments were performed on hits obtained from the screens.

Follow-up tests used LC3-II conversion in the presence of bafilomycin A1 (refs 17, 19) with statistical analysis in three or more independent experiments being the filter for inclusion in subsequent biological assays.

### HIV assays

Human monocytes from buffy coats were obtained in accordance with institutional guidelines of the ethical committee of the University of Cardiff. Monocytes were purified after Ficoll gradient separation with CD14 MicroBeads (MiltenyiBiotec). Usual purity was>95% CD14^+^. Human monocyte derived dendritic cells (MDDCs) were generated by incubation of purified monocytes in IMDM supplemented with 10% FCS, 2 mM L-glutamine, 100 IU ml^−1^ penicillin, 100 μg ml^−1^ streptomycin, 10 mM HEPES, 1% non-essential amino acids, 1 mM sodium pyruvate, 500 IU ml^−1^ granulocyte–macrophage-colony-stimulating factor and 500 IU ml^−1^ IL-4 (both supplied by Strathman Biotec, Germany). On days 2 and 4, a third of the culture medium was replaced with fresh IMDM medium supplemented with granulocyte–macrophage-colony-stimulating factor and IL-4. At day 6 immature DCs were harvested and analysed by flow cytometry. Autologous CD4^+^ T-lymphocytes were purified with CD4^+^ T-cell isolation kit II (MitenyiBiotec) in accordance with the manufacturer’s instructions. Hut-CCR5 cell line and autologous CD4^+^ T-lymphocytes were maintained in supplemented RPMI1640. 293 T human embryonic kidney cells and HeLa P4-R5 (NIH AIDS Research & Reference Reagent Program) cells were maintained in supplemented DMEM.

Virus stocks were produced as previously described^69^ with 293 T cells transiently transfected with calcium-phosphate coprecipitated proviral plasmid pR9 or pR8-Bal, encoding for full-length HIV-1 X4 and HIV-1 R5 strain provirus respectively. Infectious titres of viral stocks were evaluated by limiting dilution on HeLa P4-R5 cells. Viral titres were expressed as infectious units (IU) per ml. Approximately 500 ng of p24 ^gag^ on 2.5 × 10^5^ CD4^+^HeLa P4-R5 corresponds to a MOI of 1. Physical tires were also evaluated by quantification of an HIV-1 p24^gag^ by ELISA kit (Beckman Coulter, Paris, France). capture and transfer assays, 5 μM Rapamycin (Merck Chemicals Ltd), 50 μg ml^−1^ Zidovudine (AZT; GlaxoSmithKline) and 1 μM Torin (R&D Systems) were used.

Antibodies against DC-SIGN, CD3-PE, CD3-APC and fluorescence-activated cell sorting antibodies were obtained from BD Transduction Laboratories (Franklin Lakes, NJ). Anti-LC3B (PM036) used for immunoblotting was from MBL International (Woburn, MA). Anti-actin was from Sigma (St Louis, MO). Monoclonal anti-HIVgag (KC57-FITC from Beckman Coulter, Miami,FL) was used for flow cytometry analysis.

For knockdowns in HIV experiments, siLC3-α (sc-106197), siLC3-β (sc-43390) and siCtrl were purchased from Santa Cruz Biotechnology (Santa Cruz, CA). HiPerFect Transfection Reagent (Qiagen, Hilden, Germany) was used for transfection in accordance with the manufacturer’s recommendations. 4 × 10^5^ iDCs were transfected with 200 nM siRNA in 500 μl of IMDM/0.5% FCS medium in 12-well plates. A second round of transfection was performed 24 h later. Specific gene knockdowns were assessed by immunoblotting followed by densitometry analysis (Quantity One; Bio-Rad Laboratories, Hercules, CA) comparing with non-transfected and si Ctrl conditions.

For degradation assay, 10^5^ of iDC were plated in 96-round-well plates in 100 μl final volume of IMDM and pretreated or not with drugs Flubendazole (2 μM), Fluvoxamine (2 μM), Bromhexine (20 μM), Niclosamide (0.2 μM) or Rapamycin (5 μM) for 3–4 h. Cells were washed and challenged overnight with HIV-R5 strain R8Bal (250 ng HIV-p24). Cells were harvested after 48 h, fixed in 2% PFA, permeabilized and stained with anti-HIV-Gag-FITC and anti-DC-SIGN-APC. Experiments were done in triplicates (*N*=3).

For virus transfer assay, 10^5^ of iDC were plated in 96-round-well plates in 100 μl final volume of IMDM and pretreated or not Flubendazole (2 μM), Fluvoxamine (2 μM), Bromhexine (20 μM), Niclosamide (0.2 μM) or Rapamycin as a control (10 μM) for 3–4 h. Cells were then challenged overnight with HIV-1 (MOI of 1 corresponding to 250 ng HIV-p24 for HIV-R5 (strain AD8) or 250 ng HIV-p24 for HIV-X4 (strain R9). Cells were washed and cocultured with 1 × 10^5^ autologous CD4^+^ T cells for 4 days or days 3 and 6 for assay with Flubendazole only. Cells were harvested, fixed in 2% PFA, permeabilized and stained with anti-HIV-Gag-FITC and anti-CD3-PE. Experiment representative of *N*=3 experiments.

For viral replication assay, 10^5^ of iDC were plated in 96-round-well plates in 100 μl final volume of IMDM and pretreated or not with drug flubendazole (2 μM) for 3–4 h. AZT (1:200) was used as a control of viral replication and iDCs were pretreated for 3–4 h. Cells were washed and challenged overnight with HIV-R5 strain R8Bal (250 ng HIV-p24). Cells were harvested after 4 and 11 days, fixed in 2% PFA, permeabilized and stained with anti-HIV-Gag-FITC and anti-DC-SIGN-APC. Experiments were done in triplicates (*N*=3).

For viral detection by flow cytometry, after isolation or differentiation, surface staining of primary cells (monocytes, MDDCs and autologous CD4^+^ T cells) were performed at 4 °C for 30 min with monoclonal antibodies from BD and directed against the following molecules: CD1a, CD3, CD4, CD8, CD14, CD16, CD19, CD25, CD45Ro, CD69, CD83, CCR7, DC-SIGN and HLA-DR. Gag p24 expression was measured on permeabilized cells with anti-Gagp24 (FITC-coupled) mAb (KC57, Coulter). Isotope-matched monoclonal antibodies were used as negative controls. Samples were analysed by flow cytometry with a FacsCanto (Becton Dickinson) and data processed with FlowJo software.

### Microscopy analyses and quantification

Cells were visualized using a laser confocal microscope and images were captured using LSM510 software^42^. For quantification of nuclear translocation of TFEB and colocalization of LF82 and LAMP1, images from different fields were captured and profiles analysed^42^.

### Microtubule immunostaining and imaging

For microtubule staining, cells were fixed in formaldehyde (5 min) followed by 10 min permeabilization with 0.01% TritonX-100, 1% BSA in phosphate-buffered saline (PBS) and blocking in 1% BSA in PBS. Abcam antibody ab6161 was used for immunostaining of α-tubulin. Acetylated tubulins were stained with anti-acetylated monoclonal antibody from Sigma (clone 6-11B-1, cat # T7451). Immunostained cells were imaged by confocal microscopy, with constant parameters for excitation, emission, pinhole and exposure time when levels of immunostained material were compared.

### Tau protein accumulation assays

Mouse neuroblastoma cells (Neuro2a or N2a cells; ATCC, Manassas, VA) were cultured in in Neurobasal media for up to 10 passages before being expanded and plated at a density of 500,000 cells per well for all the experiments. The mouse RAW 264.7 macrophage cells (ATCC TIB-71) were maintained in 10% fetal bovine serum (FBS)/DMEM for up to 10 passages before being expanded into two flasks.

For treatment of N2a cells with flubendazole, bromhexine, or memantine and macrophage conditioned media (CM); the media in each flask of RAW cells were replaced with Neurobasal media containing either vehicle (RPMI) or LPS (1 ng ml^−1^). RAW cells were activated with LPS for 18 h. Before the conditioned media experiment, N2a cells were transiently transfected with human tau plasmid (0.4 mg DNA per transfection), pRC/CMV n123c (0N3R tau) containing T231D/S235D mutation (tau^T231D/S235D^), which mimic phosphorylation on this disease-specific site^70^ using Effectine reagent per manufacturer’s instructions (Qiagen, Cat# 301425). Tau-transfected N2a cells were pretreated for 30 min with vehicle (DMSO), flubendazole (5 μg ml^−1^), bromhexine (60 μM), memantine (30 μM); or left untreated. After 30 min, human tau-expressing N2a cells were treated with either vehicle- or LPS-treated RAW CM for 6 h (for flubendazole) or 24 h (for bromhexine and memantine). The cells were rinsed with PBS three times, lysed and processed for SDS–polyacrylamide gel electrophoresis and western blotting with 4–12% Bis-Tris protein Gel (Life Technologies). The membranes were probed for; 1) tau with AT8 antibody to detect phosphorylated tau at S202/T205 at 1:5000 (Thermo Scientific); PHF1 antibody to detect phosphorylated tau at S396/S404 at 1:5000 (a kind gift from Dr Peter Davies); Tau5 antibody (ab80579) to detect both human and mouse tau at 1:5000; Tau12 antibody (ab74137) to detect only human tau at 1:5000 for both (Abcam); followed by incubations with respective secondary antibodies (Jackson Immunoresearch) and developed with enhanced chemiluminescence (ECL; Pierce).

For the time-course kinetic experiment, the N2a cells and RAW cells were maintained and set up as above. N2a cells were transiently transfected with human tau^(T231D/S235D)^ before pre-treatment with Bromhexine for 30 min or DMSO. RAW cells were stimulated with LPS (1 ng ml^−1^). LPS-stimulated RAW CM was added to the tau-transfected, Bromhexine (60 μM) treated, N2a cells. The cells were lysed at the following time points: 0, 1, 2, 3, 4, 6 and 8 h post-LPS-CM stimulation. Cells from each treatment were lysed in triplicate.

For the Beclin 1-silencing experiments, the N2a cells expressing tau^T231D/S235D^ were nucleofected with siRNA to Beclin 1 and treated with LPS-activated macrophage CM. After 24 h, cells were lysed to detect phosphorylated tau, total human tau and Beclin 1.

For cotransfection of tau and TFEB, N2a cells were transiently transfected with a blank vector, tau^T231D/S235D^only, TFEB only (FLAG tag^33^; a kind gift from Dr Andrea Ballabio), or with both TFEB and tau^T231D/S235D^ plasmids. After 24 h, cells were to detect phosphorylated tau, autophagy and total human tau. Transfection of TFEB was detected by probing membranes with anti-FLAG antibody (Clone M2, Sigma, F1804; 1:1,000.

### Bacterial infection assays

For AIEC LF82 survival assay^54^, THP-1 cells were infected with AIEC LF82 of MOI of 1:20 for 1 h. To elminate extracellular *E.coli*, THP-1 cells were treated with gentamycin (100 μg ml^−1^) for 1 h followed by incubation in fresh media containing DMSO or flubendazole (5 μg ml^−1^) for 30 min. Cells were lysed and surviving bacteria quantified by plating and determining colony-forming units.

### Protein interactions analyses and immunoblotting

Protein interactions were analysed by coimmunoprecipitation assays. Briefly, the cells were lysed using NP-40 buffer (Invitrogen) containing protease inhibitor cocktail (Roche) and phenylmethylsulfonyl fluoride (PMSF). Lysates were incubated with antibody for 2 h followed by incubation with protein G Dynabeads (Life technologies) for 2 h. Beads were washed for four times with 1XPBS and then boiled with SDS–polyacrylamide gel electrophoresis buffer for analysis of interacting protein by Immunoblotting.

### Antibody and dilutions

Antibodies used in this study: ATAT1 (Abcam, ab58742); LC3B (Sigma, L7543); HDAC6 (Cell Signaling, #7558); JNK1/p-JNK (Cell Signaling, #9252, #4668); Bcl-2/p-Bcl2 (Cell Signaling, #2870, #2827); LAMP1 (Abcam, ab24170); S6K, pS6K (Cell Signaling, #9202, #9205); 4E-BP1, p4E-BP1 (Cell Signaling, #9644, #2855). For immunoblotting, primary antibodies were used in 1:1,000 dilution, and for immunofluorescence imaging as 1:100-1:200 dilution, unless specified differently. Secondary antibodies were at 1:1,000 dilution. Un-cropped blots are shown in Supplementary Figs 9–13.

### siRNA knockdowns

SMARTpool siGENOME siRNAs from GE Dharmacon were used for knockdowns.

### Statistical analysis

Significant differences between groups were calculated with analysis of variance or Student’s *t*-test; *P* values <0.05 were considered significant

## Additional information

**How to cite this article:** Chauhan, S. *et al.* Pharmaceutical screen identifies novel target processes for activation of autophagy with a broad translational potential. *Nat. Commun.* 6:8620 doi: 10.1038/ncomms9620 (2015).

## Supplementary Material

Supplementary Information Supplementary Figures 1-13, Supplementary Tables 1-3 and Supplementary References
