# Supplementary material for: Pharmaceutical screen identifies novel target processes for activation of autophagy with a broad translational potential
Source: Nat Commun. 2015 Oct 27;6:8620. doi: 10.1038/ncomms9620 (PMC4624223; doi:10.1038/ncomms9620)
Supplement: Supplementary Information — Supplementary Figures 1-13, Supplementary Tables 1-3 and Supplementary References [file ncomms9620-s1.pdf]

Supplementary Figure 1

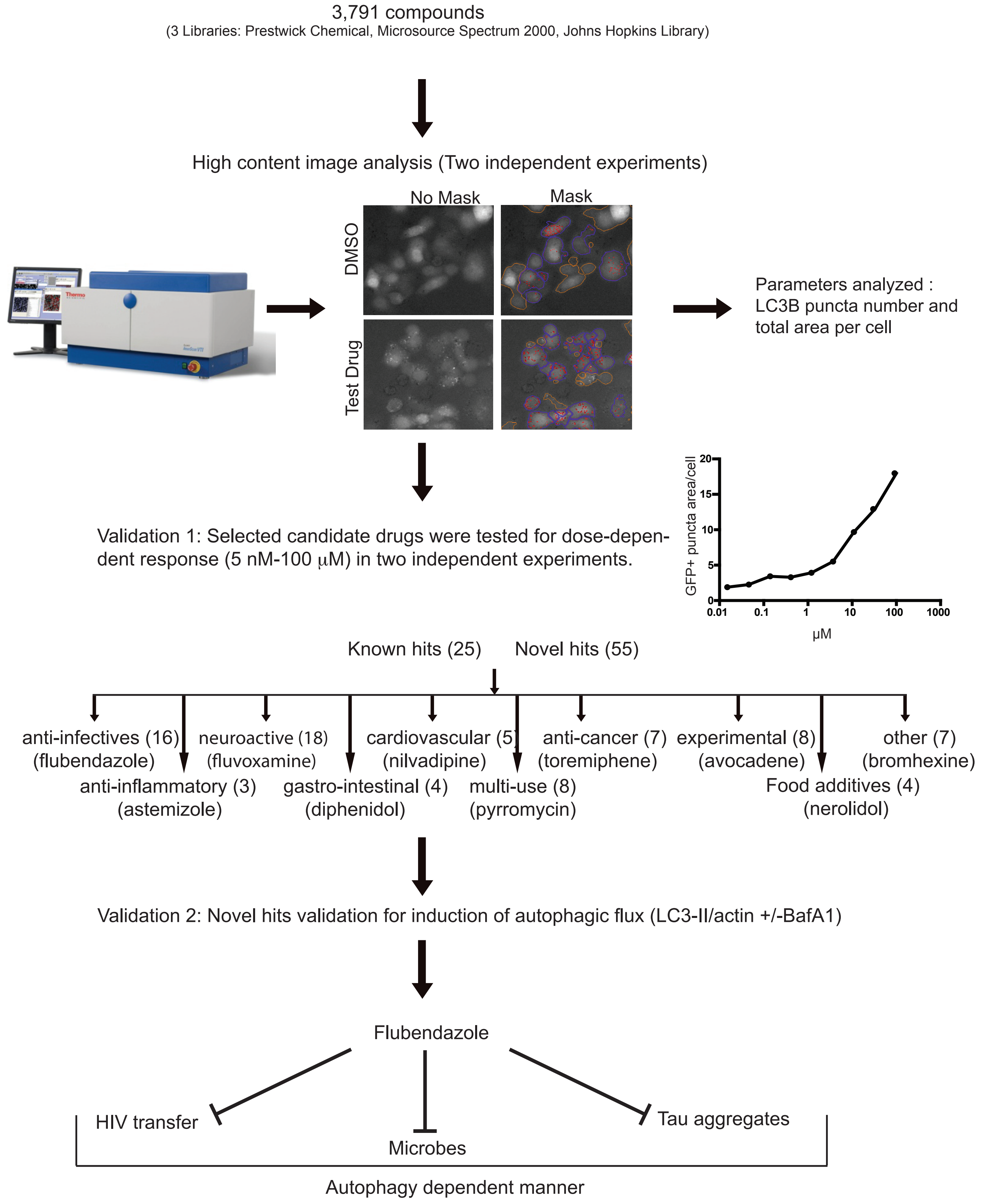

Supplementary Fig. 1. Screen workflow. Schematic representation of methodology and workflow used to screen and validate autophagy modulating drugs.

Supplementary Fig. 2. Dose response analysis of autophagy-modulating drugs. The compounds positive in LC3B puncta-based primary screens that passed the filters were tested for dose response in two independent experiments (high content image analysis).

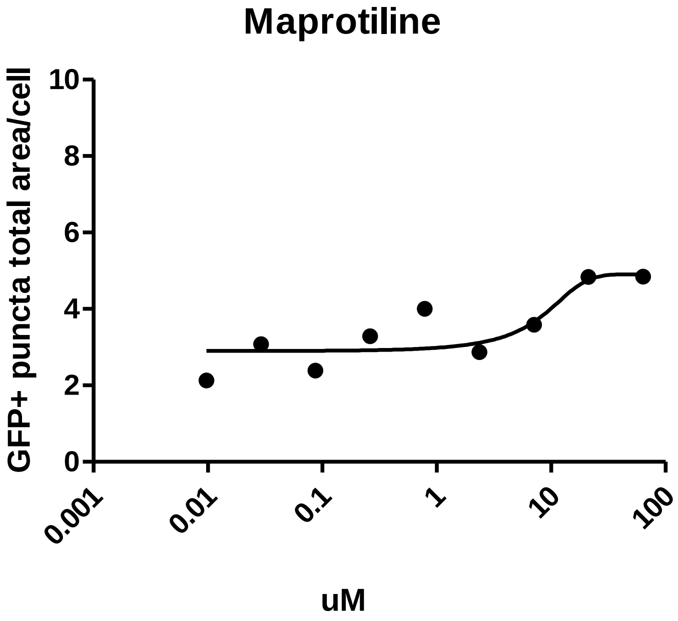
Maprotiline
GFP+ puncta total area/cell
uM

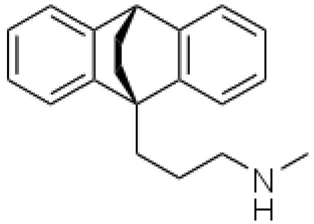
N
H

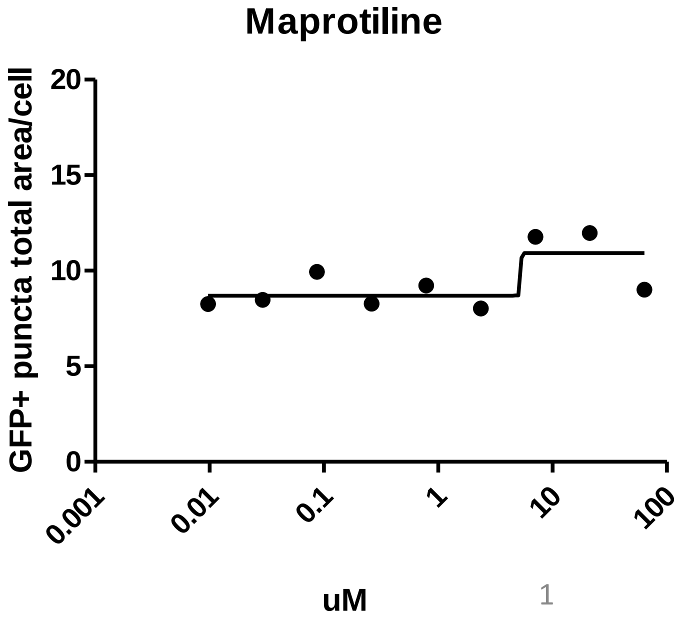
Maprotiline
GFP+ puncta total area/cell
uM

1

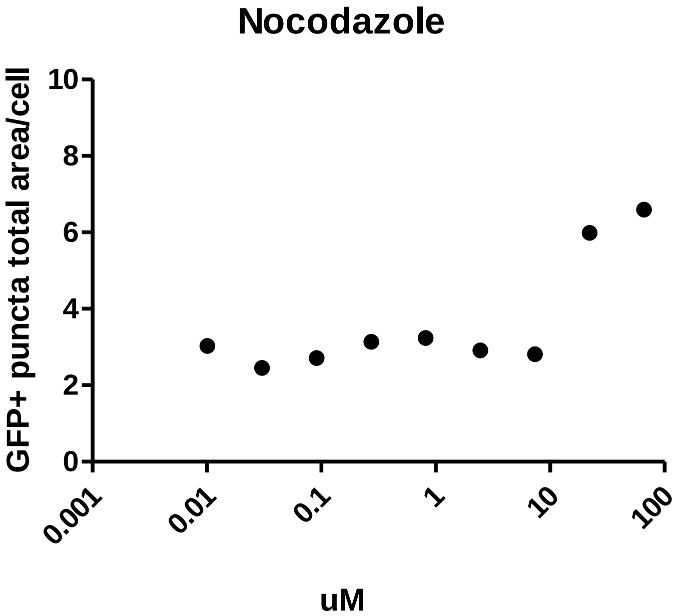
Nocodazole
GFP+ puncta total area/cell
uM

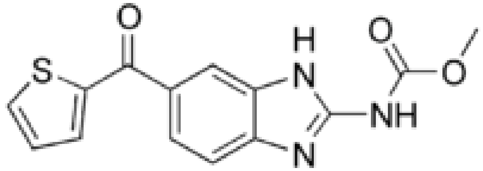
O
S
H
N
N
O
O
NH

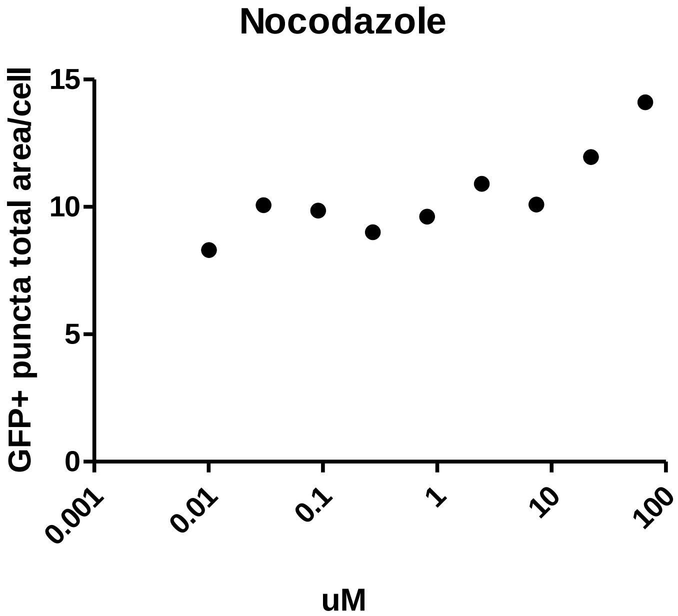
Nocodazole
GFP+ puncta total area/cell
uM

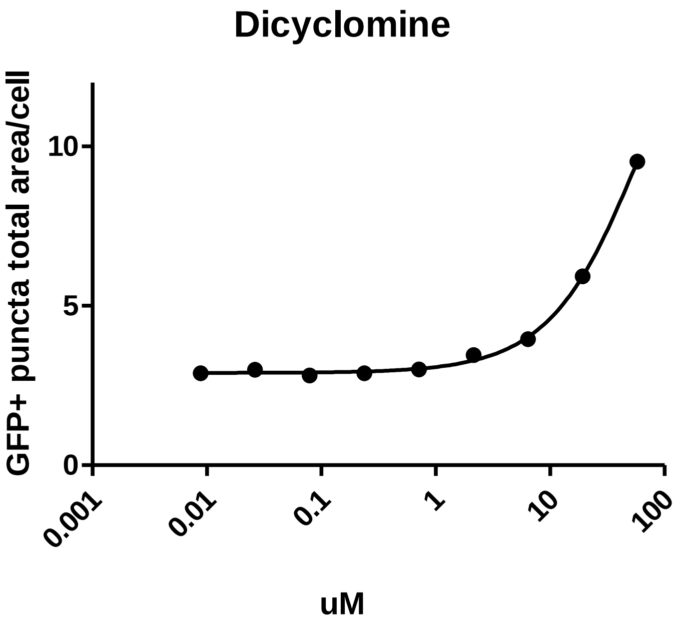
Dicyclomine
GFP+ puncta total area/cell
0
5
10
0.001
0.01
0.1
1
10
100
uM

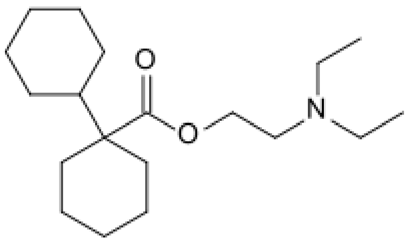
O
O
N

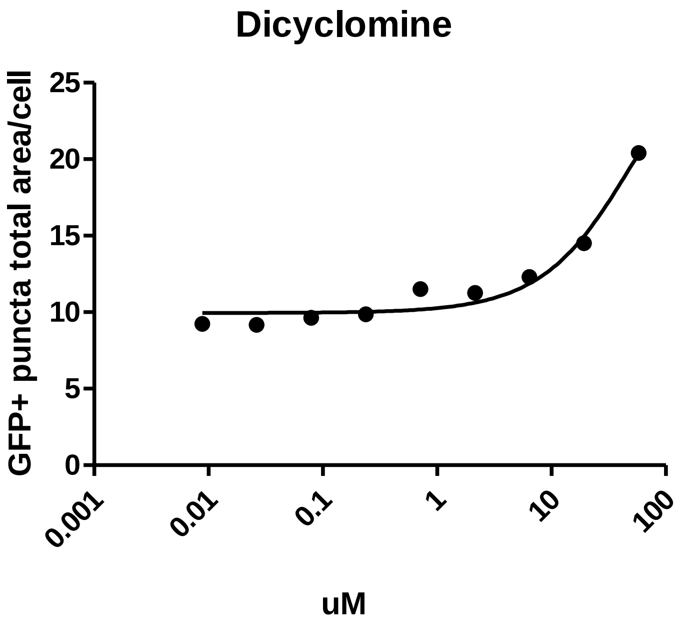
Dicyclomine
GFP+ puncta total area/cell
0
5
10
15
20
25
0.001
0.01
0.1
1
10
100
uM

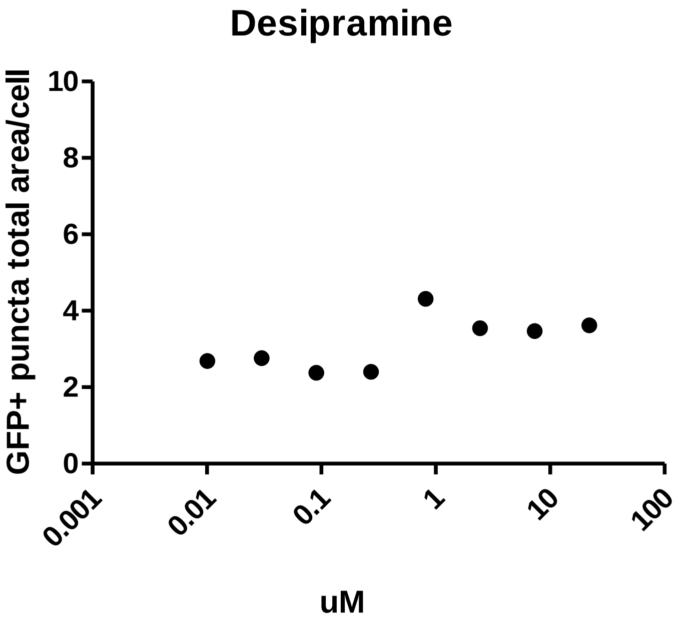
Desipramine
GFP+ puncta total area/cell
0
2
4
6
8
10
0.001
0.01
0.1
1
10
100
uM

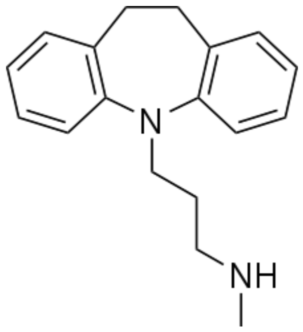
N
NH

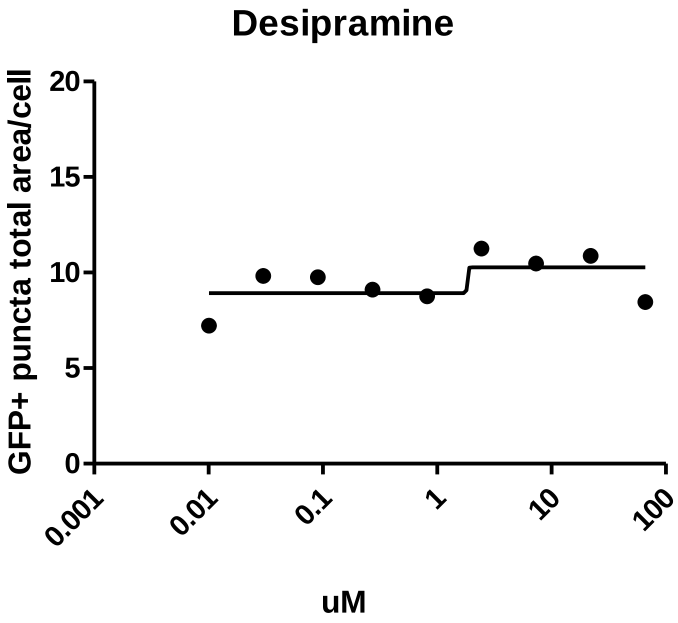
Desipramine
GFP+ puncta total area/cell
0
5
10
15
20
0.001
0.01
0.1
1
10
100
uM

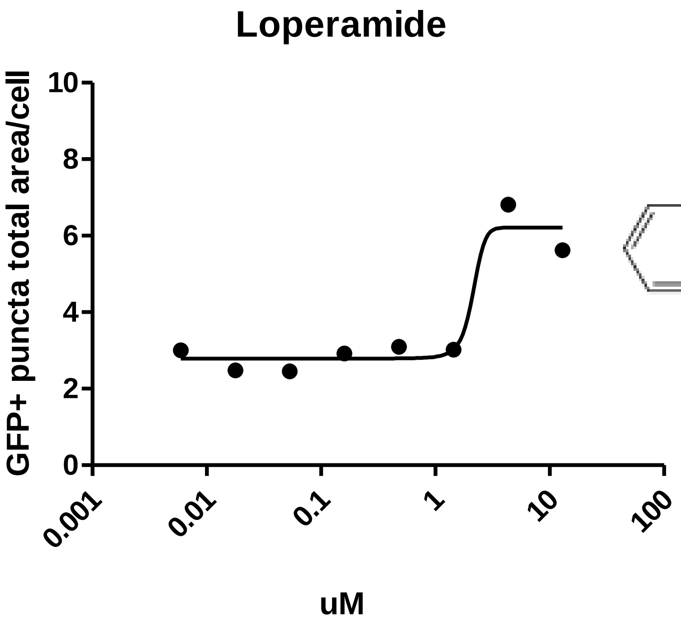
Loperamide
GFP+ puncta total area/cell
0
2
4
6
8
10
0.001
0.01
0.1
1
10
100
uM

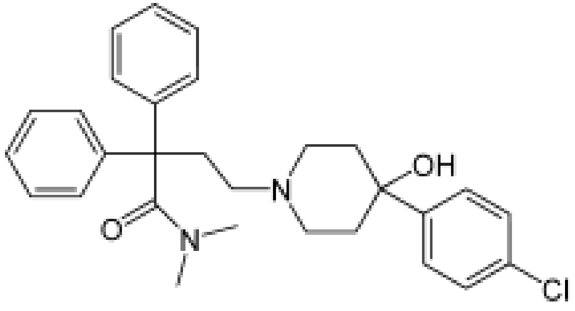
OH
N
O
N
Cl

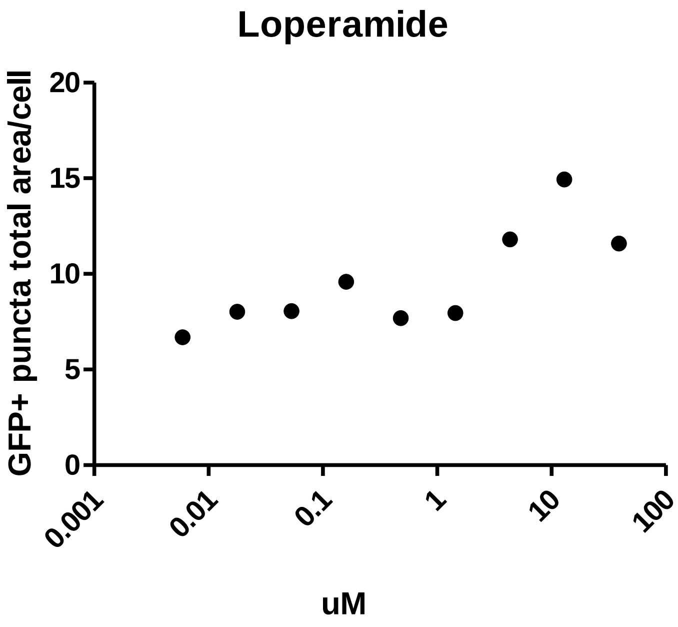
Loperamide
GFP+ puncta total area/cell
0
5
10
15
20
0.001
0.01
0.1
1
10
100
uM

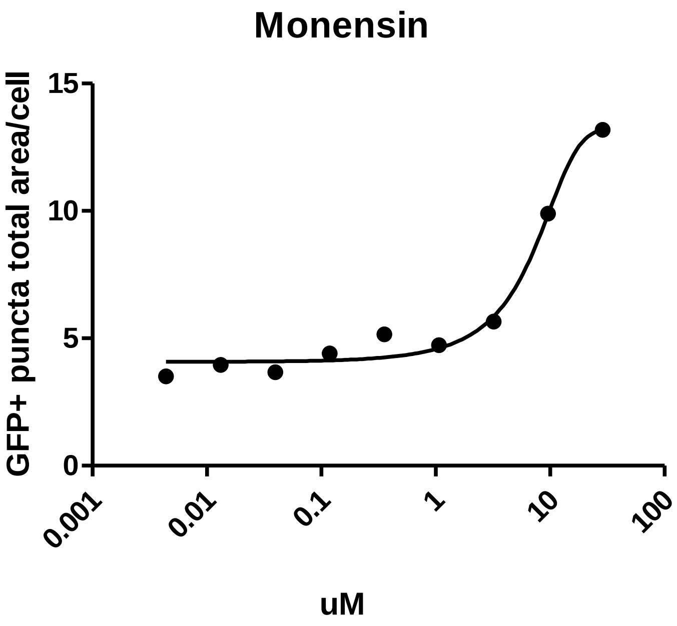
Monensin
GFP+ puncta total area/cell
0
5
10
15
0.001
0.01
0.1
1
10
100
uM

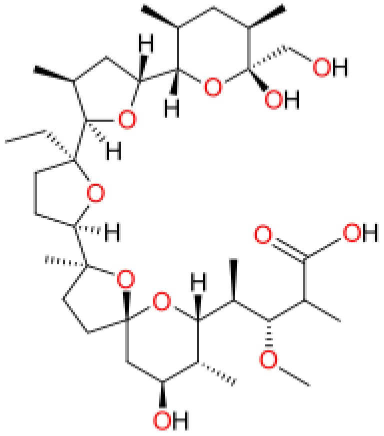
OH
OH
O
OH
OH
O
OH

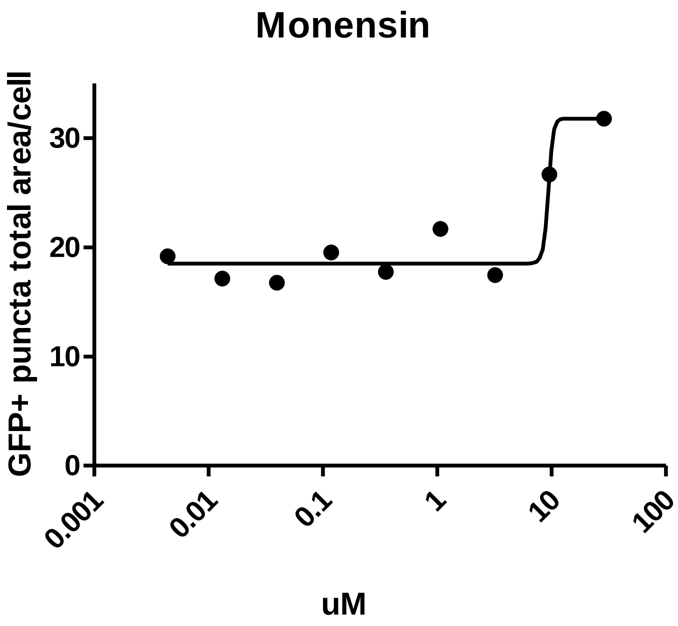
Monensin
GFP+ puncta total area/cell
0
10
20
30
0.001
0.01
0.1
1
10
100
uM

Sertraline

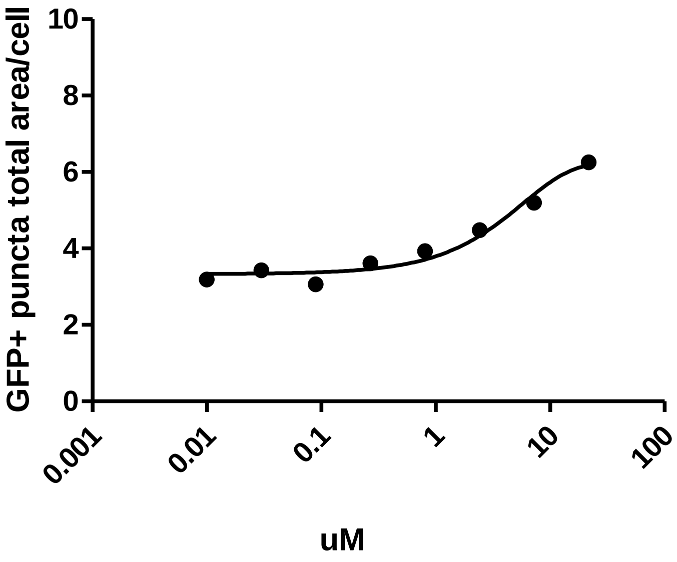

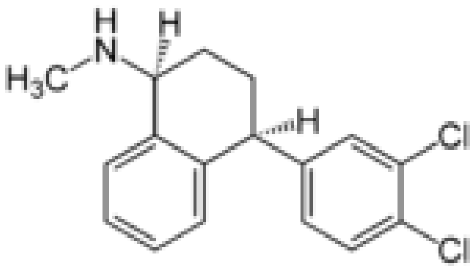

Sertraline

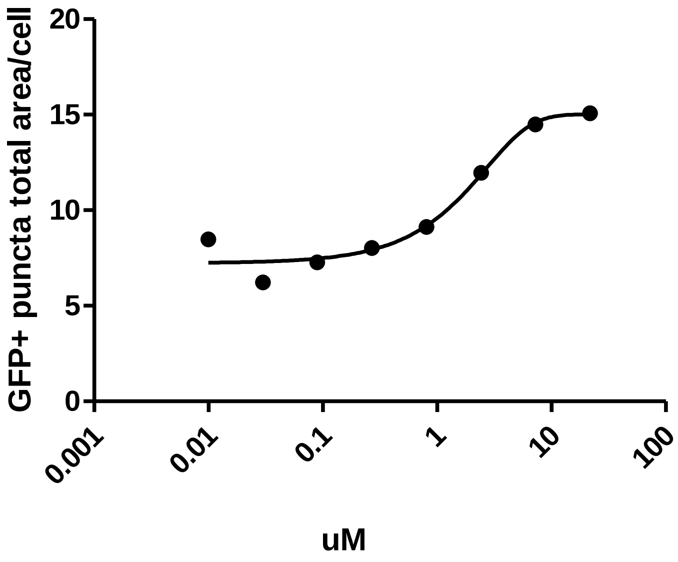

Bepridil

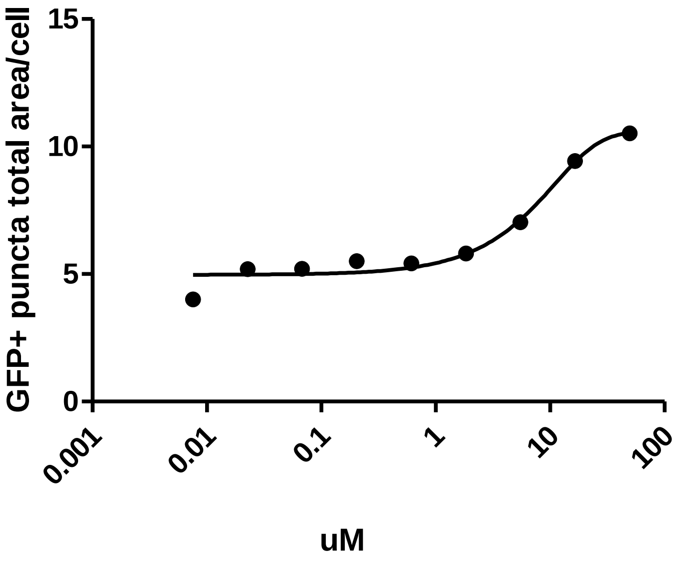

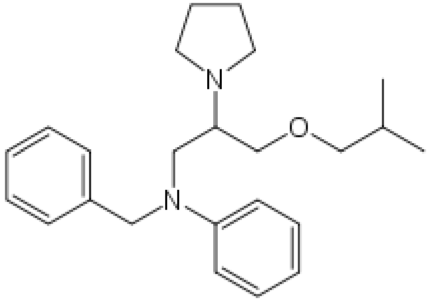

Bepridil

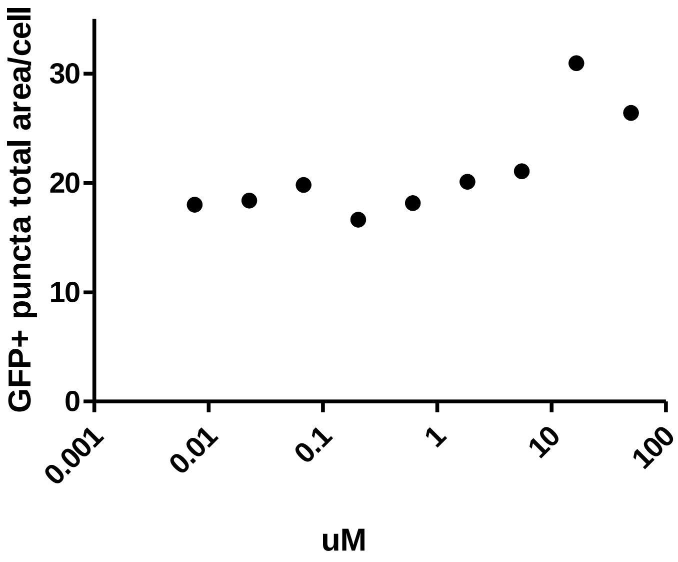

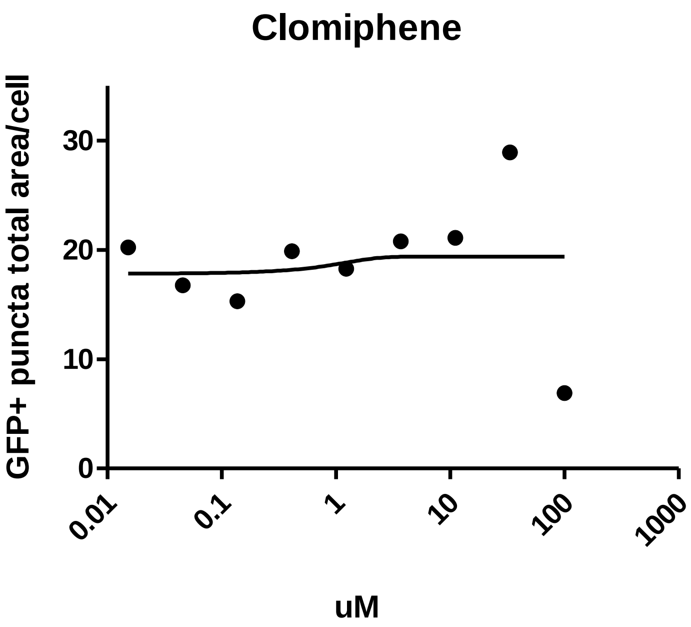
Clomiphene
GFP+ puncta total area/cell
0
10
20
30
0.01
0.1
1
10
100
1000
uM

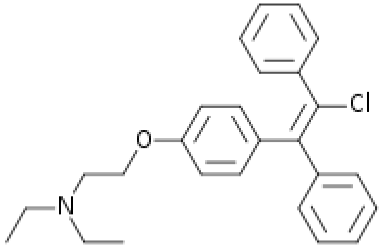
Cl
O
N

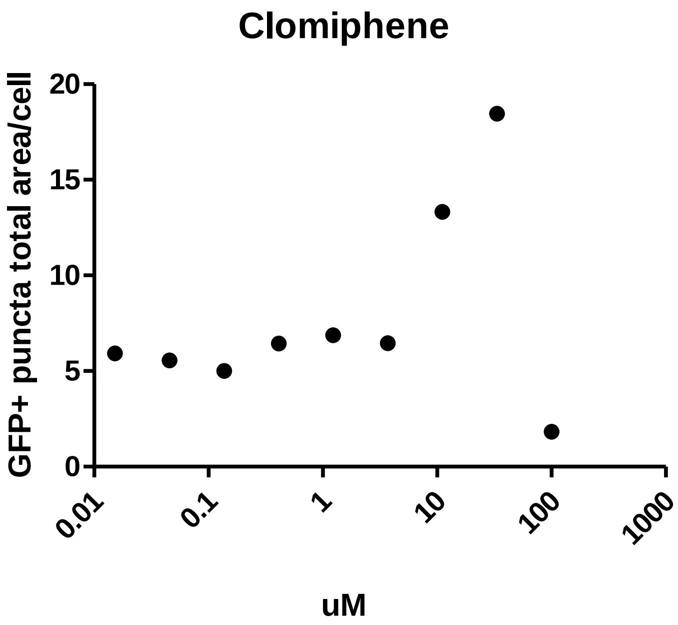
Clomiphene
GFP+ puncta total area/cell
0
5
10
15
20
0.01
0.1
1
10
100
1000
uM

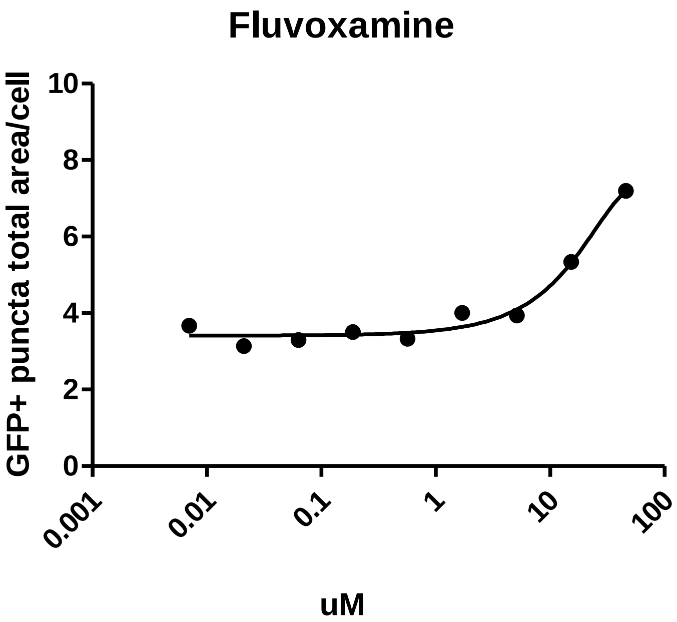
Fluvoxamine
GFP+ puncta total area/cell
0
2
4
6
8
10
0.001
0.01
0.1
1
10
100
uM

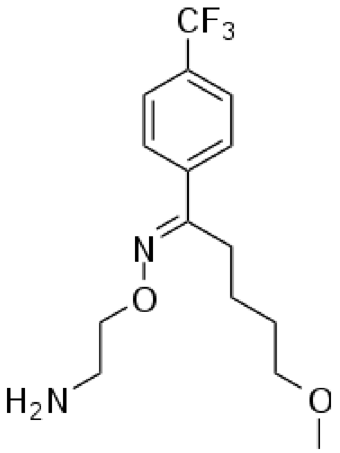
CF3
N
O
H2N
O

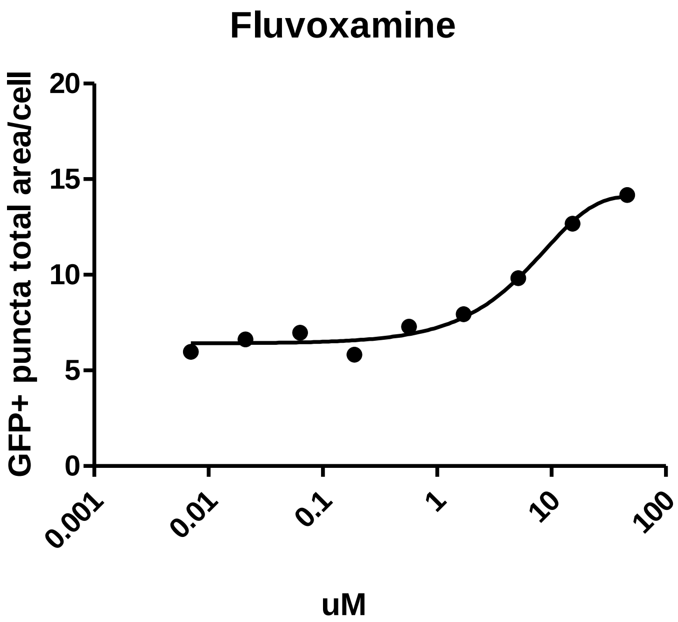
Fluvoxamine
GFP+ puncta total area/cell
0
5
10
15
20
0.001
0.01
0.1
1
10
100
uM

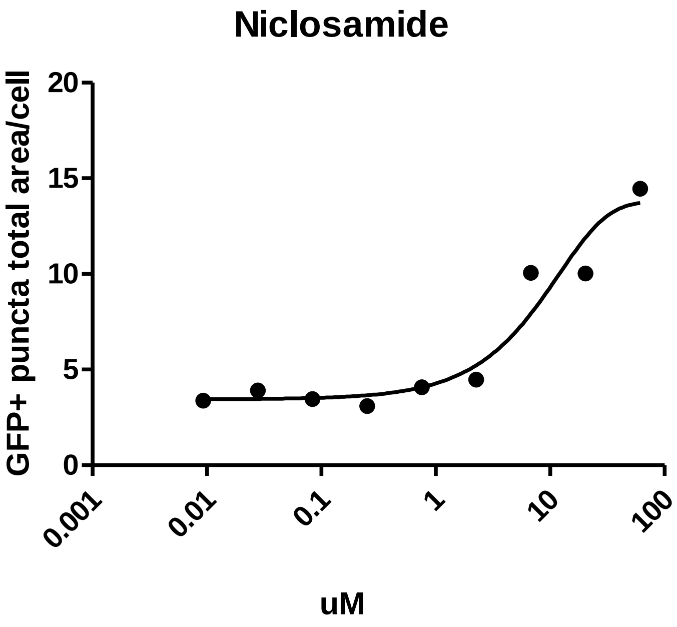
Niclosamide
GFP+ puncta total area/cell
0
5
10
15
20
0.001
0.01
0.1
1
10
100
uM

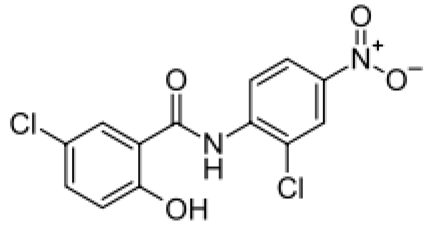
Cl
O
N
H
OH
Cl
O
N+
O−

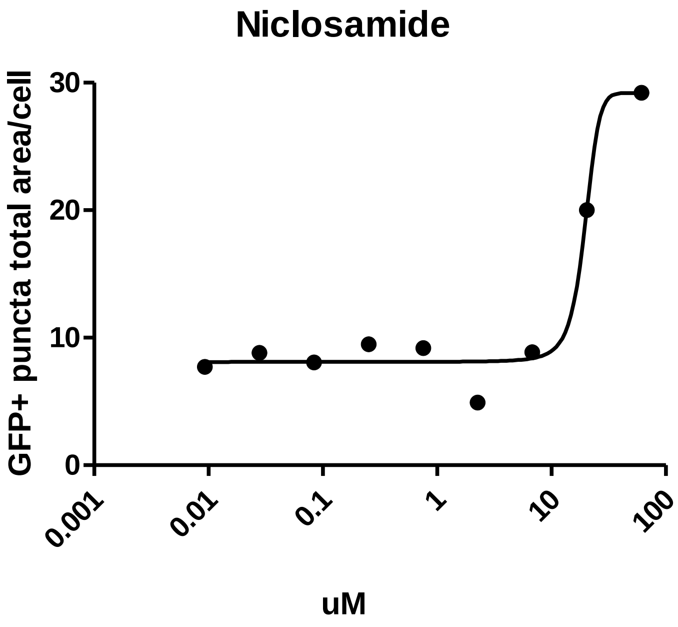
Niclosamide
GFP+ puncta total area/cell
0
10
20
30
0.001
0.01
0.1
1
10
100
uM

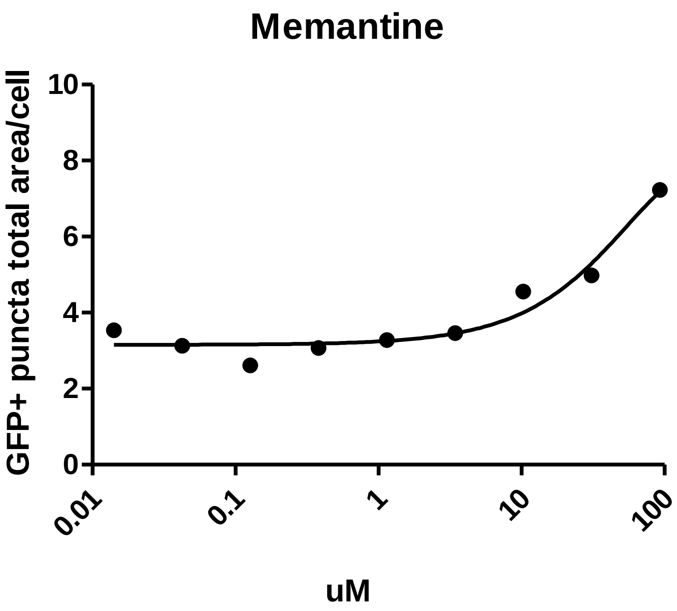
Memantine
GFP+ puncta total area/cell
0
2
4
6
8
10
0.01
0.1
1
10
100
uM

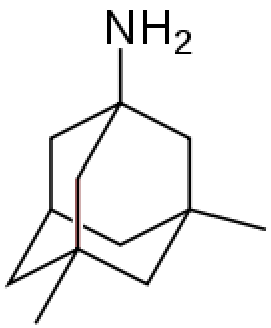
NH2

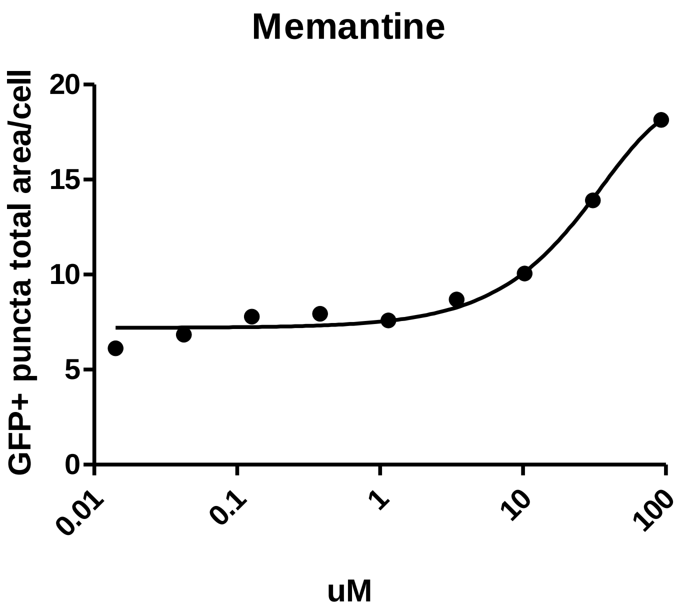
Memantine
GFP+ puncta total area/cell
0
5
10
15
20
0.01
0.1
1
10
100
uM

Nortriptyline

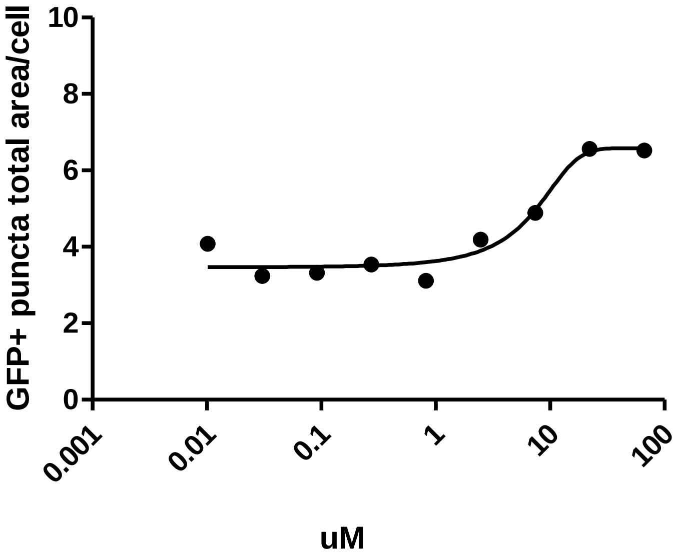

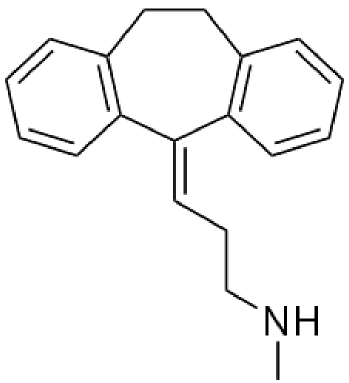

Nortriptyline

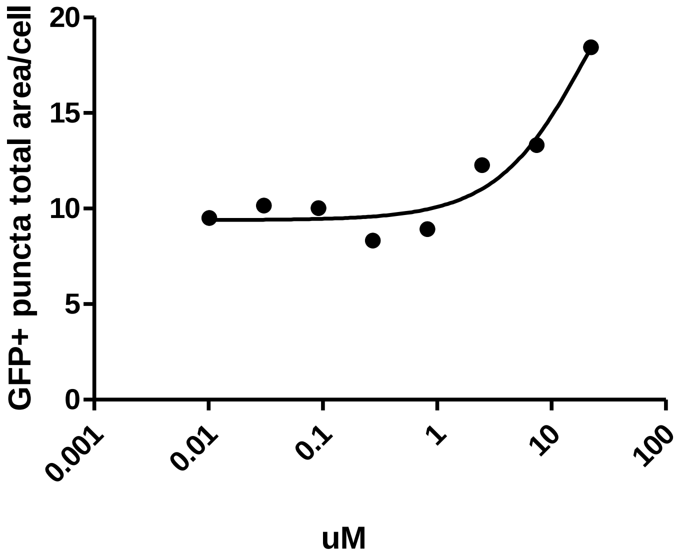

Ticlopidine

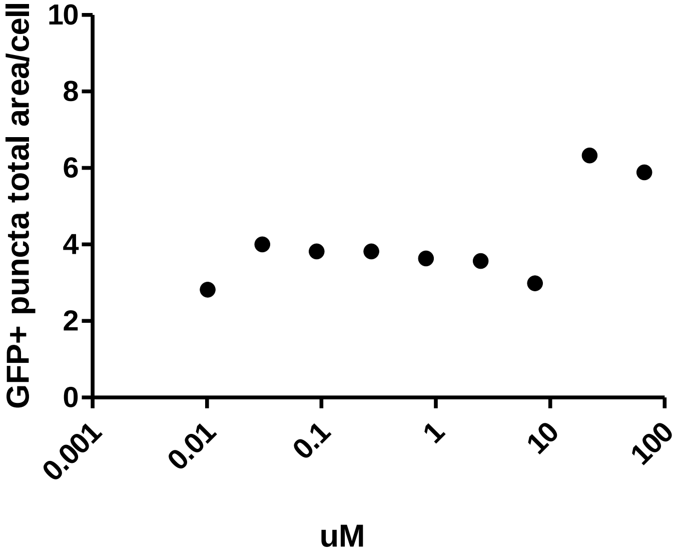

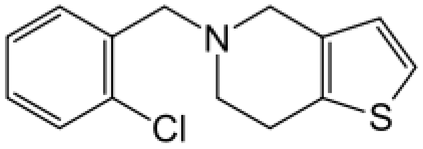

Ticlopidine

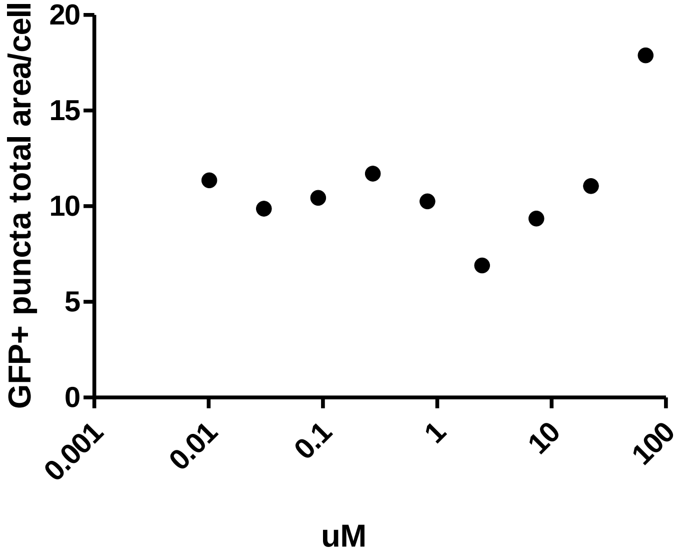

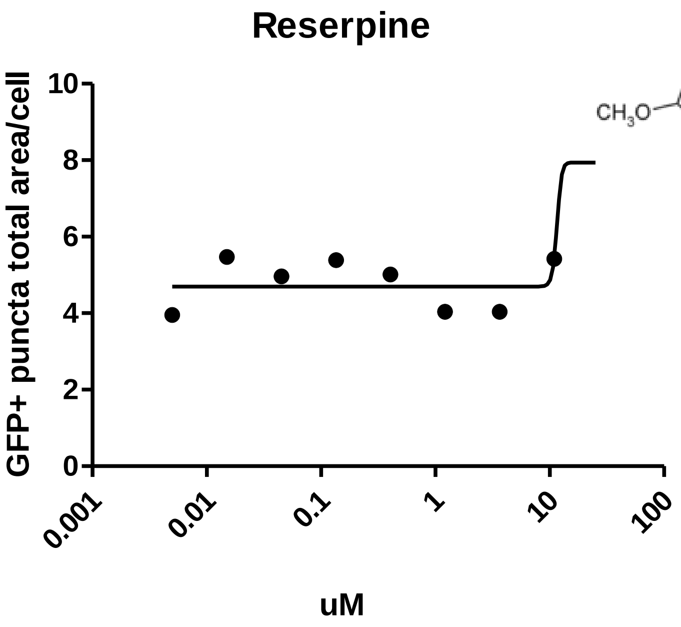
Reserpine
GFP+ puncta total area/cell
0
2
4
6
8
10
0.001
0.01
0.1
1
10
100
uM

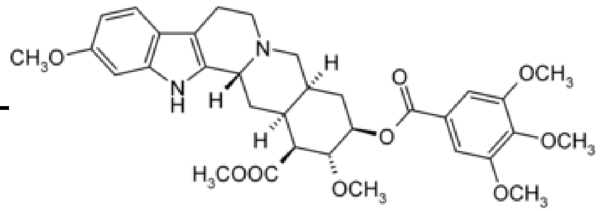
CH3O
N
H
H
H
H
H
O
O
OCH3
OCH3
OCH3
H3COOC
OCH3

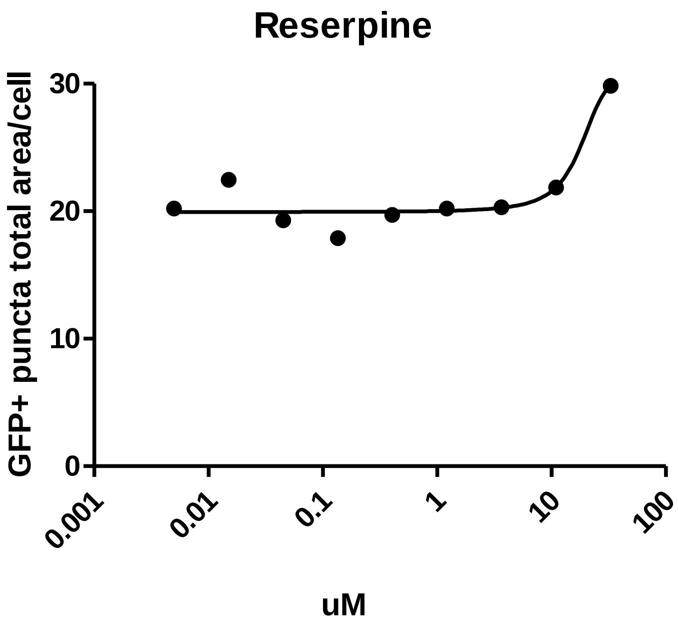
Reserpine
GFP+ puncta total area/cell
0
10
20
30
0.001
0.01
0.1
1
10
100
uM

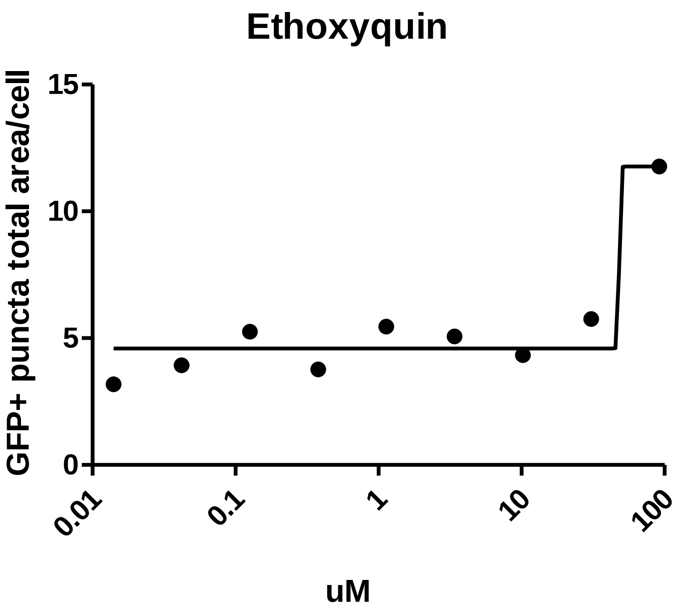
Ethoxyquin
GFP+ puncta total area/cell
0
5
10
15
0.01
0.1
1
10
100
uM

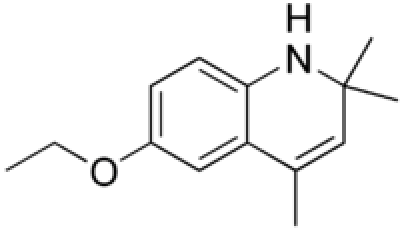
H
N
O

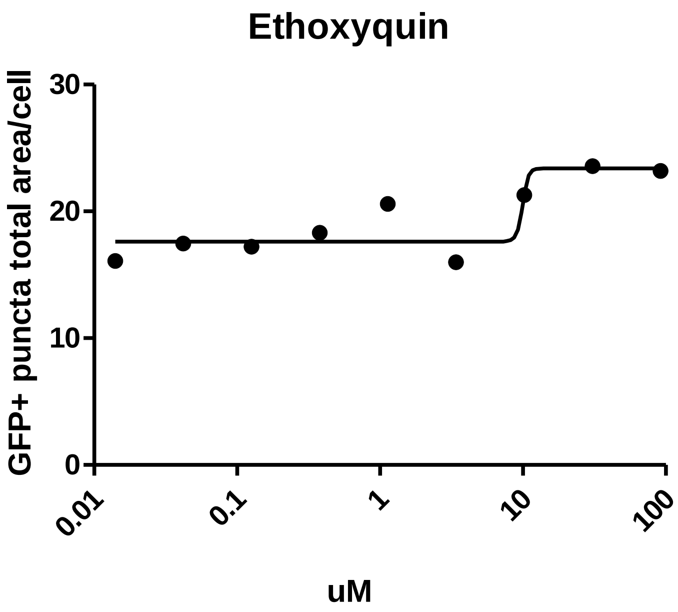
Ethoxyquin
GFP+ puncta total area/cell
0
10
20
30
0.01
0.1
1
10
100
uM

GBR 12909

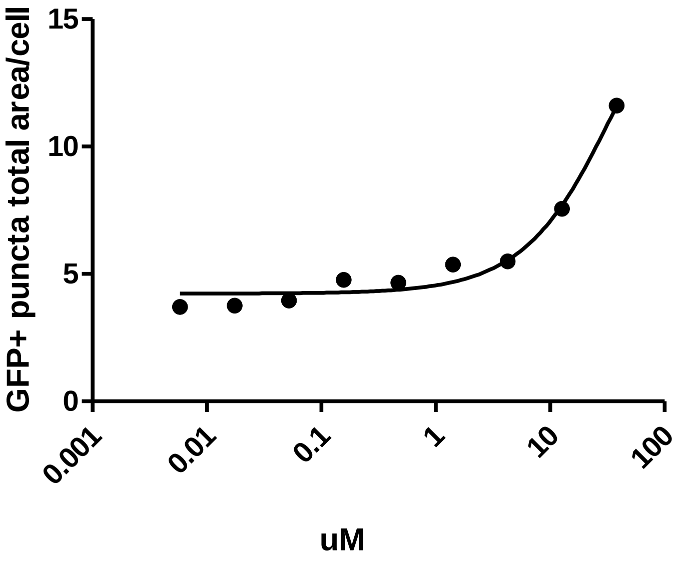

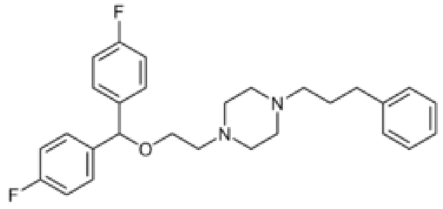

GBR 12909

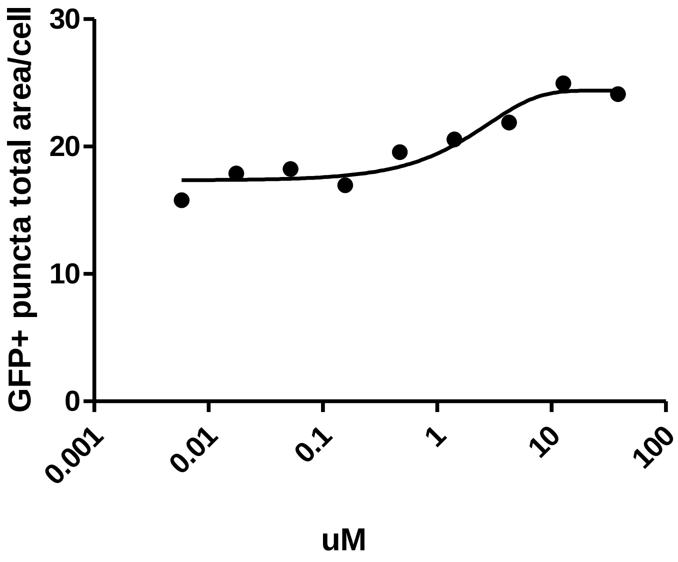

Propafenone

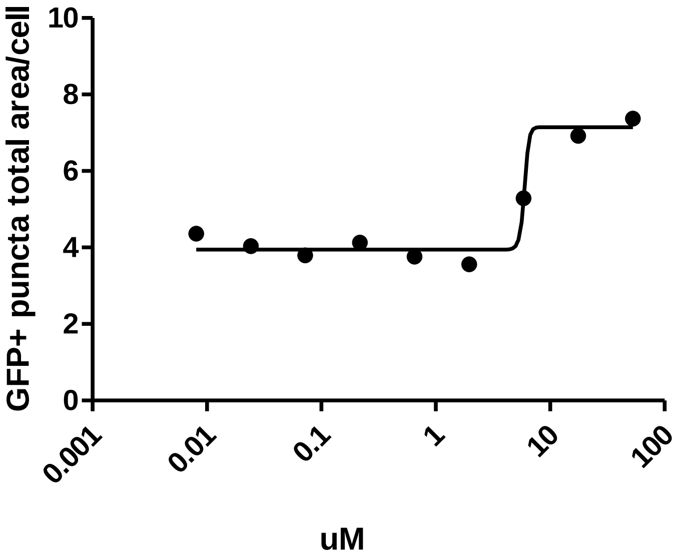

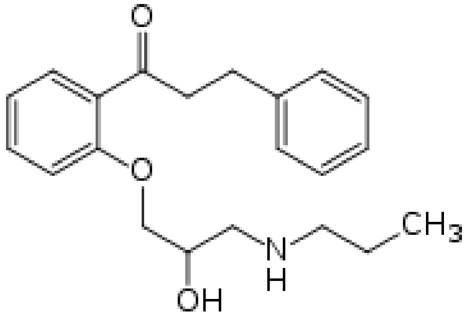

Propafenone

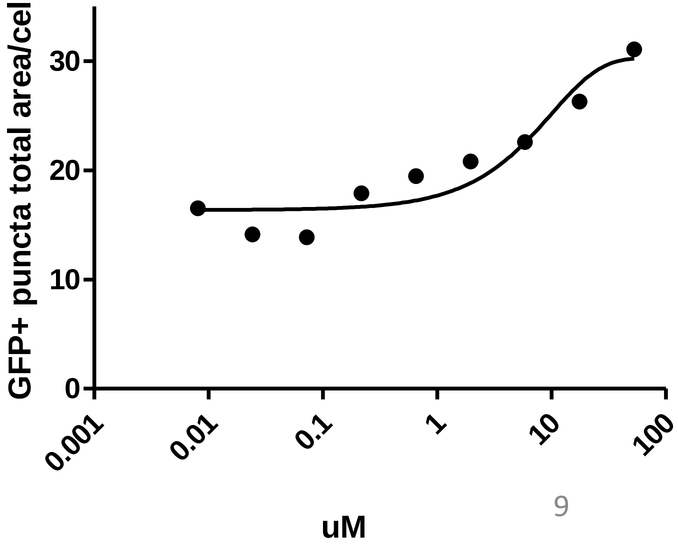

9

Hexachlorophene

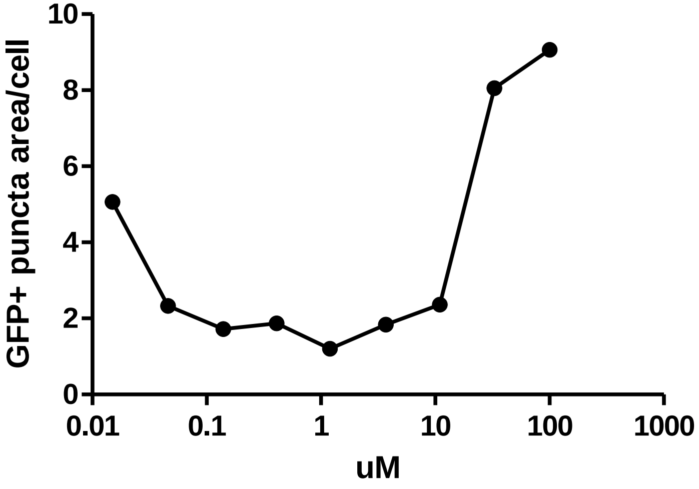

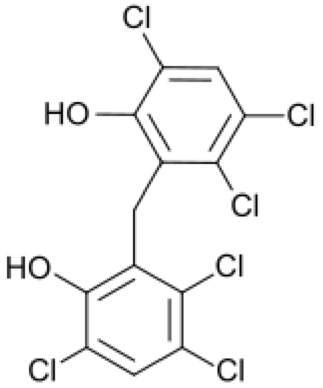

Hexachlorophene

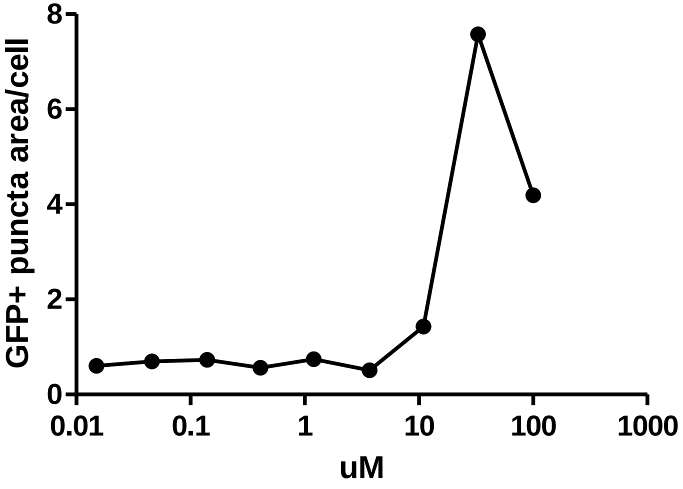

Diperodon

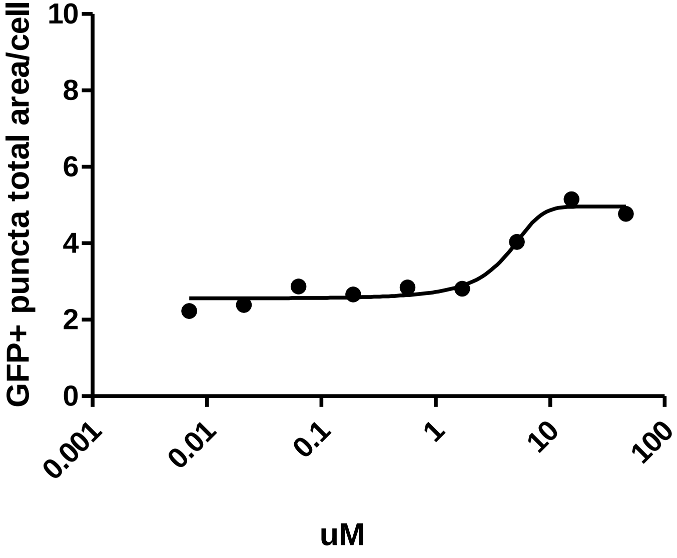

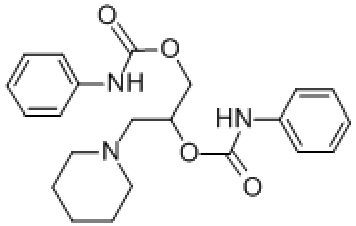

Diperodon

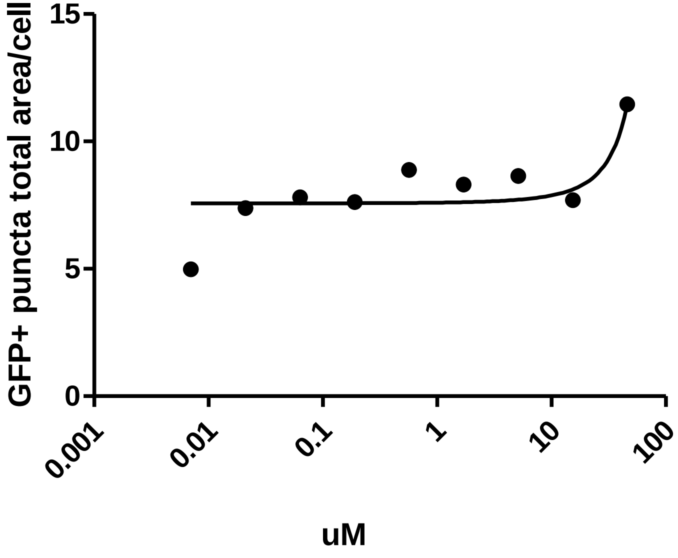

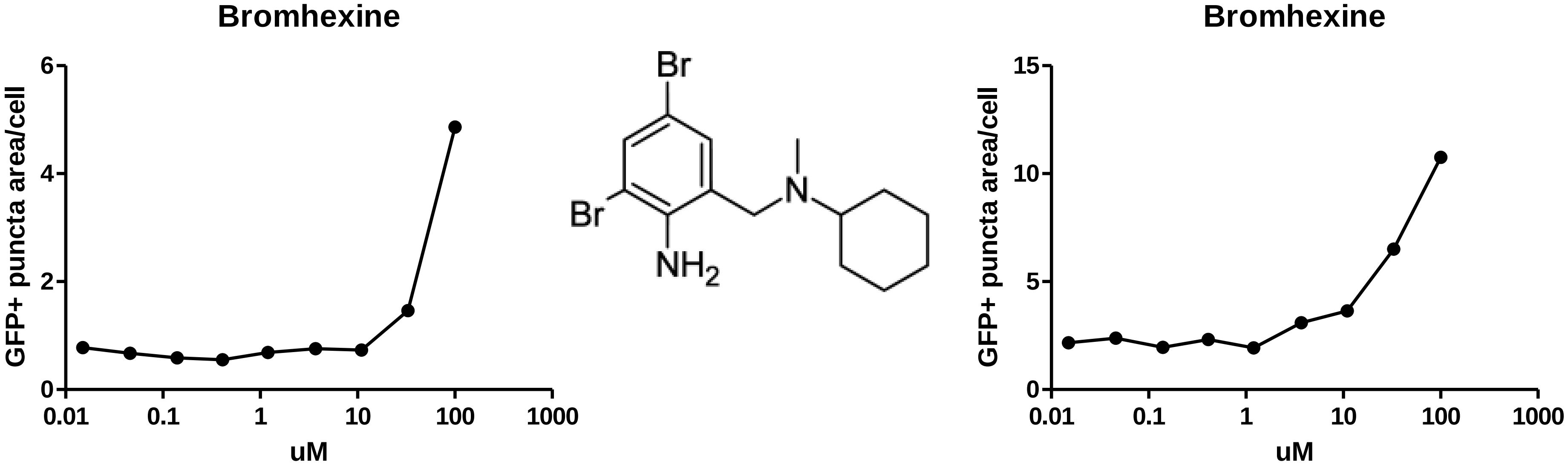
Bromhexine
GFP+ puncta area/cell
0
2
4
6
0.01
0.1
1
10
100
1000
uM
Br
Br
NH2
N
Bromhexine
GFP+ puncta area/cell
0
5
10
15
0.01
0.1
1
10
100
1000
uM

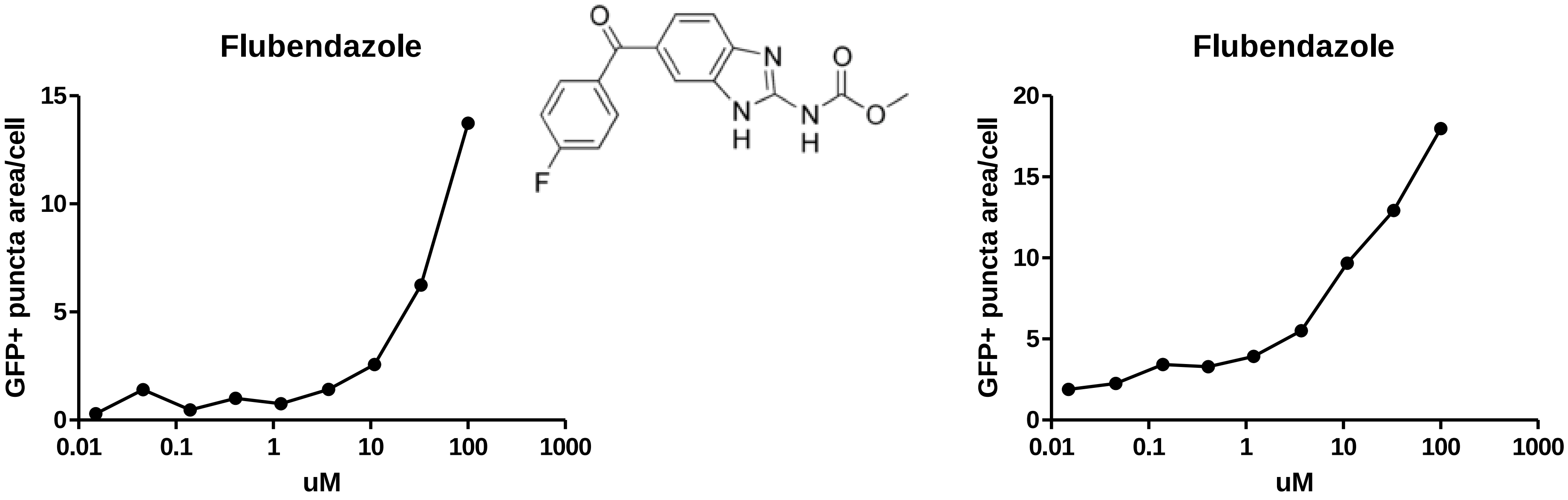
Flubendazole
GFP+ puncta area/cell
0
5
10
15
0.01
0.1
1
10
100
1000
uM
O
N
O
N
H
N
H
O
F
Flubendazole
GFP+ puncta area/cell
0
5
10
15
20
0.01
0.1
1
10
100
1000
uM

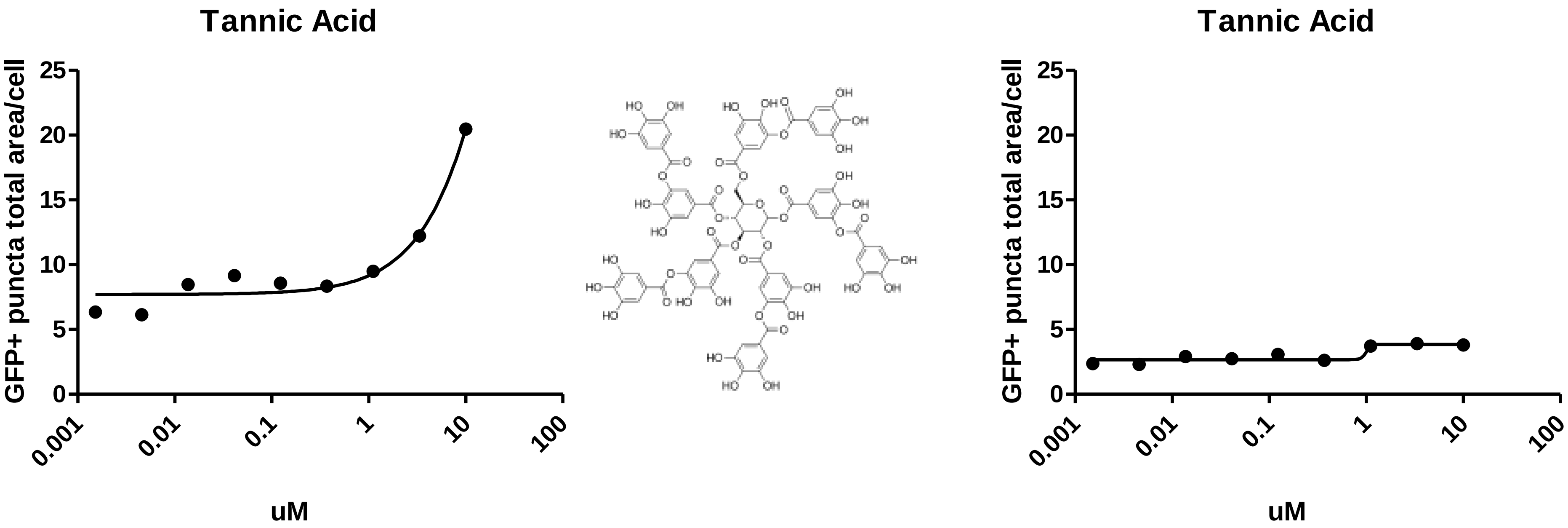
Tannic Acid
GFP+ puncta total area/cell
25
20
15
10
5
0
0.001
0.01
0.1
1
10
100
uM
Tannic Acid
GFP+ puncta total area/cell
25
20
15
10
5
0
0.001
0.01
0.1
1
10
100
uM

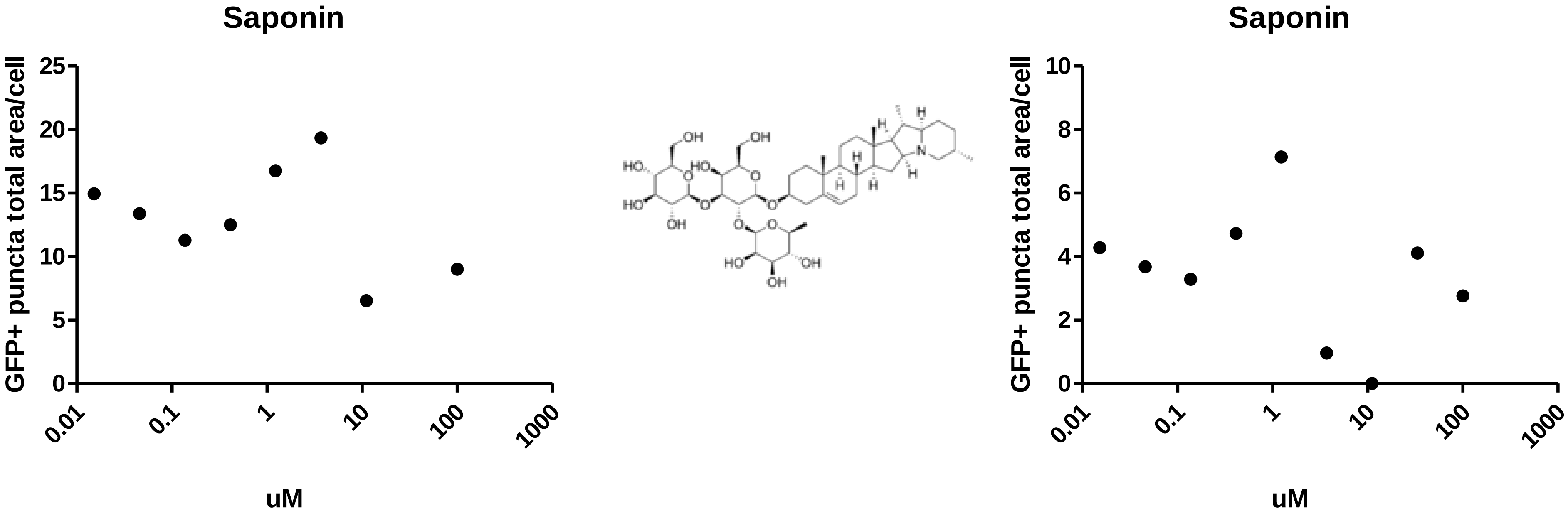
Saponin
GFP+ puncta total area/cell
25
20
15
10
5
0
0.01
0.1
1
10
100
1000
uM
Saponin
GFP+ puncta total area/cell
10
8
6
4
2
0
0.01
0.1
1
10
100
1000
uM

Vinblastine sulfate

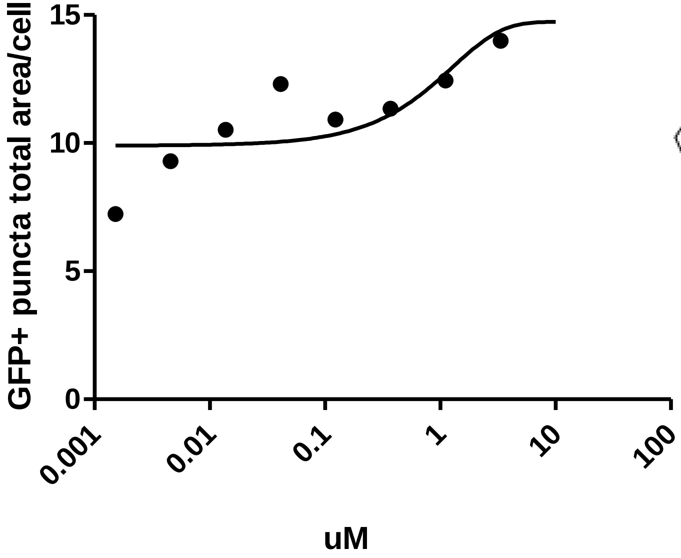

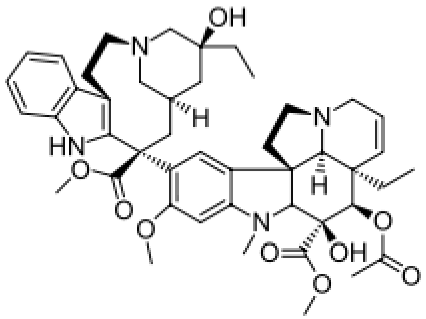

Vinblastine sulfate

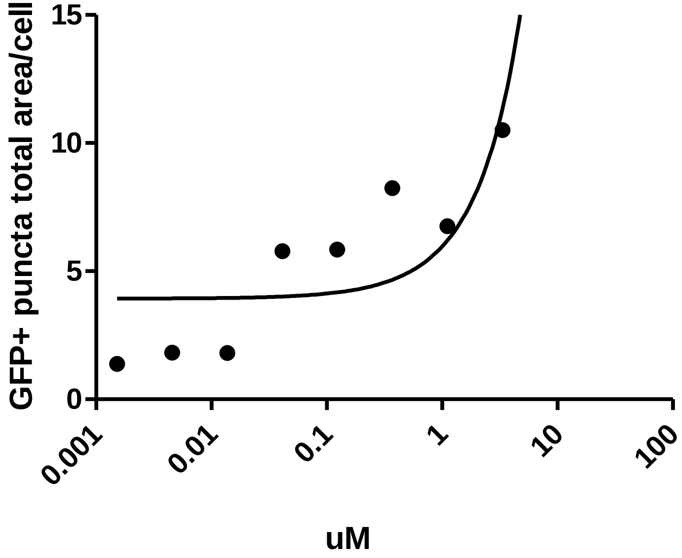

Phenylmercuric acetate

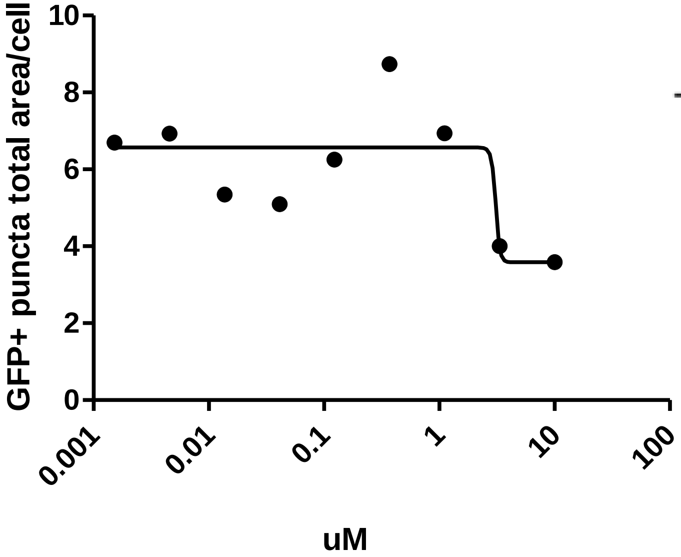

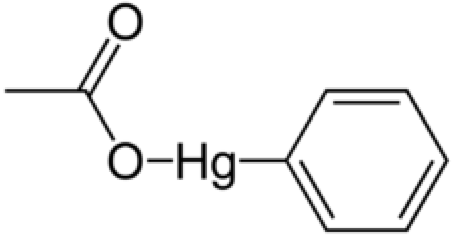

Phenylmercuric acetate

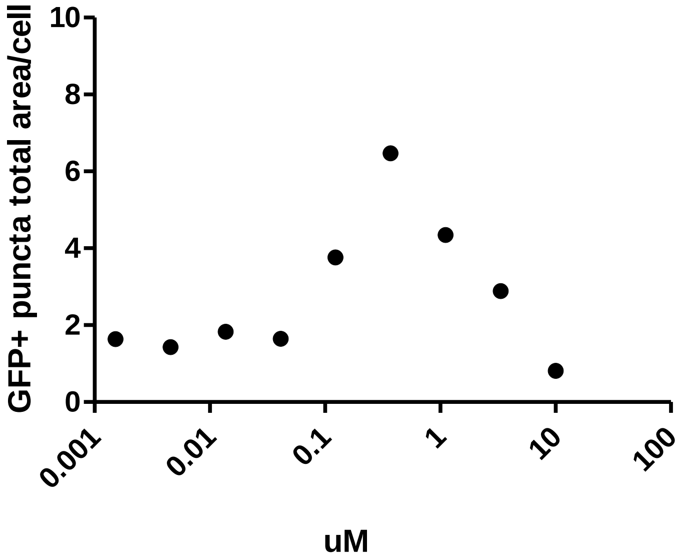

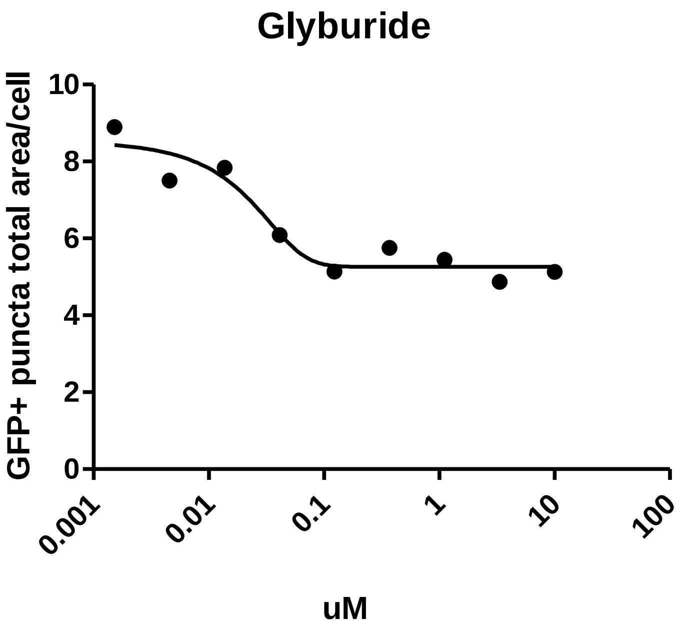
Glyburide
GFP+ puncta total area/cell
10
8
6
4
2
0
0.001
0.01
0.1
1
10
100
uM

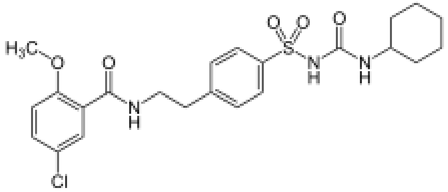
H3C
O
O
N
H
O O
S
N
H
O
N
H
Cl

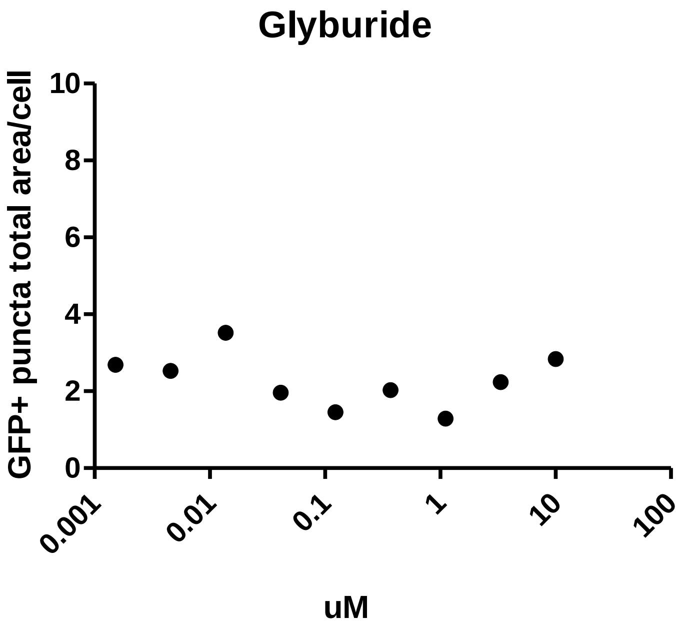
Glyburide
GFP+ puncta total area/cell
10
8
6
4
2
0
0.001
0.01
0.1
1
10
100
uM

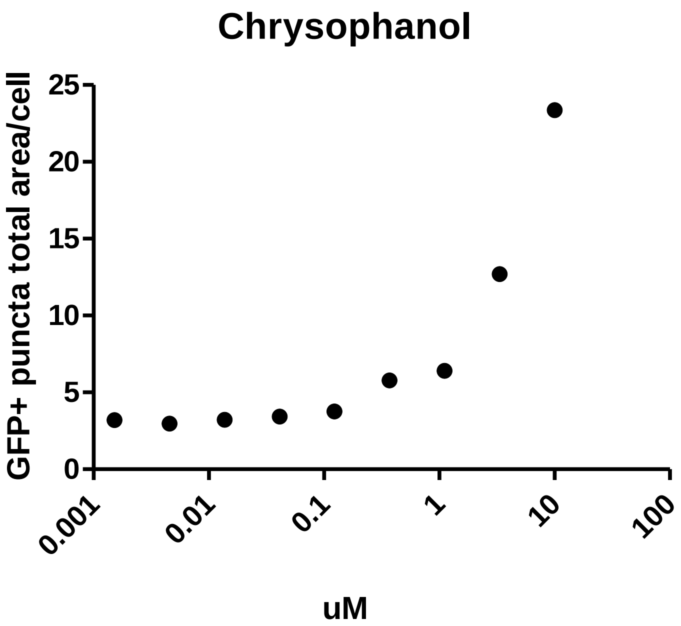
Chrysophanol
GFP+ puncta total area/cell
25
20
15
10
5
0
0.001
0.01
0.1
1
10
100
uM

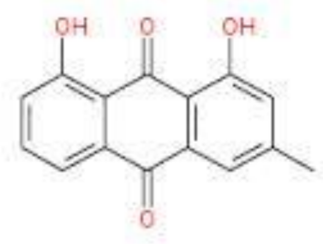
OH
O
OH
O

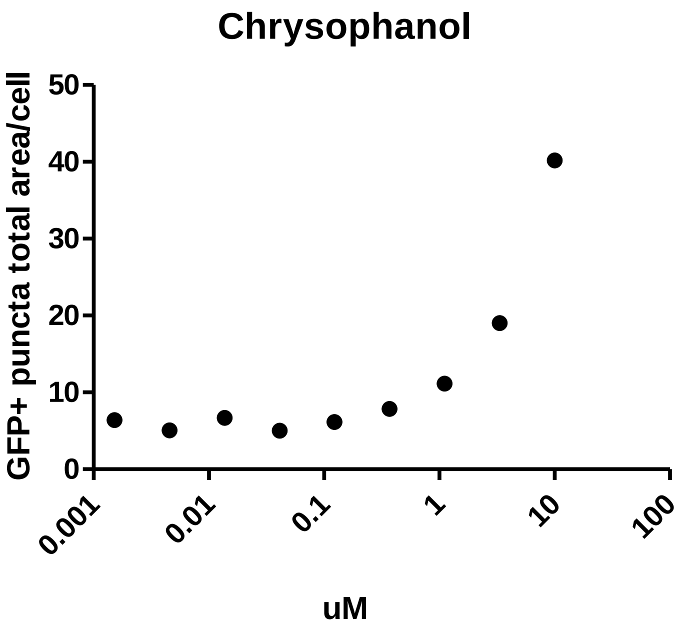
Chrysophanol
GFP+ puncta total area/cell
50
40
30
20
10
0
0.001
0.01
0.1
1
10
100
uM

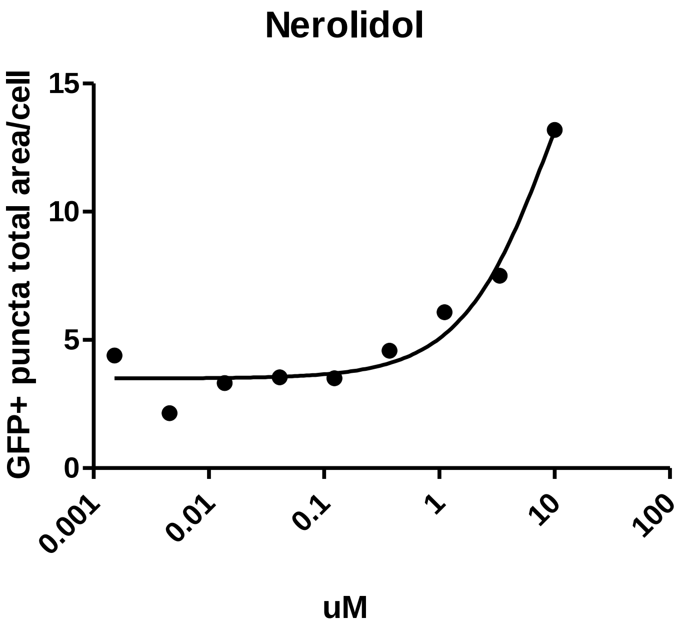
Nerolidol
GFP+ puncta total area/cell
15
10
5
0
0.001
0.01
0.1
1
10
100
uM

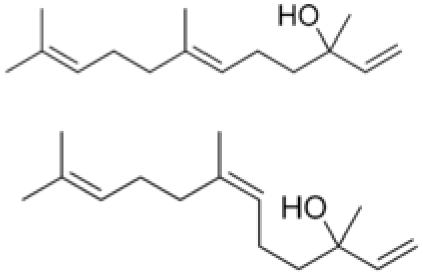
HO
HO

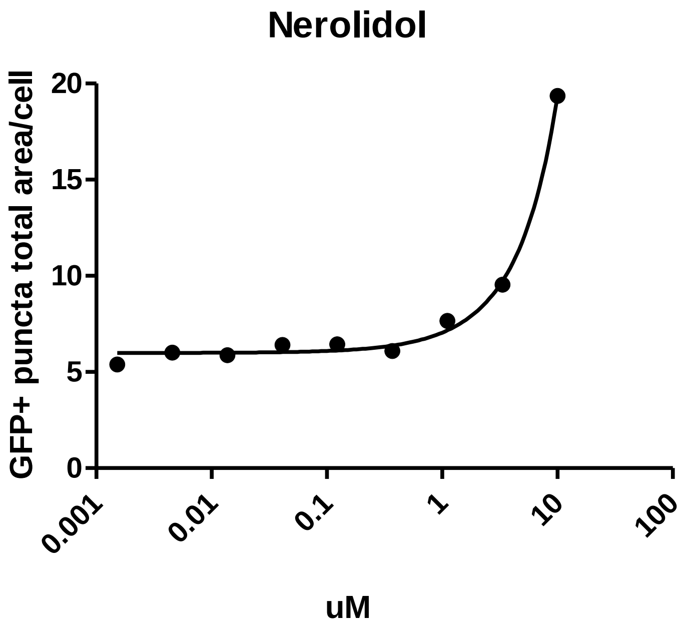
Nerolidol
GFP+ puncta total area/cell
20
15
10
5
0
0.001
0.01
0.1
1
10
100
uM

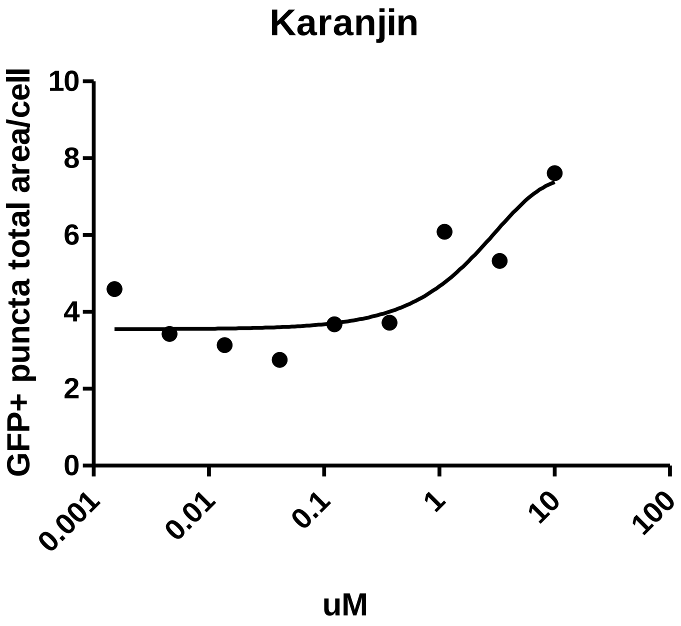
Karanjin
GFP+ puncta total area/cell
10
8
6
4
2
0
0.001
0.01
0.1
1
10
100
uM

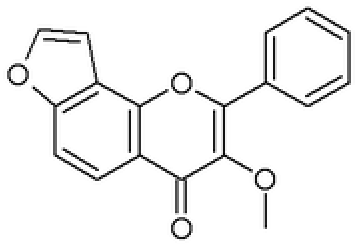
O
O
O
O

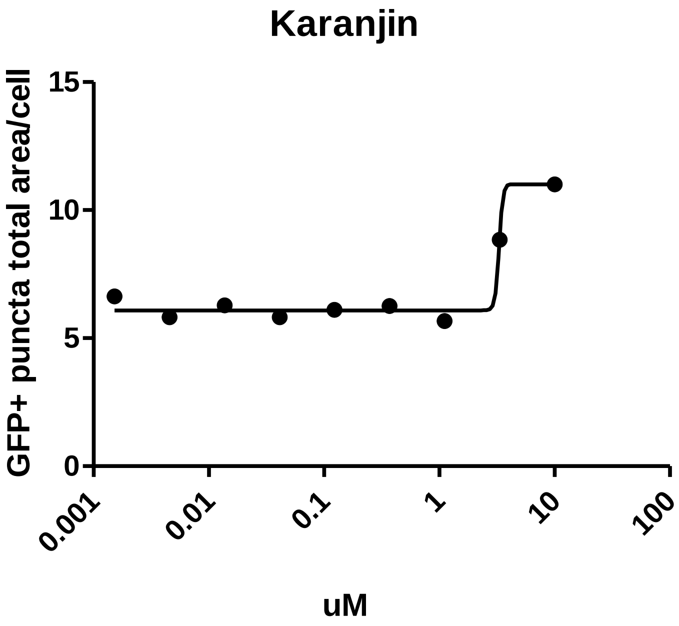
Karanjin
GFP+ puncta total area/cell
15
10
5
0
0.001
0.01
0.1
1
10
100
uM

Berbamine Hydrochloride

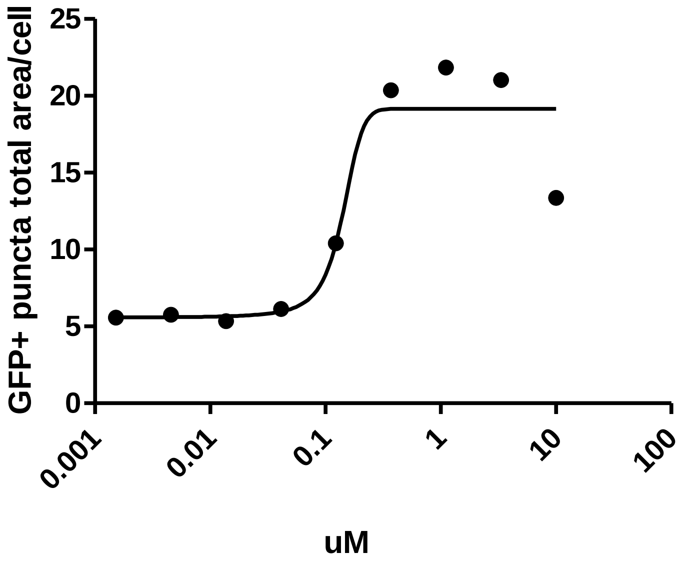

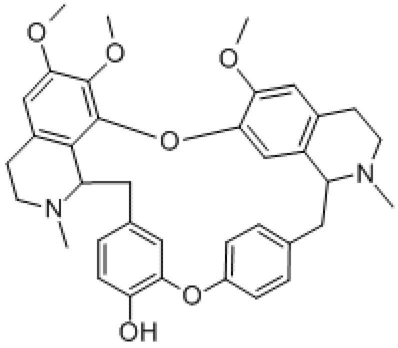

Berbamine Hydrochloride

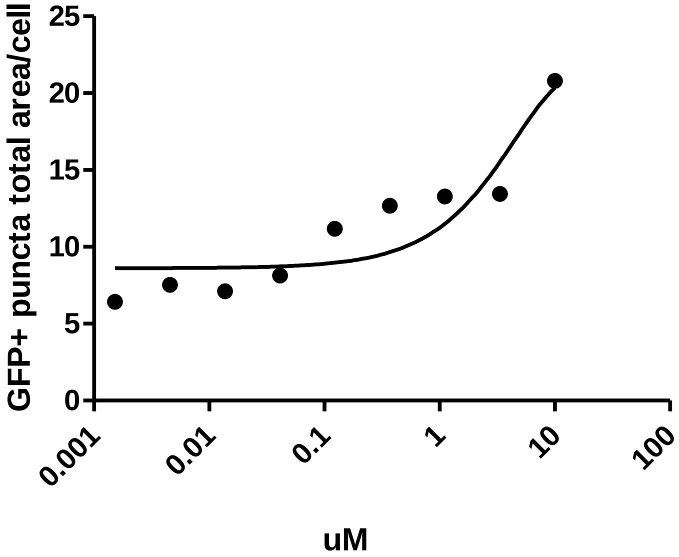

alpha-Mangostin

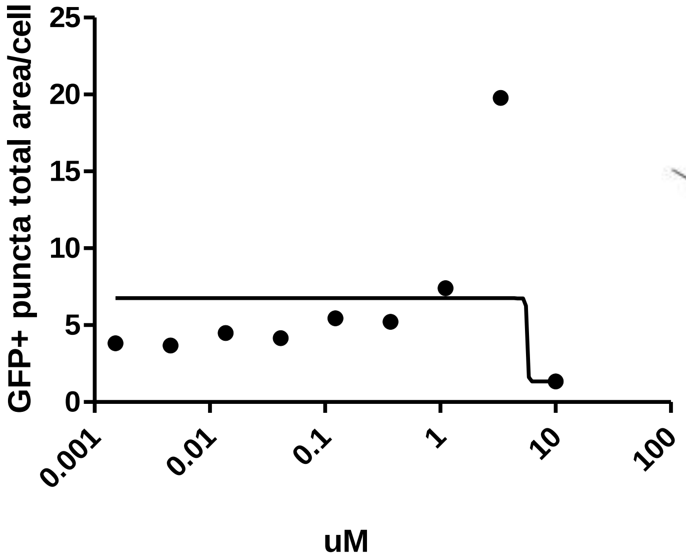

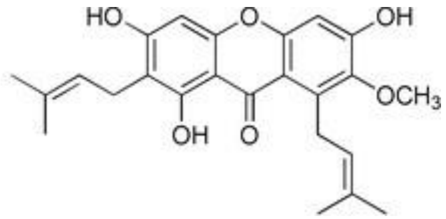

alpha-Mangostin

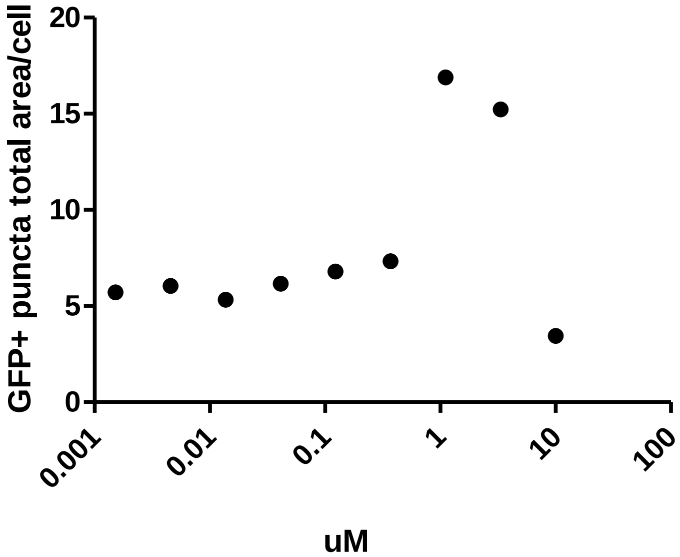

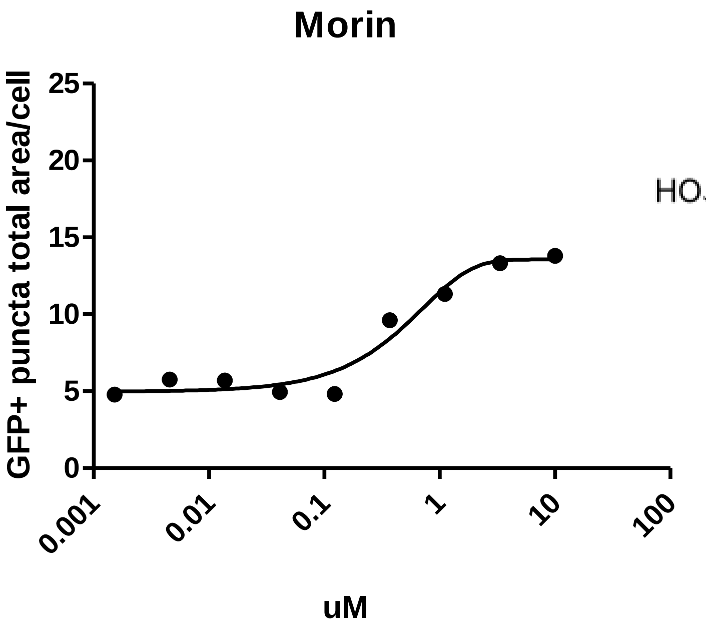
Morin
GFP+ puncta total area/cell
25
20
15
10
5
0
0.001
0.01
0.1
1
10
100
uM

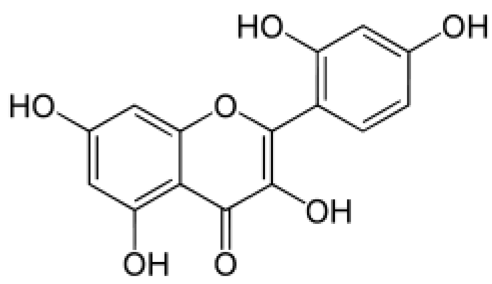
HO
OH
HO
O
OH
OH
O

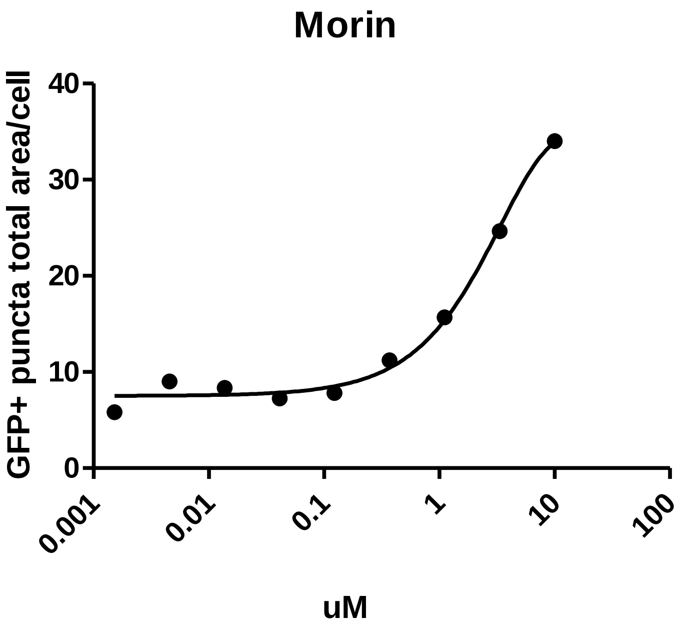
Morin
GFP+ puncta total area/cell
40
30
20
10
0
0.001
0.01
0.1
1
10
100
uM

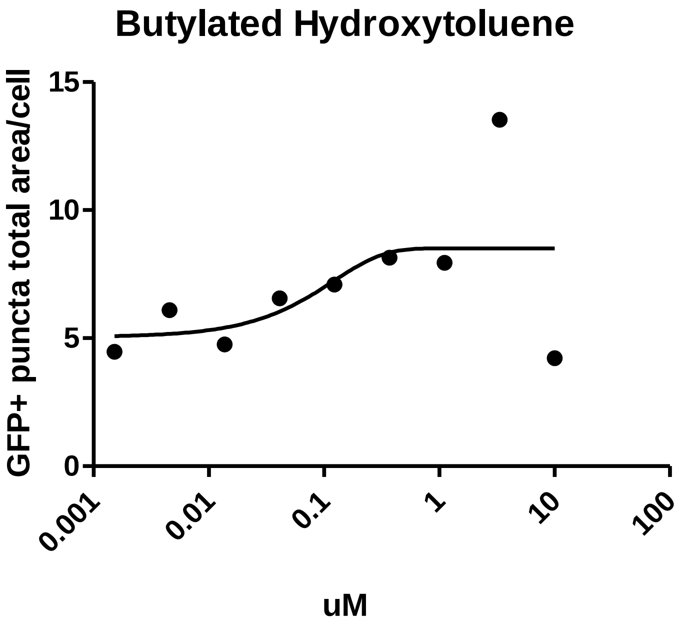
Butylated Hydroxytoluene
GFP+ puncta total area/cell
15
10
5
0
0.001
0.01
0.1
1
10
100
uM

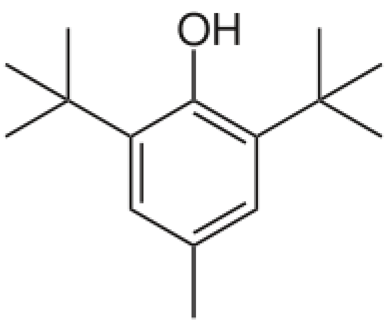
OH

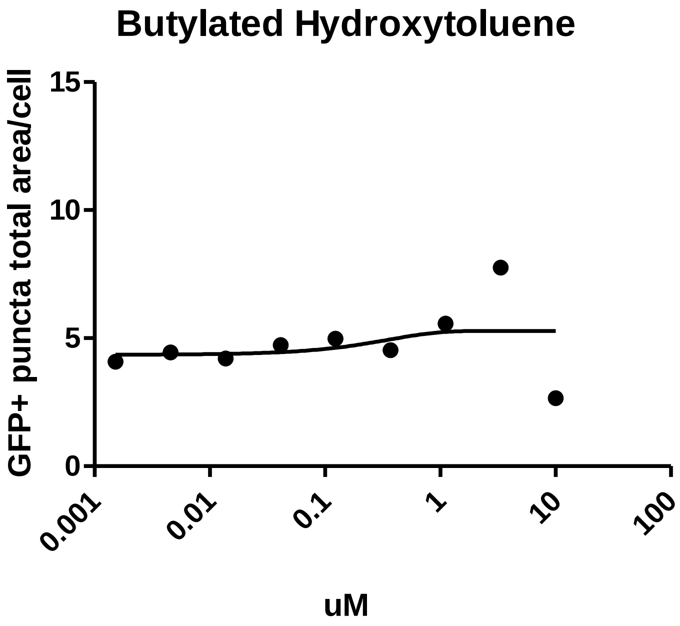
Butylated Hydroxytoluene
GFP+ puncta total area/cell
15
10
5
0
0.001
0.01
0.1
1
10
100
uM

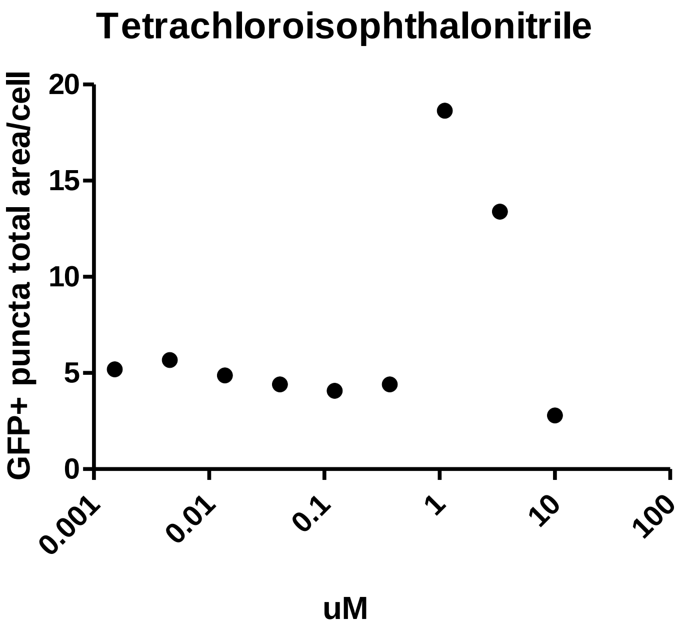
Tetrachloroisophthalonitrile
GFP+ puncta total area/cell
20
15
10
5
0
0.001
0.01
0.1
1
10
100
uM

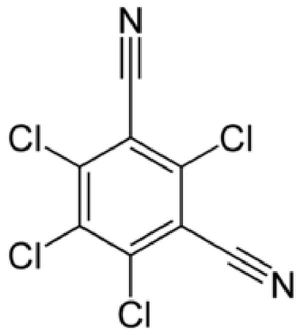
N
Cl
Cl
Cl
N
Cl

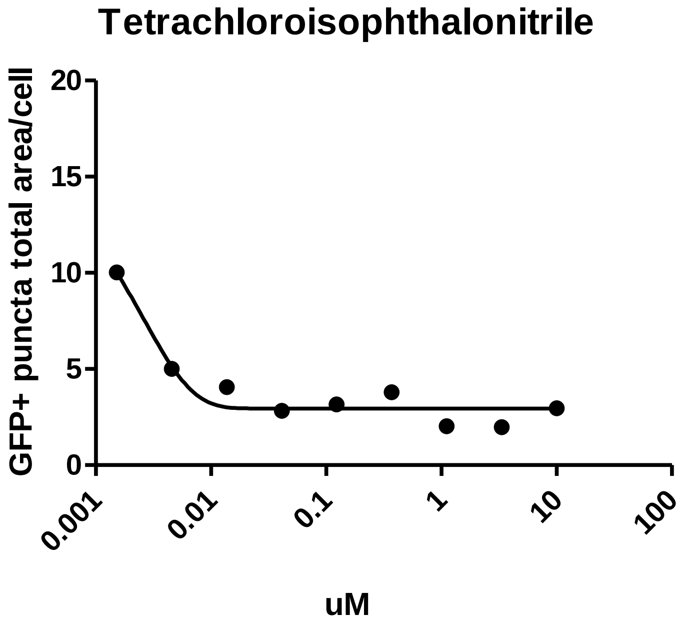
Tetrachloroisophthalonitrile
GFP+ puncta total area/cell
20
15
10
5
0
0.001
0.01
0.1
1
10
100
uM

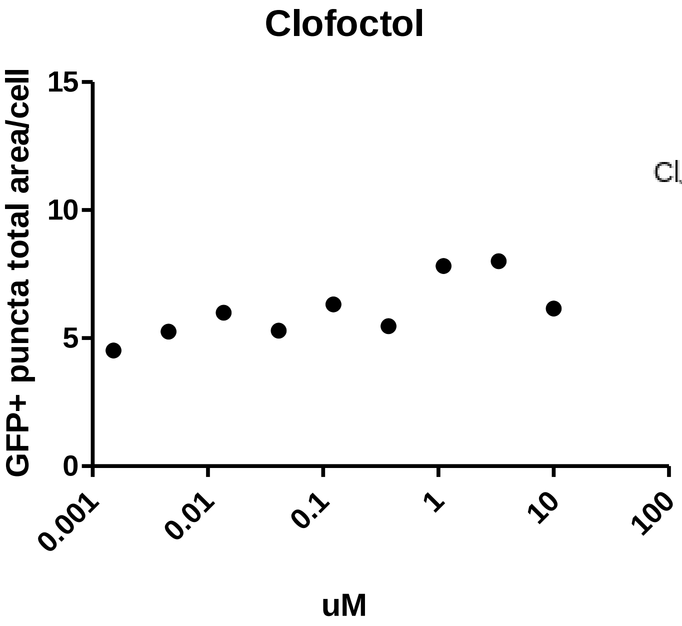
Clofoctol
GFP+ puncta total area/cell
15
10
5
0
0.001
0.01
0.1
1
10
100
uM

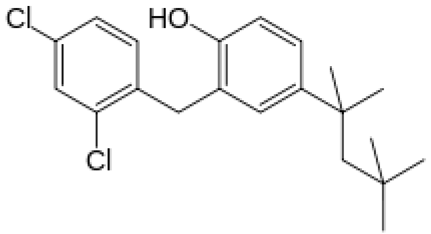
Cl
HO
Cl

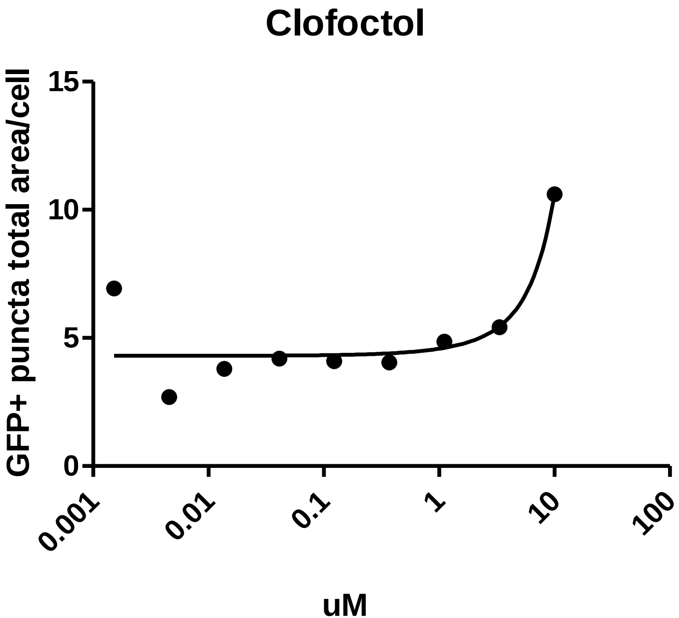
Clofoctol
GFP+ puncta total area/cell
15
10
5
0
0.001
0.01
0.1
1
10
100
uM

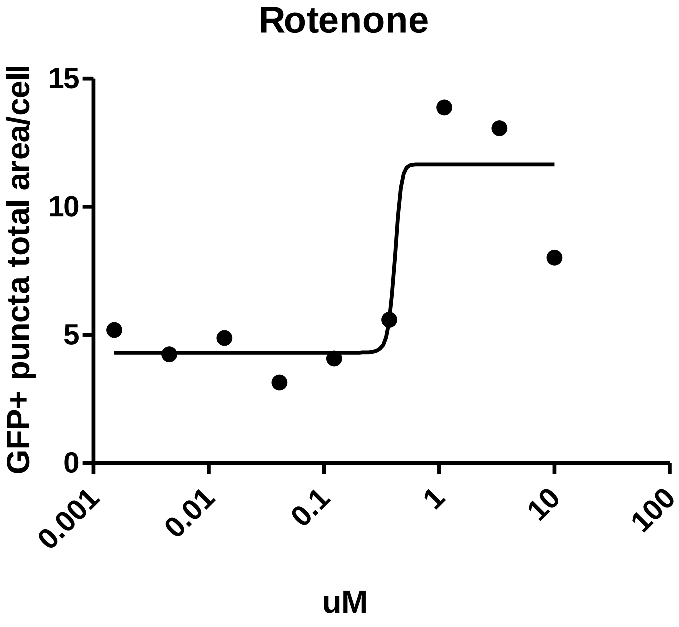
Rotenone
GFP+ puncta total area/cell
15
10
5
0
0.001
0.01
0.1
1
10
100
uM

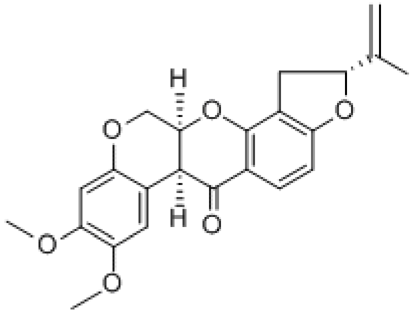
H
O
O
O
O
H
O
O
O

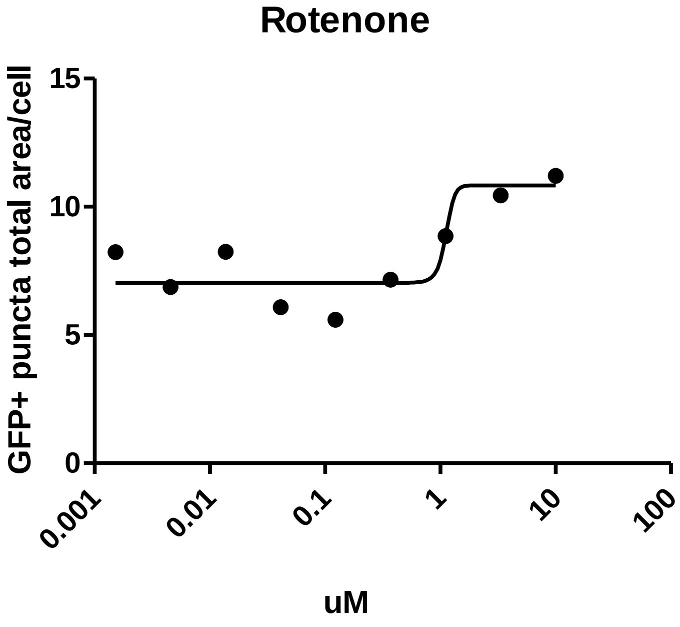
Rotenone
GFP+ puncta total area/cell
15
10
5
0
0.001
0.01
0.1
1
10
100
uM

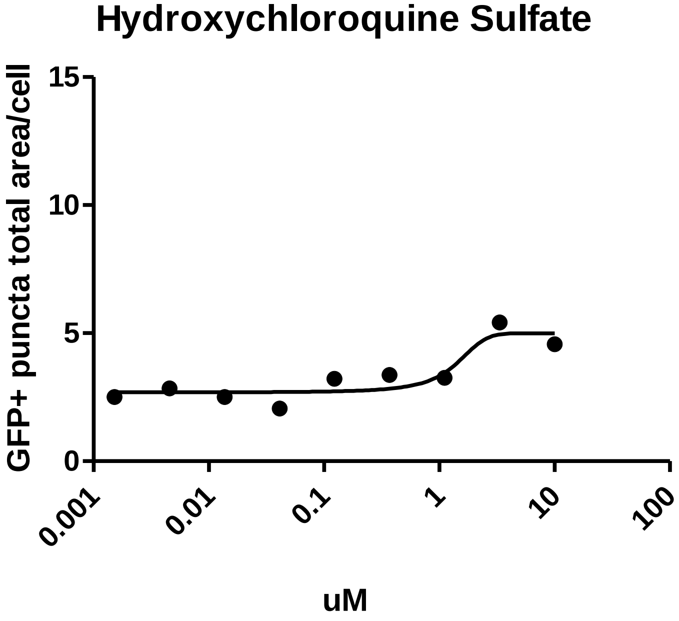
Hydroxychloroquine Sulfate
GFP+ puncta total area/cell
15
10
5
0
0.001
0.01
0.1
1
10
100
uM

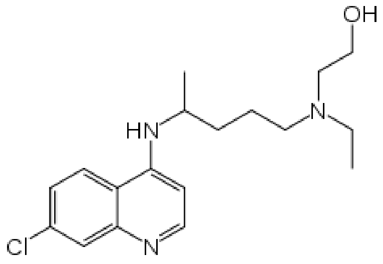
OH
HN
N
Cl
N

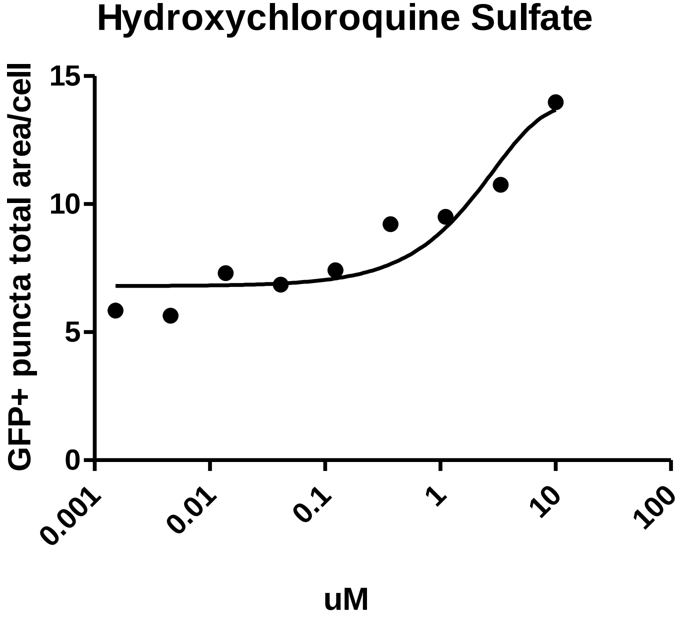
Hydroxychloroquine Sulfate
GFP+ puncta total area/cell
15
10
5
0
0.001
0.01
0.1
1
10
100
uM

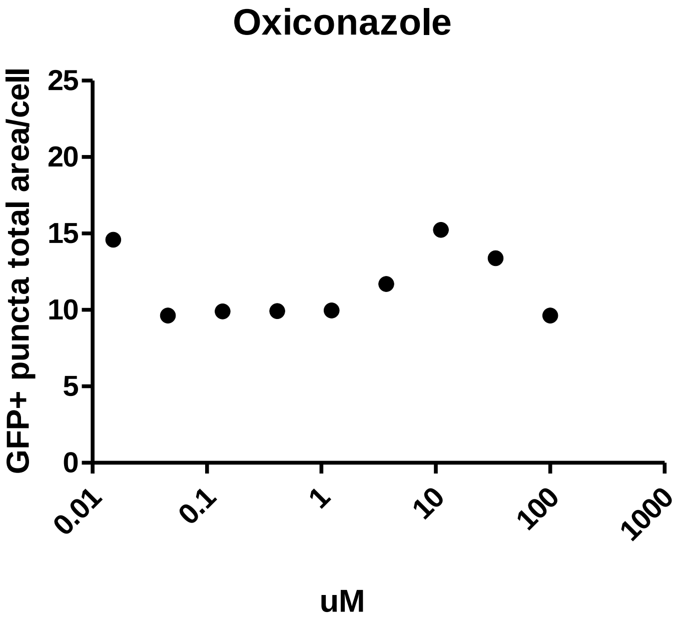
Oxiconazole
GFP+ puncta total area/cell
25
20
15
10
5
0
0.01
0.1
1
10
100
1000
uM

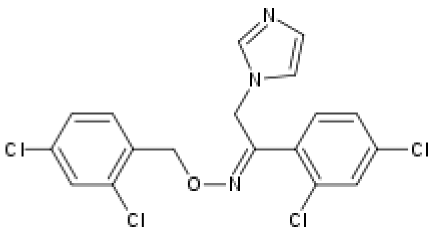
N
N
Cl
O
N
Cl
Cl
Cl

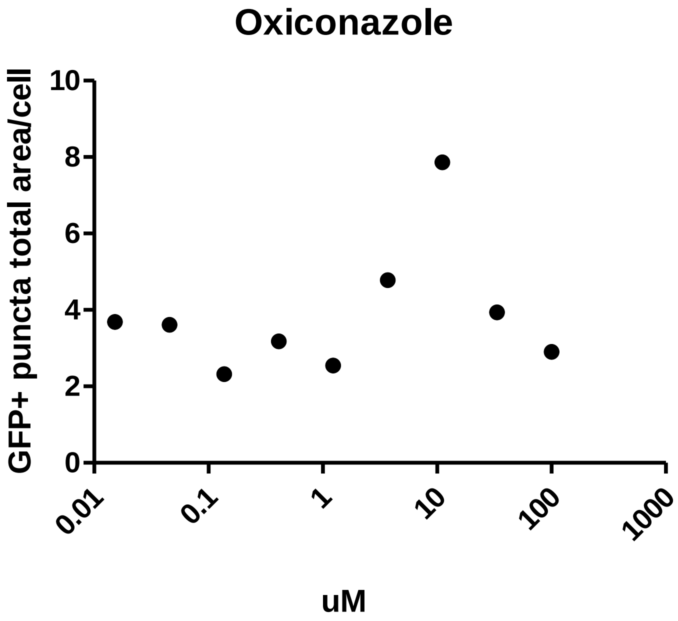
Oxiconazole
GFP+ puncta total area/cell
10
8
6
4
2
0
0.01
0.1
1
10
100
1000
uM

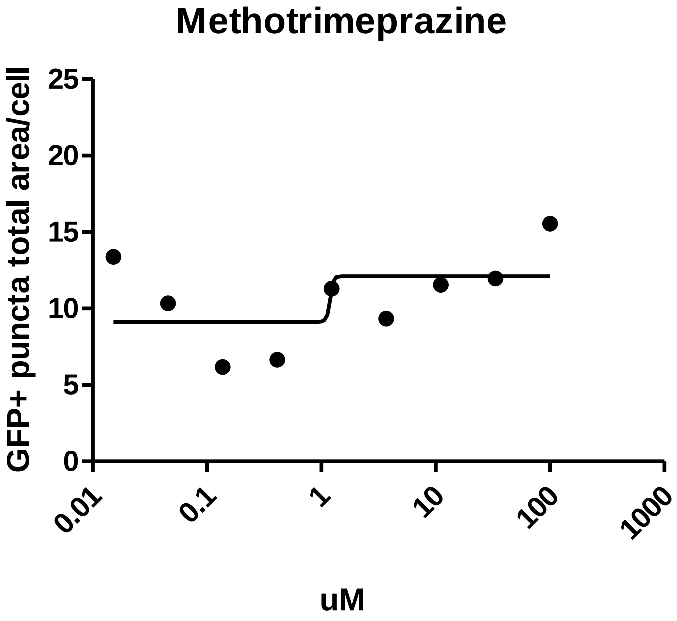
Methotrimeprazine
GFP+ puncta total area/cell
25
20
15
10
5
0
0.01
0.1
1
10
100
1000
uM

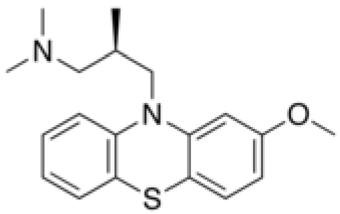
N
N
O
S

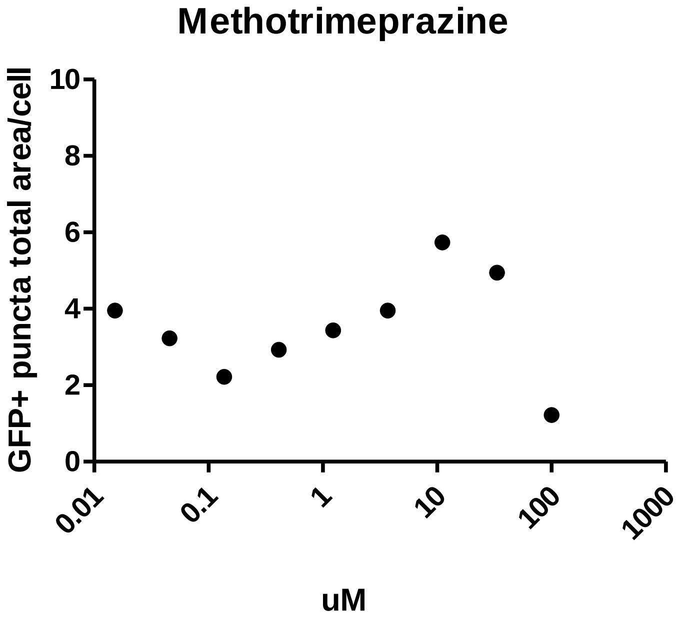
Methotrimeprazine
GFP+ puncta total area/cell
10
8
6
4
2
0
0.01
0.1
1
10
100
1000
uM

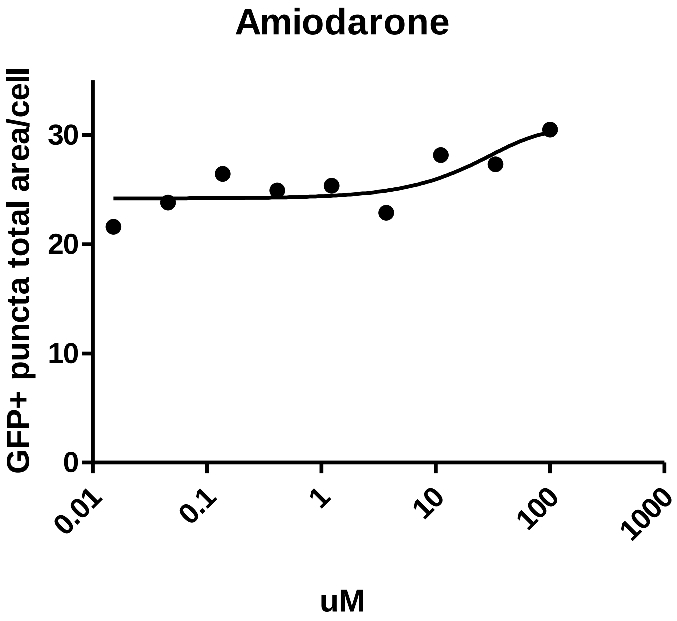
Amiodarone
GFP+ puncta total area/cell
0
10
20
30
0.01
0.1
1
10
100
1000
uM

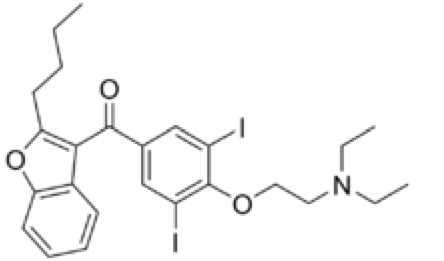
O
O
I
I
O
N

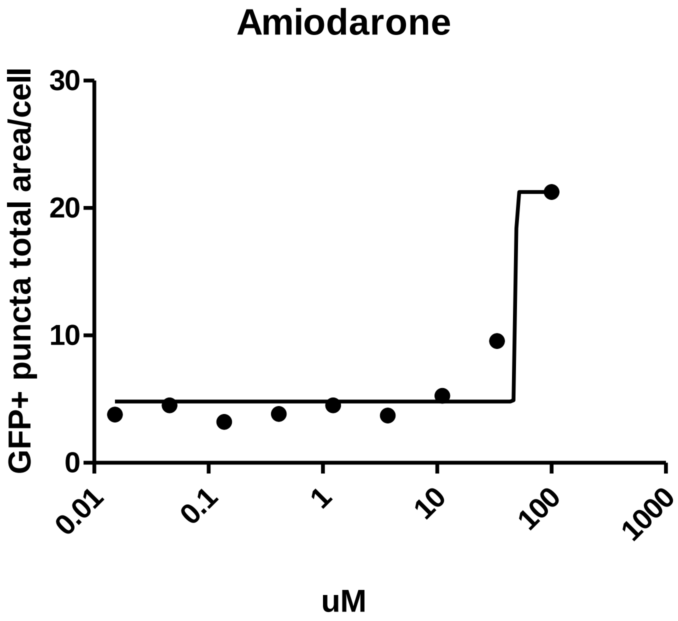
Amiodarone
GFP+ puncta total area/cell
0
10
20
30
0.01
0.1
1
10
100
1000
uM

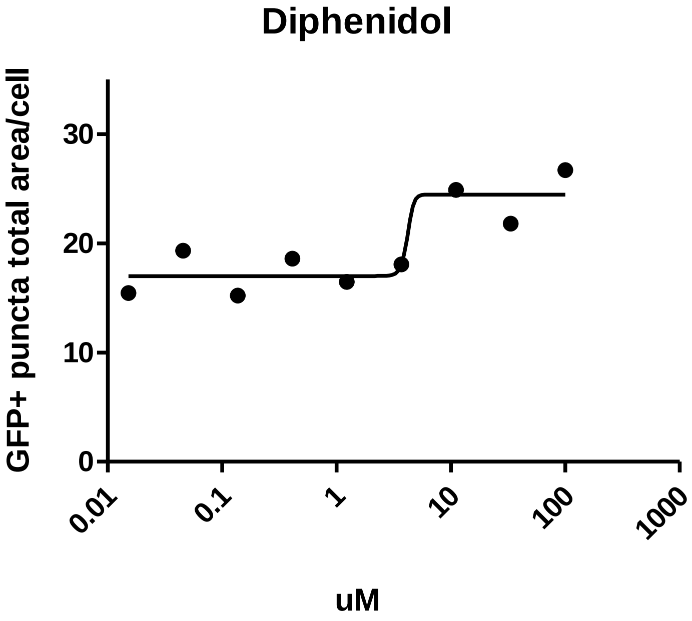
Diphenidol
GFP+ puncta total area/cell
0
10
20
30
0.01
0.1
1
10
100
1000
uM

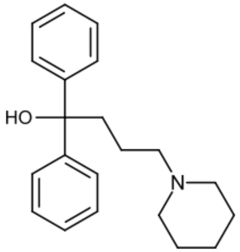
HO
N

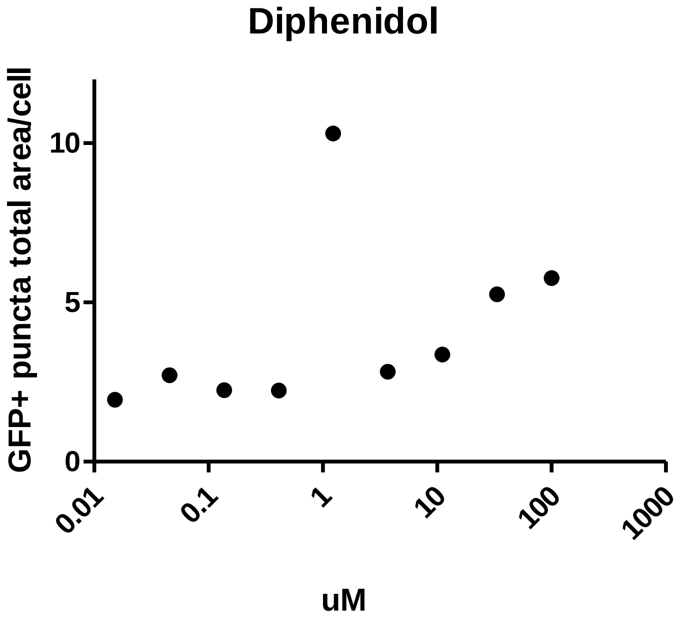
Diphenidol
GFP+ puncta total area/cell
0
5
10
0.01
0.1
1
10
100
1000
uM

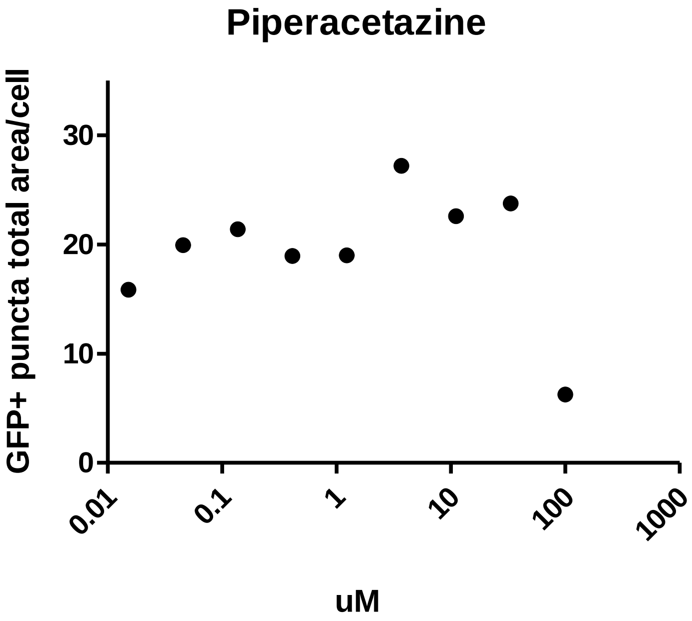
Piperacetazine
GFP+ puncta total area/cell
0
10
20
30
0.01
0.1
1
10
100
1000
uM

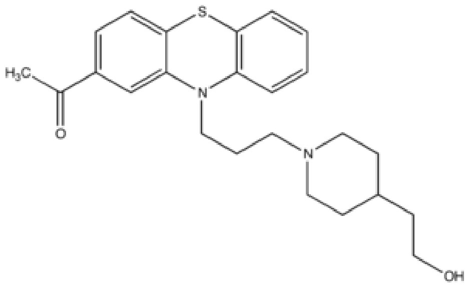
S
N
N
OH
O

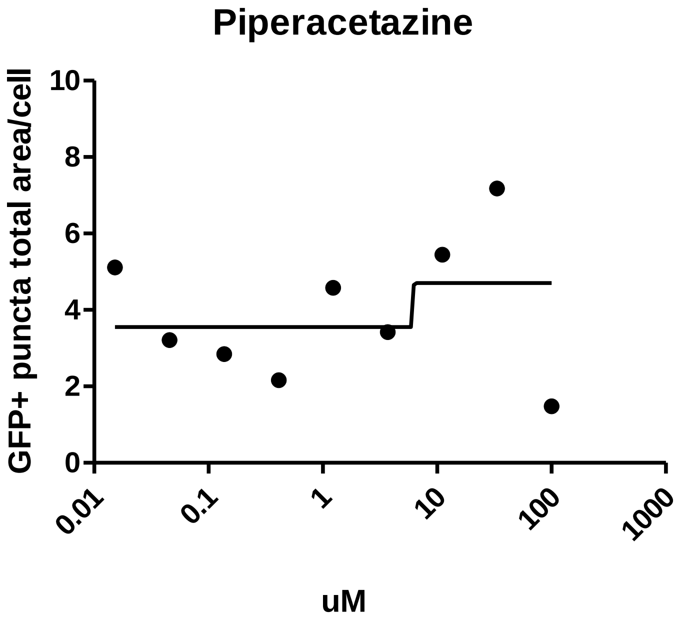
Piperacetazine
GFP+ puncta total area/cell
0
2
4
6
8
10
0.01
0.1
1
10
100
1000
uM

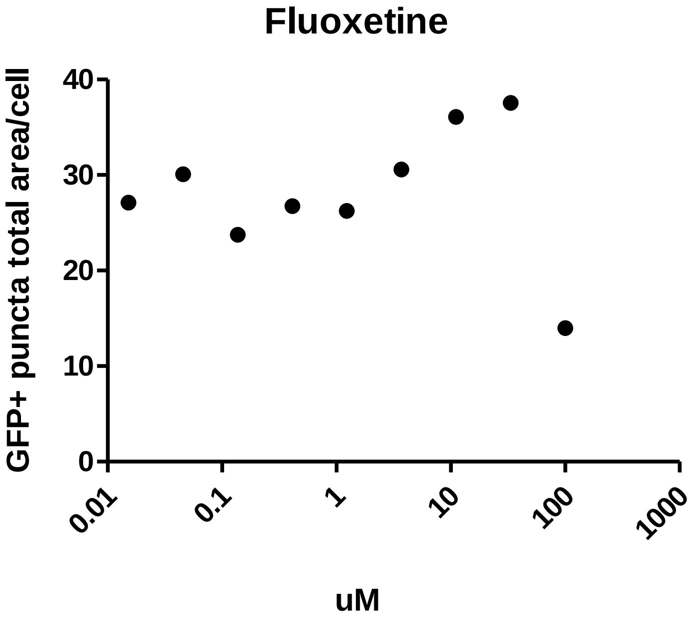
Fluoxetine
GFP+ puncta total area/cell
0
10
20
30
40
0.01
0.1
1
10
100
1000
uM

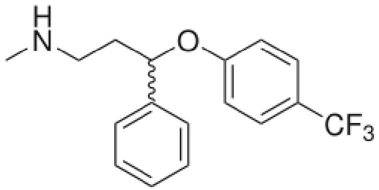
H
N
O

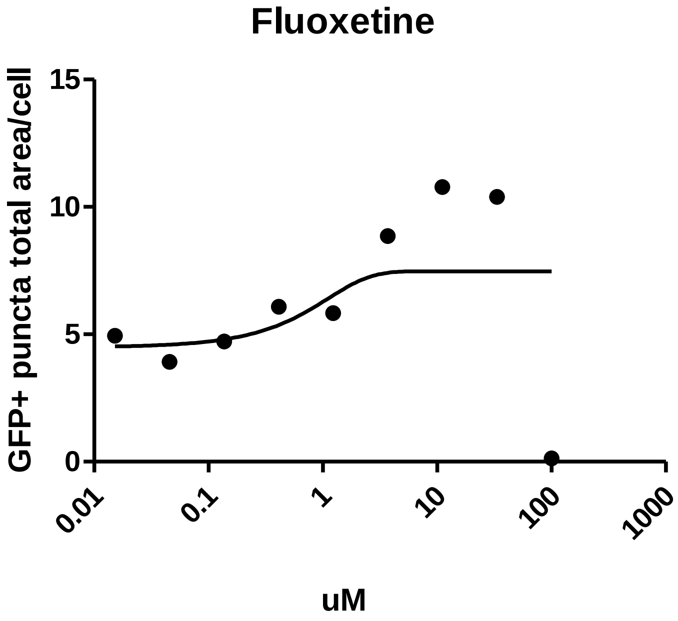
Fluoxetine
GFP+ puncta total area/cell
0
5
10
15
0.01
0.1
1
10
100
1000
uM

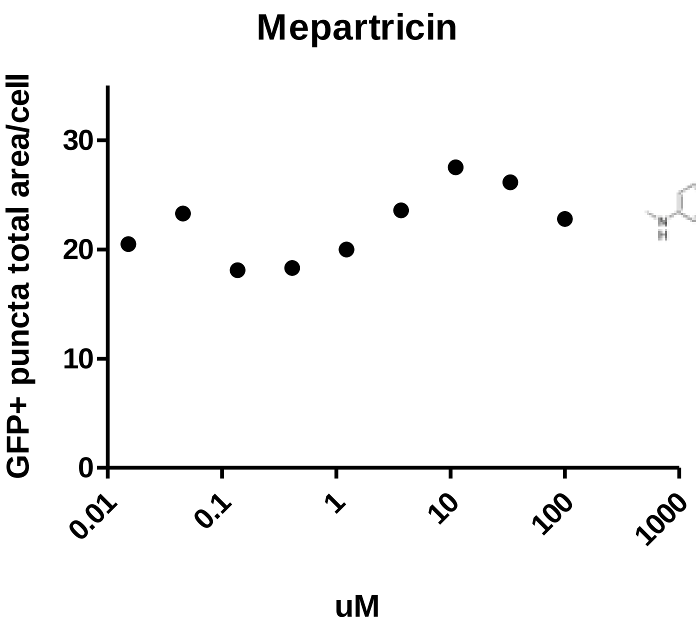
Mepartricin
GFP+ puncta total area/cell
0
10
20
30
0.01
0.1
1
10
100
1000
uM

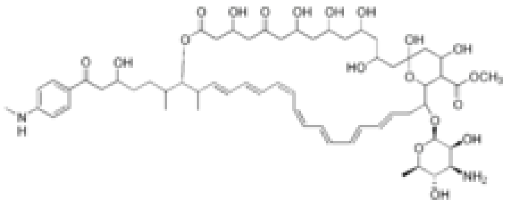

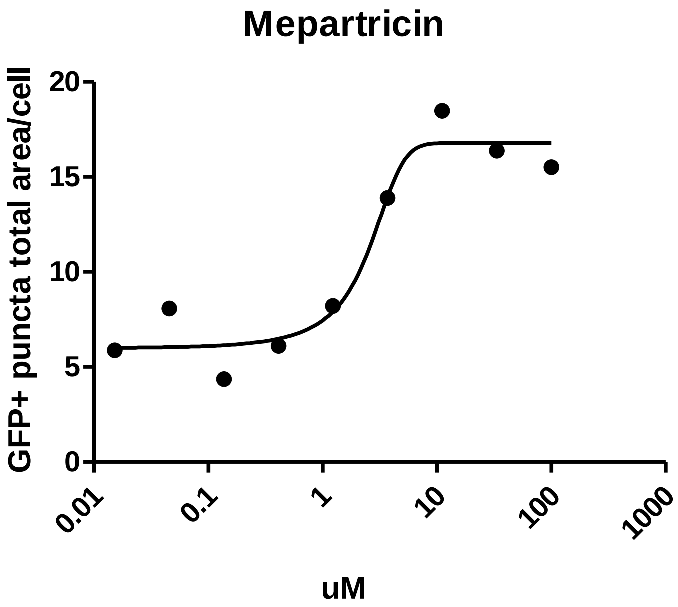
Mepartricin
GFP+ puncta total area/cell
0
5
10
15
20
0.01
0.1
1
10
100
1000
uM

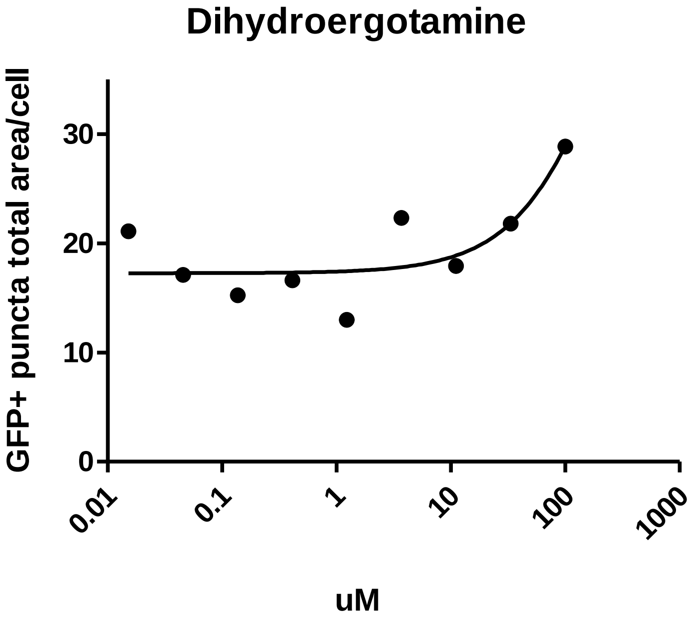
Dihydroergotamine
GFP+ puncta total area/cell
0
10
20
30
0.01
0.1
1
10
100
1000
uM

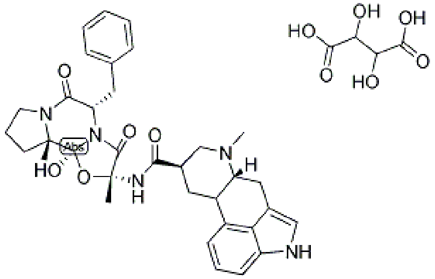

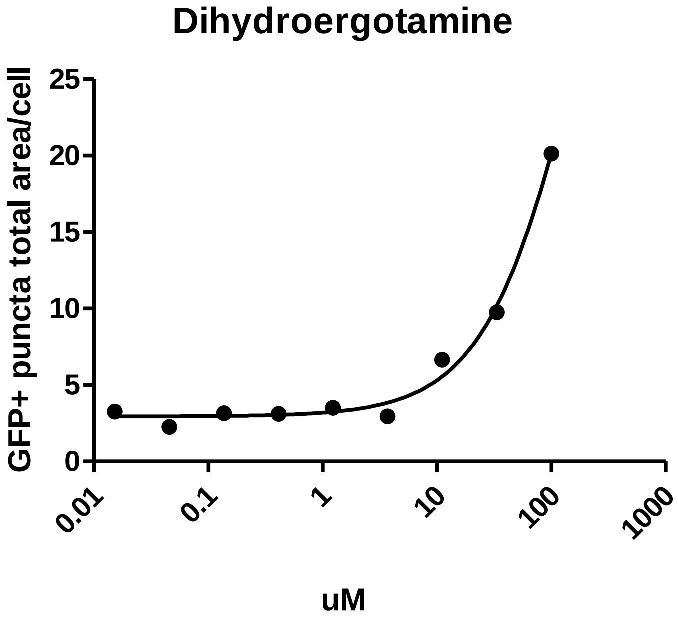
Dihydroergotamine
GFP+ puncta total area/cell
0
5
10
15
20
25
0.01
0.1
1
10
100
1000
uM

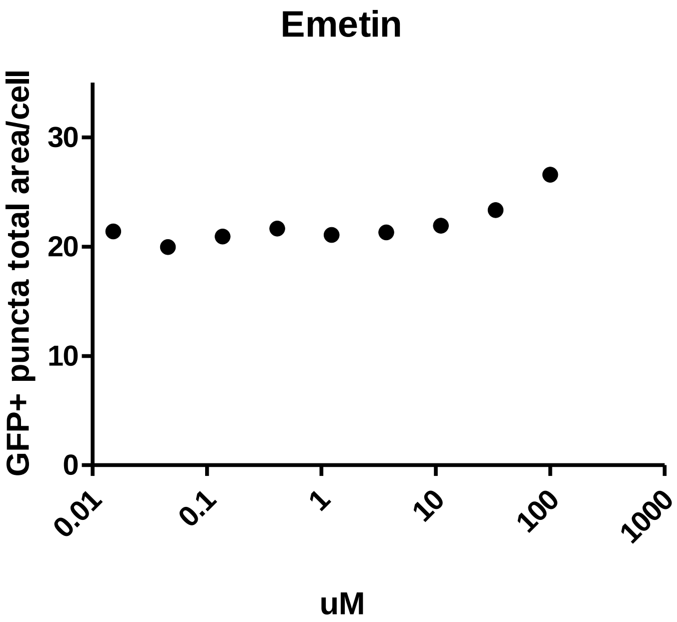
Emetin
GFP+ puncta total area/cell
0
10
20
30
0.01
0.1
1
10
100
1000
uM

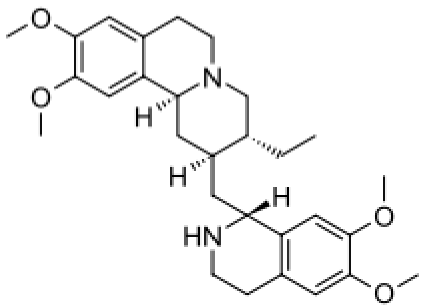
O
O
N
H
H
H
HN
O
O

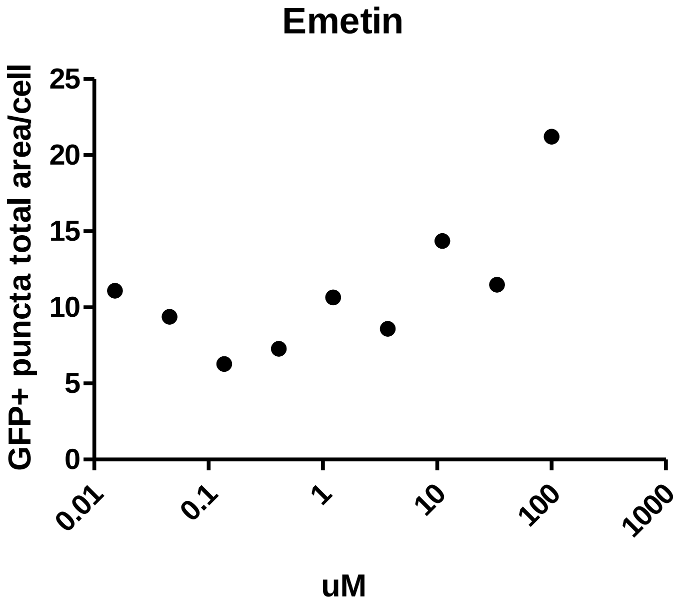
Emetin
GFP+ puncta total area/cell
0
5
10
15
20
25
0.01
0.1
1
10
100
1000
uM

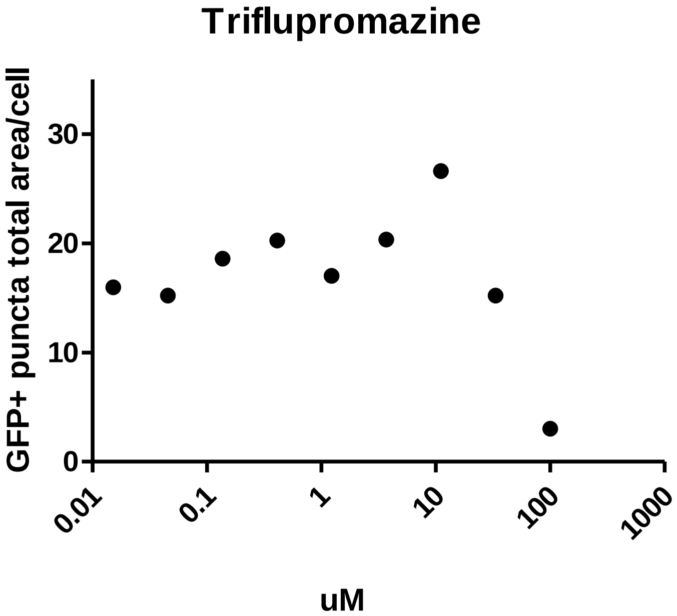
Triflupromazine
GFP+ puncta total area/cell
0
10
20
30
0.01
0.1
1
10
100
1000
uM

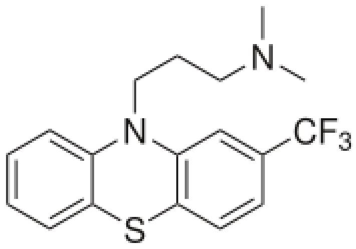
N
N
CF3
S

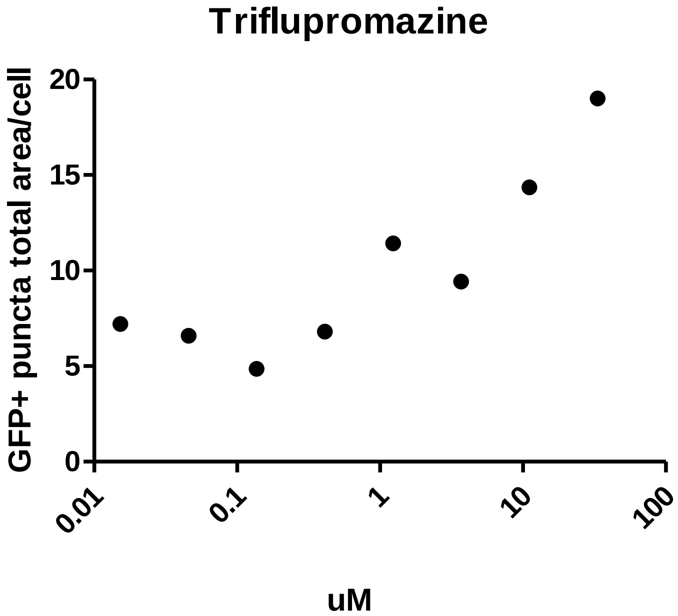
Triflupromazine
GFP+ puncta total area/cell
0
5
10
15
20
0.01
0.1
1
10
100
uM

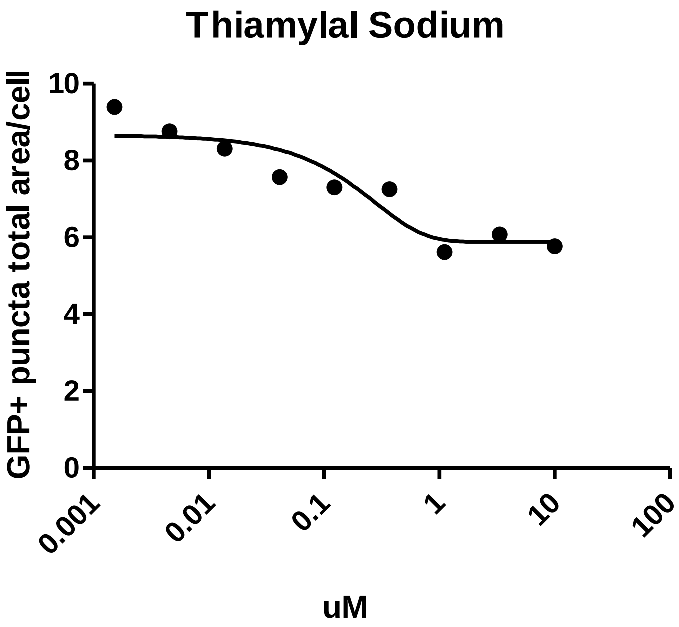
Thiamylal Sodium
GFP+ puncta total area/cell
0
2
4
6
8
10
0.001
0.01
0.1
1
10
100
uM

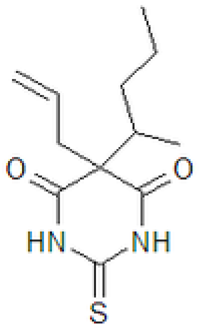
O
O
HN
NH
S

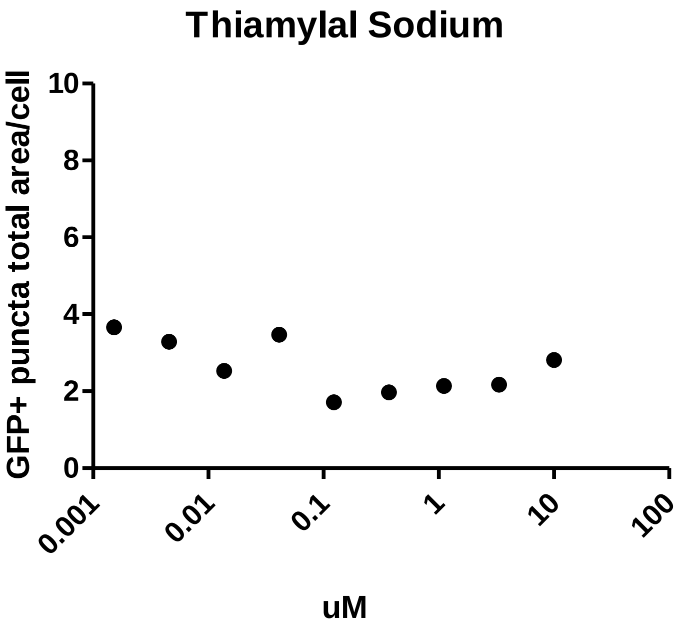
Thiamylal Sodium
GFP+ puncta total area/cell
0
2
4
6
8
10
0.001
0.01
0.1
1
10
100
uM

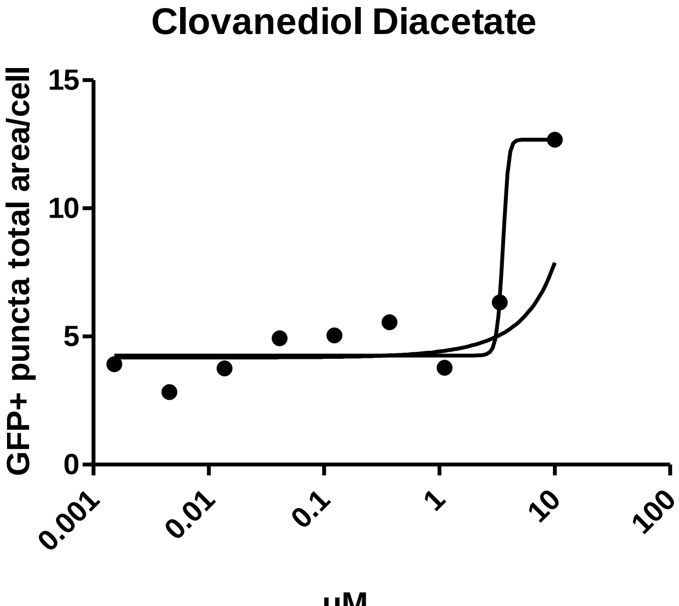
Clovanediol Diacetate
GFP+ puncta total area/cell
0
5
10
15
0.001
0.01
0.1
1
10
100
uM

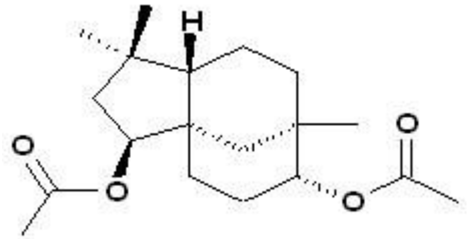
H
O
O
O
O

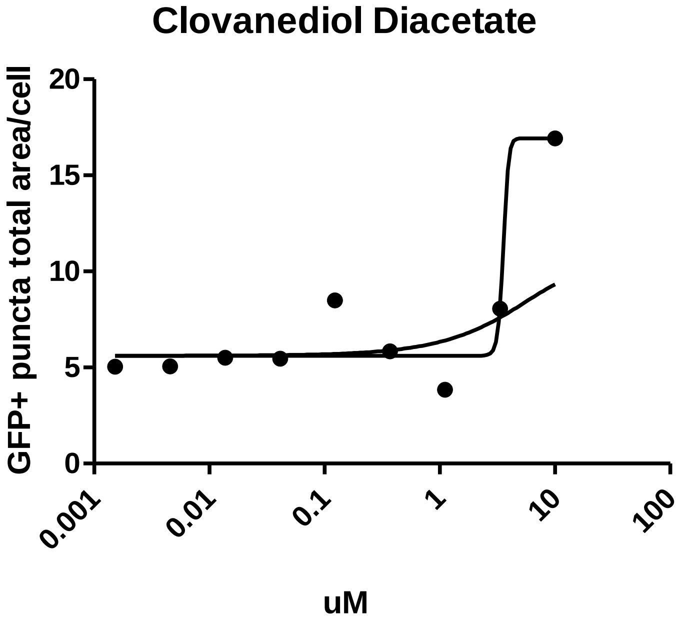
Clovanediol Diacetate
GFP+ puncta total area/cell
0
5
10
15
20
0.001
0.01
0.1
1
10
100
uM

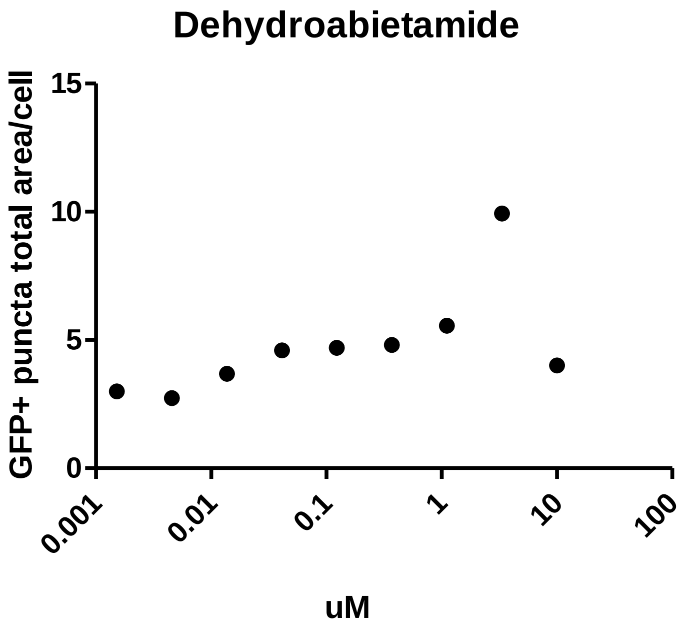
Dehydroabietamide
GFP+ puncta total area/cell
15
10
5
0
0.001
0.01
0.1
1
10
100
uM

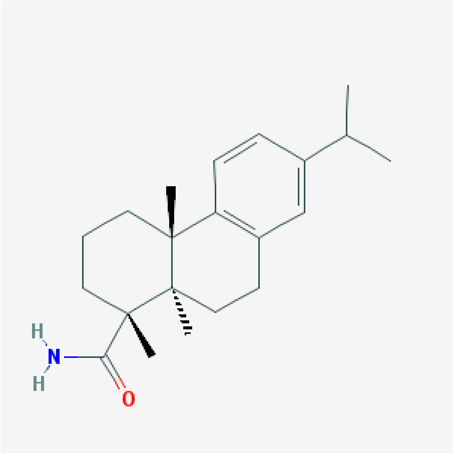
H
N
H
O

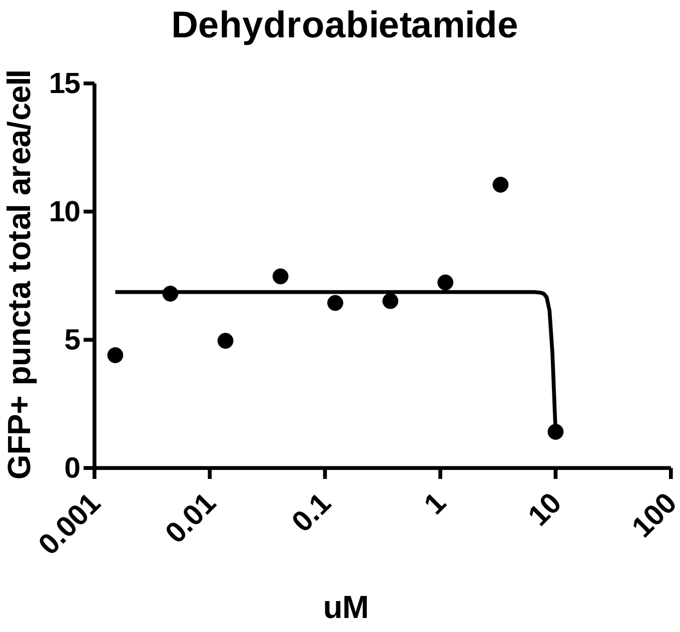
Dehydroabietamide
GFP+ puncta total area/cell
15
10
5
0
0.001
0.01
0.1
1
10
100
uM

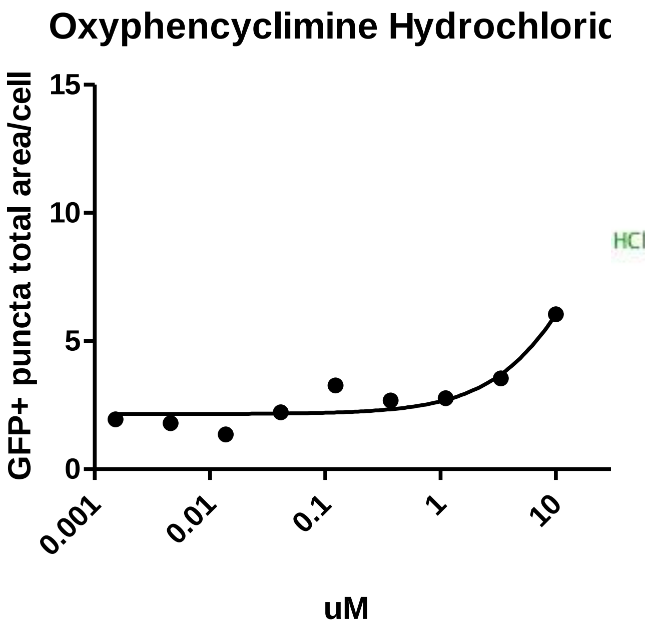
Oxyphencyclimine Hydrochloric
GFP+ puncta total area/cell
15
10
5
0
0.001
0.01
0.1
1
10
uM

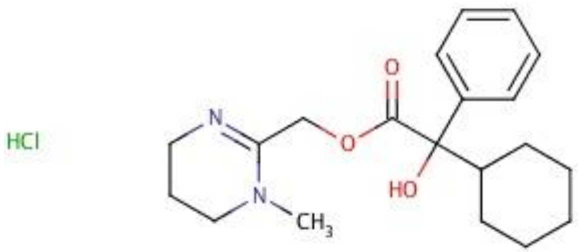
HCl
N
N
CH3
O
O
HO

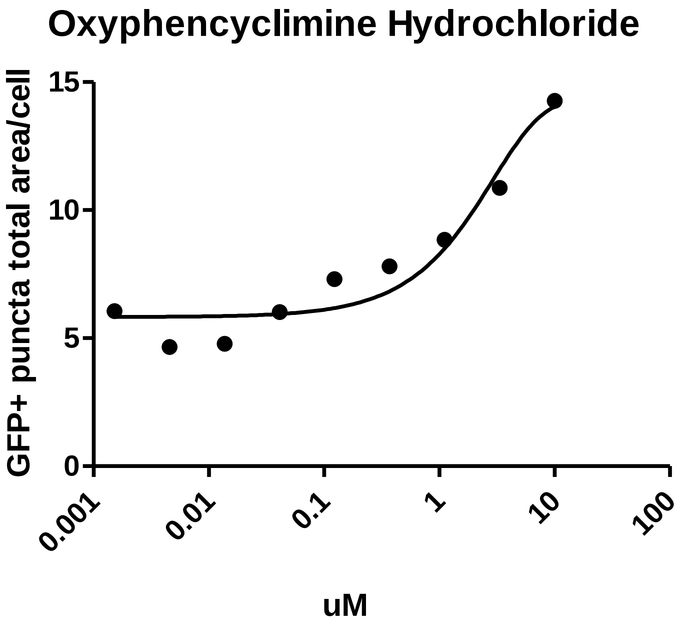
Oxyphencyclimine Hydrochloride
GFP+ puncta total area/cell
15
10
5
0
0.001
0.01
0.1
1
10
100
uM

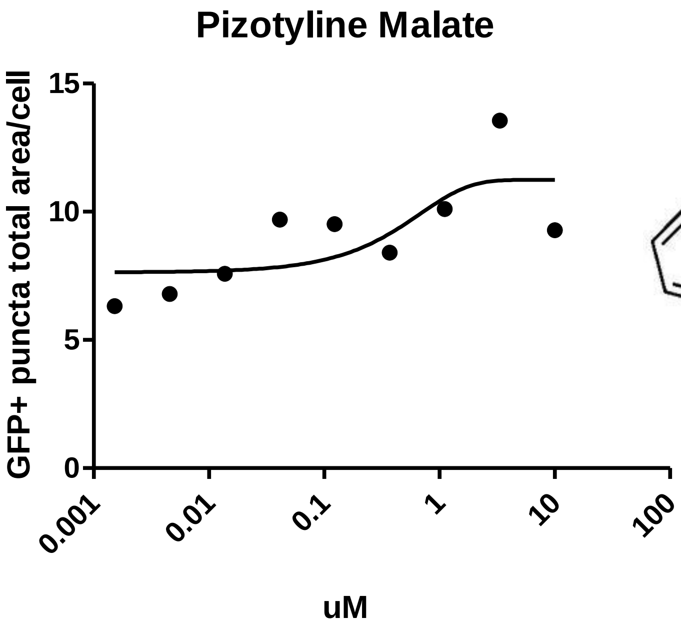
Pizotyline Malate
GFP+ puncta total area/cell
15
10
5
0
0.001
0.01
0.1
1
10
100
uM

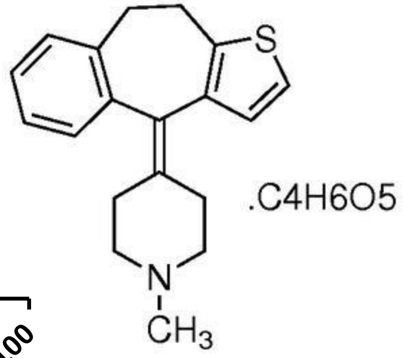
S
N
CH3
.C4H6O5

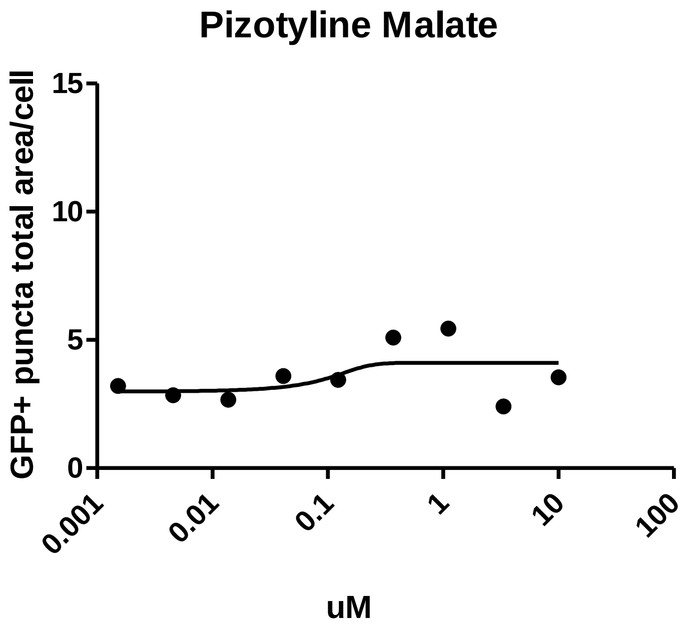
Pizotyline Malate
GFP+ puncta total area/cell
15
10
5
0
0.001
0.01
0.1
1
10
100
uM

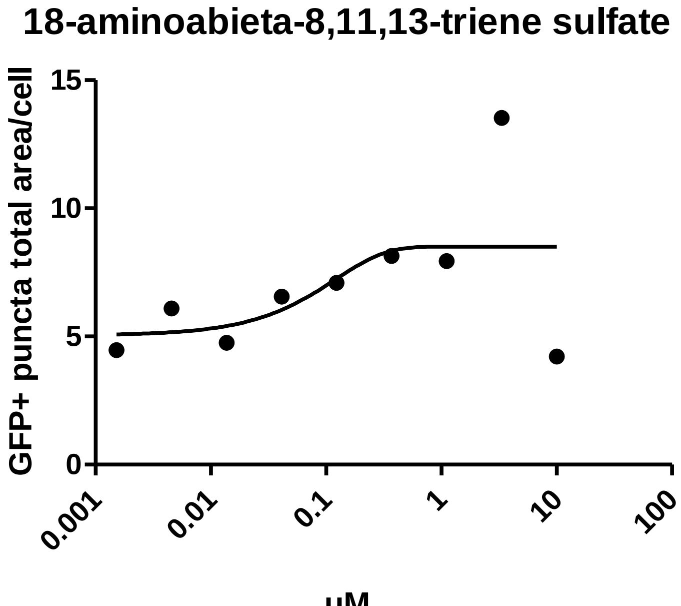
18-aminoabieta-8,11,13-triene sulfate
GFP+ puncta total area/cell
15
10
5
0
0.001
0.01
0.1
1
10
100
uM

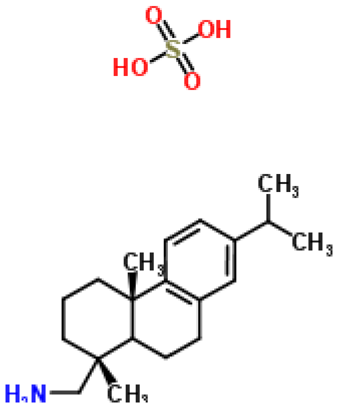
O
OH
HO
S
O
CH3
CH3
CH3
H2N
CH3

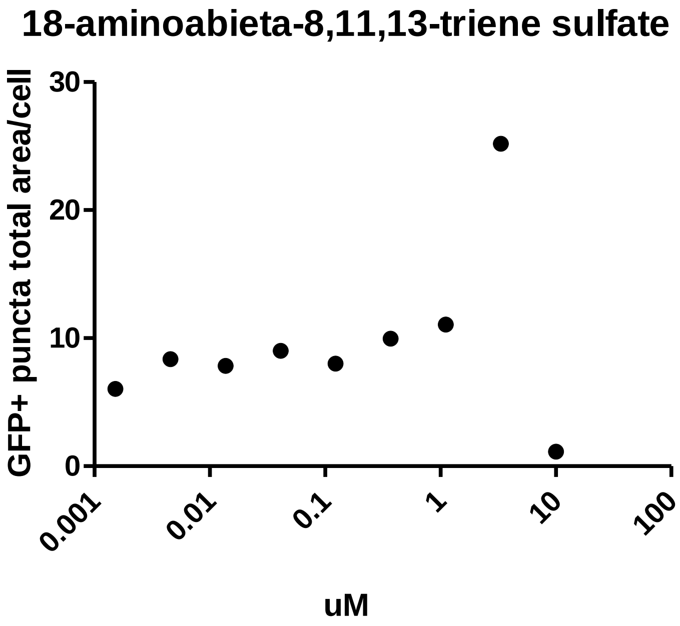
18-aminoabieta-8,11,13-triene sulfate
GFP+ puncta total area/cell
30
20
10
0
0.001
0.01
0.1
1
10
100
uM

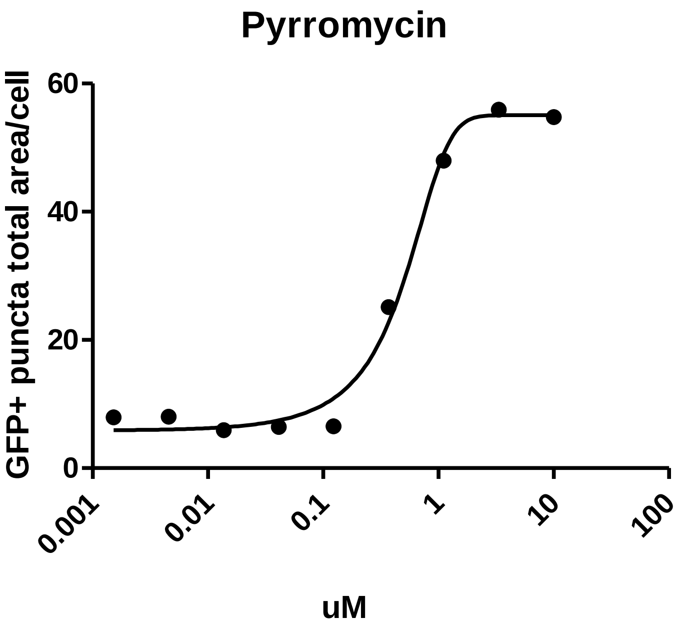
Pyrromycin
GFP+ puncta total area/cell
60
40
20
0
0.001
0.01
0.1
1
10
100
uM

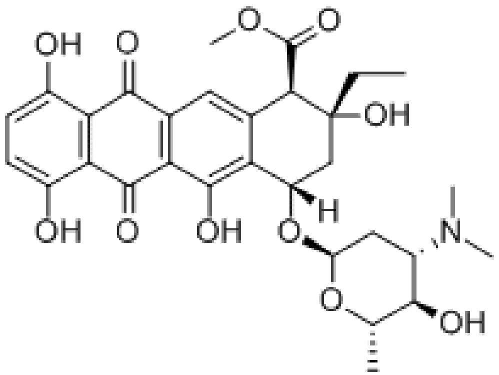
OH
O
OH
O
OH
O
O
OH
H
O
N
O
OH

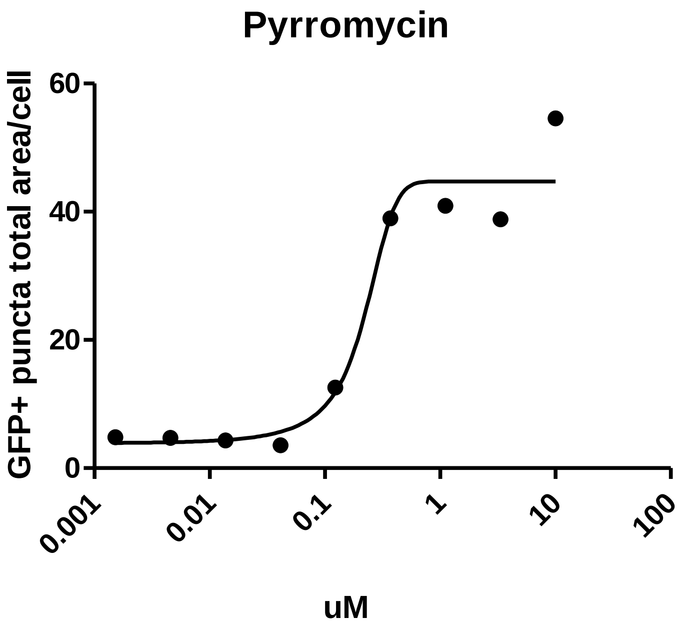
Pyrromycin
GFP+ puncta total area/cell
60
40
20
0
0.001
0.01
0.1
1
10
100
uM

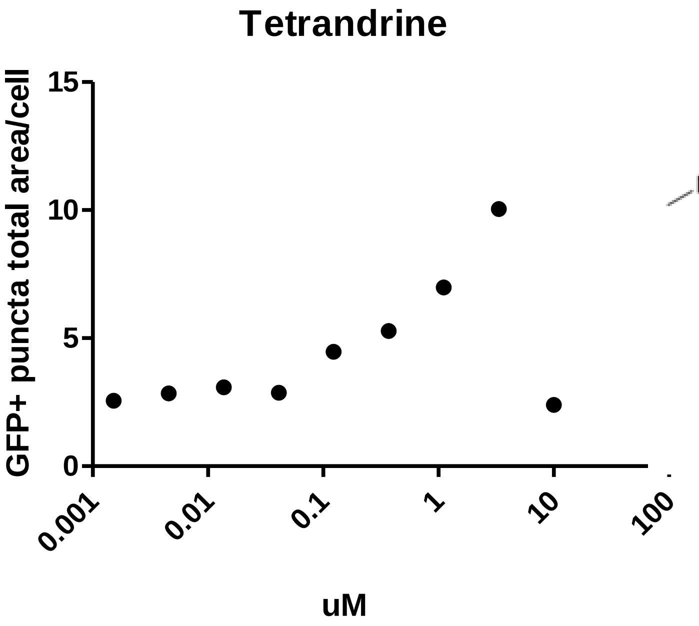
Tetrandrine
GFP+ puncta total area/cell
15
10
5
0
0.001
0.01
0.1
1
10
100
uM

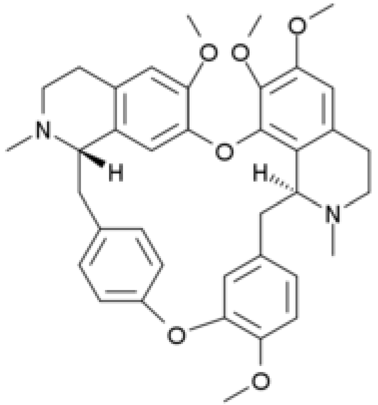
O
O
O
N
H
O
H
N
O
O

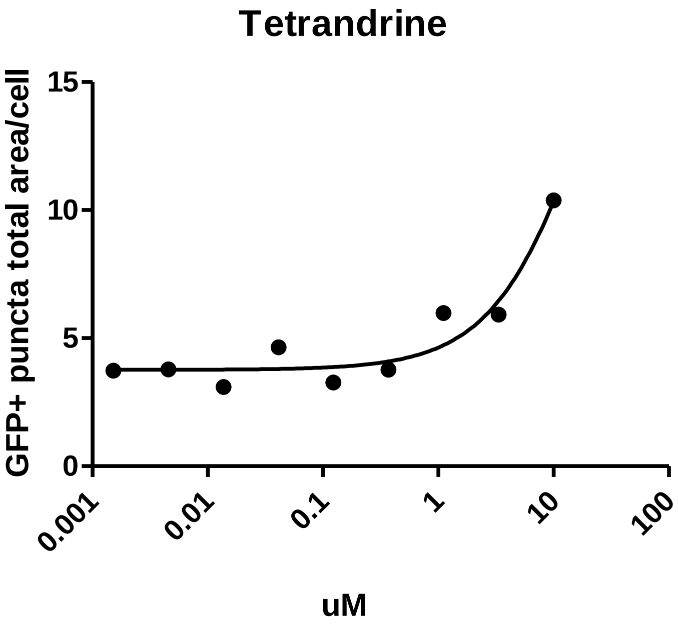
Tetrandrine
GFP+ puncta total area/cell
15
10
5
0
0.001
0.01
0.1
1
10
100
uM

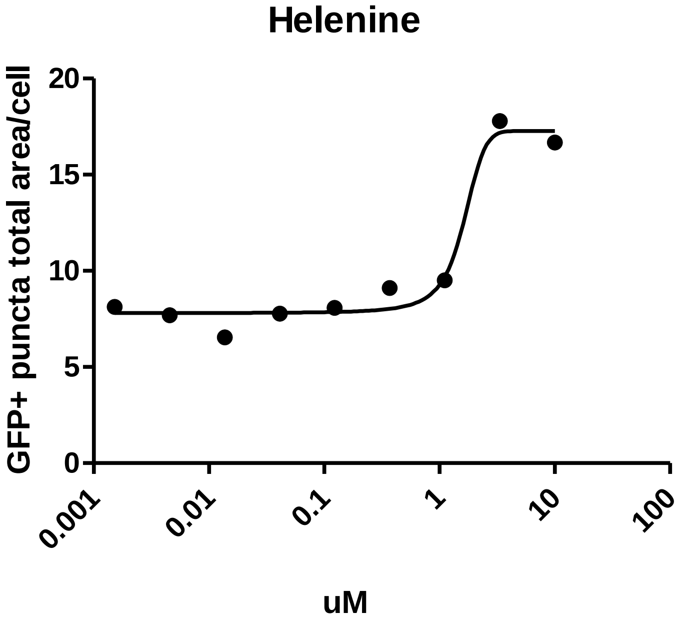
Helenine
GFP+ puncta total area/cell
0
5
10
15
20
0.001
0.01
0.1
1
10
100
uM

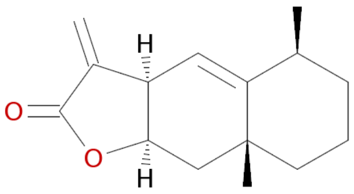

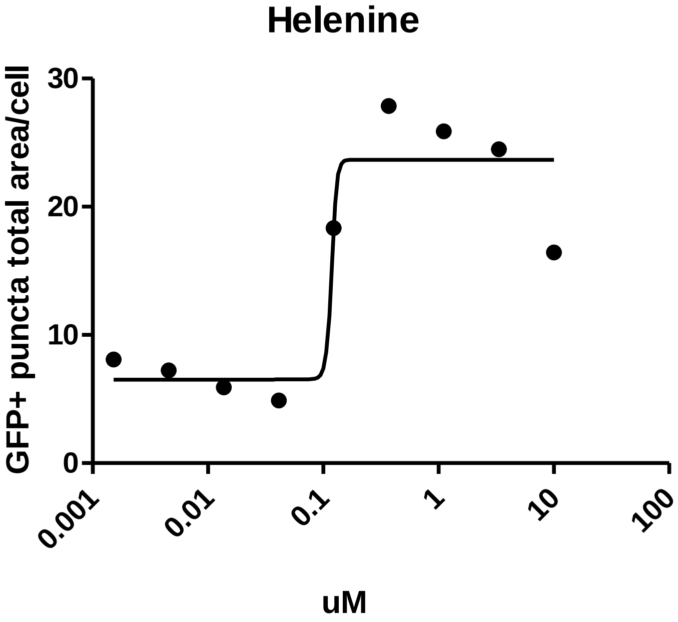
Helenine
GFP+ puncta total area/cell
0
10
20
30
0.001
0.01
0.1
1
10
100
uM

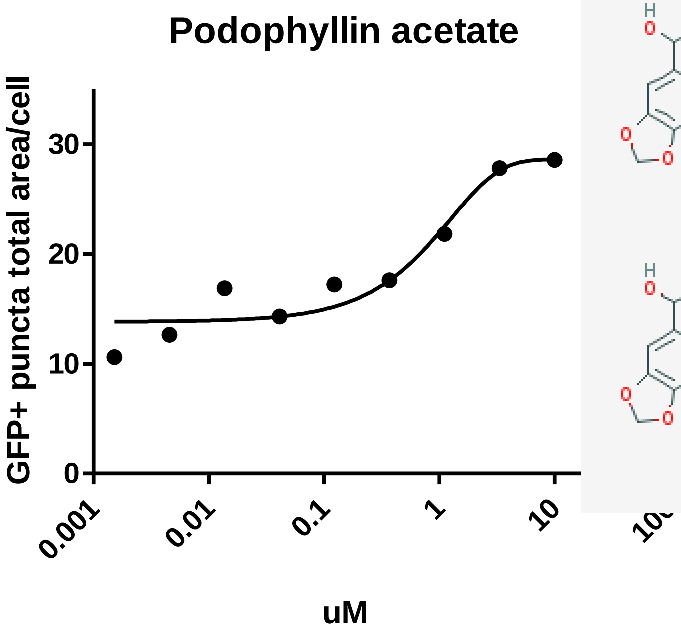
Podophyllin acetate
GFP+ puncta total area/cell
0
10
20
30
0.001
0.01
0.1
1
10
uM

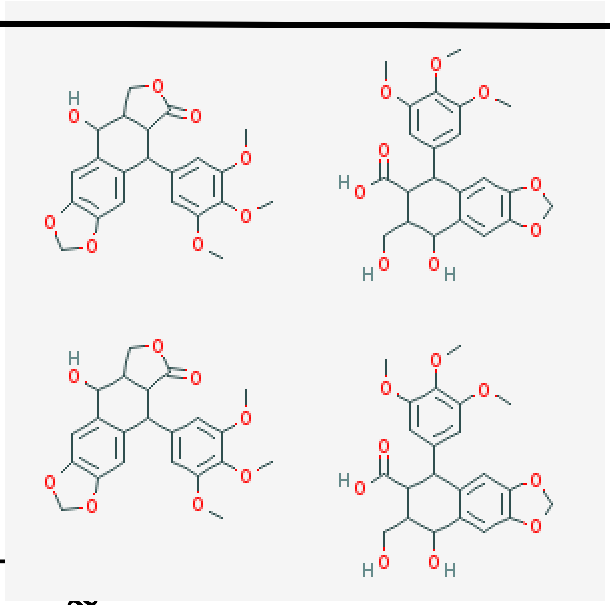

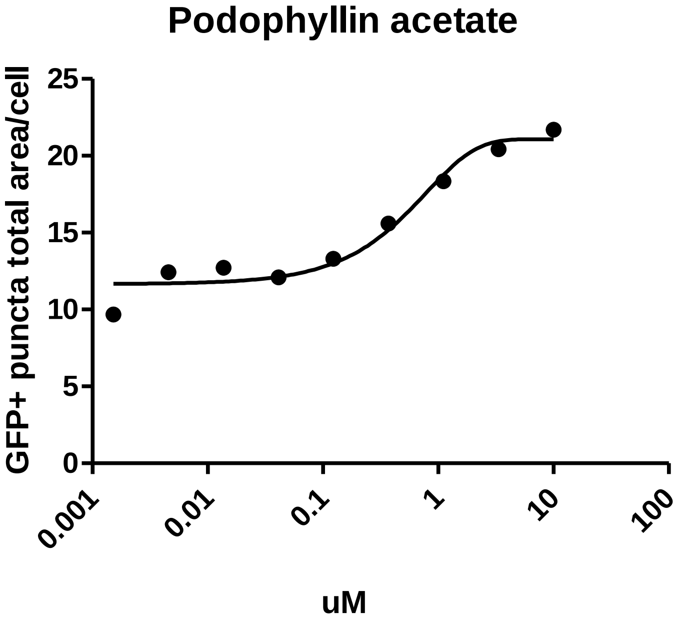
Podophyllin acetate
GFP+ puncta total area/cell
0
5
10
15
20
25
0.001
0.01
0.1
1
10
100
uM

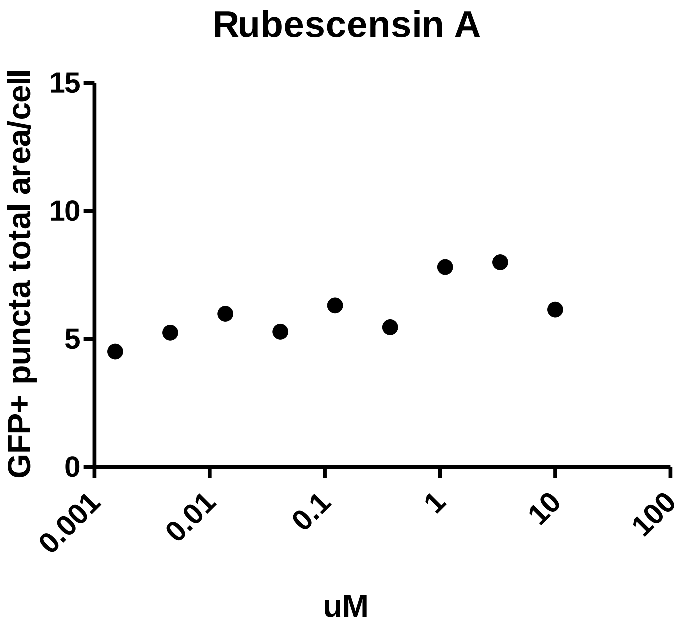
Rubescensin A
GFP+ puncta total area/cell
0
5
10
15
0.001
0.01
0.1
1
10
100
uM

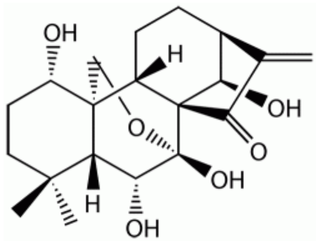
OH
H
OH
O
O
OH
H
OH

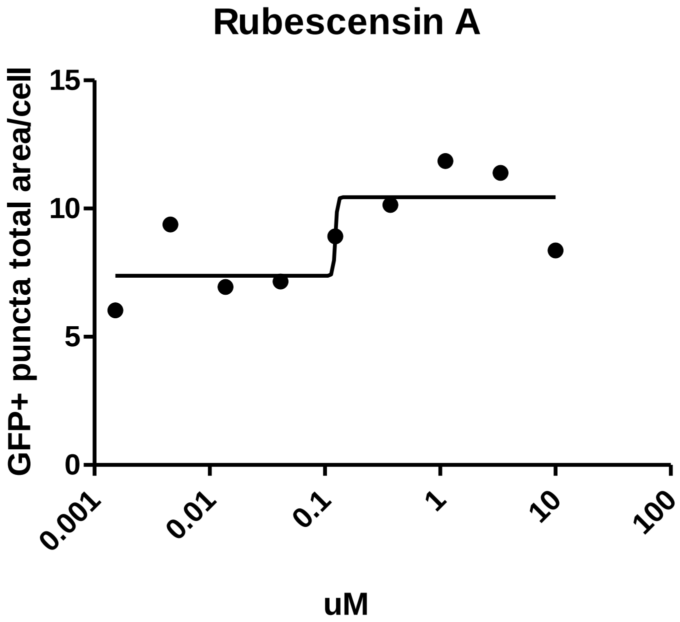
Rubescensin A
GFP+ puncta total area/cell
0
5
10
15
0.001
0.01
0.1
1
10
100
uM

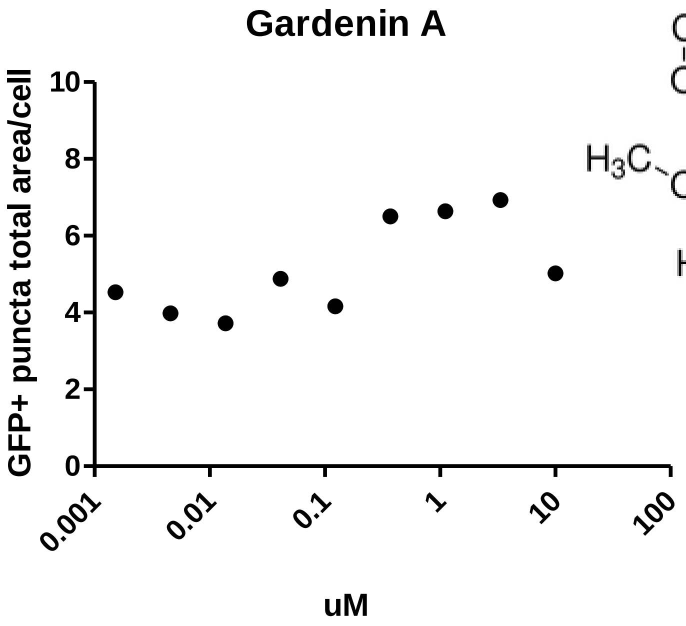
Gardenin A
GFP+ puncta total area/cell
0
2
4
6
8
10
0.001
0.01
0.1
1
10
100
uM

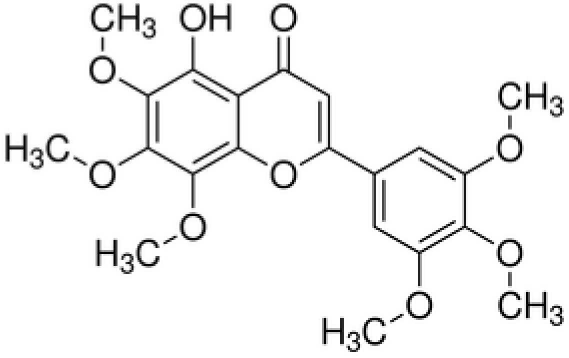
CH3
OH
O
O
H3C
O
O
O
H3C
CH3
O
O
CH3
O
H3C

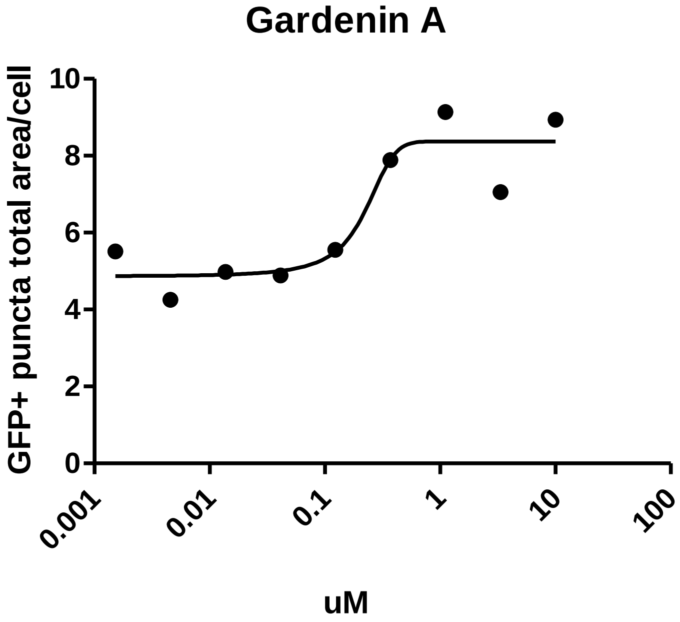
Gardenin A
GFP+ puncta total area/cell
0
2
4
6
8
10
0.001
0.01
0.1
1
10
100
uM

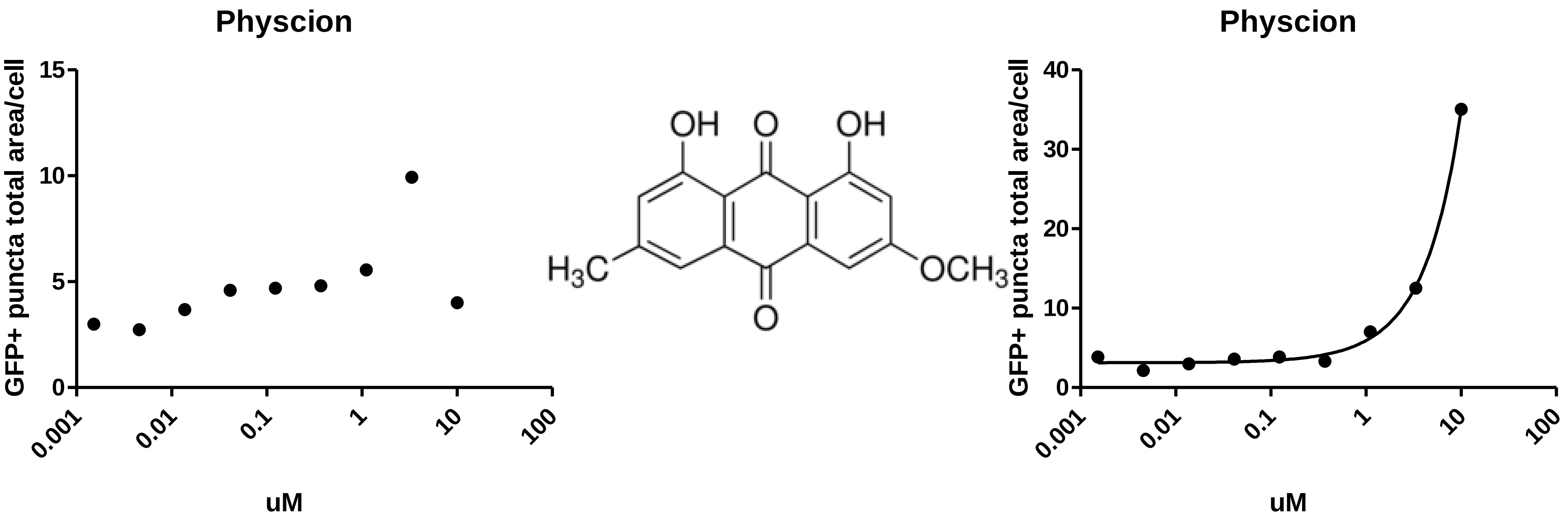
Physcion
GFP+ puncta total area/cell
15
10
5
0
0.001
0.01
0.1
1
10
100
uM
OH
O
OH
H_3C
OCH_3
O
Physcion
GFP+ puncta total area/cell
40
30
20
10
0
0.001
0.01
0.1
1
10
100
uM

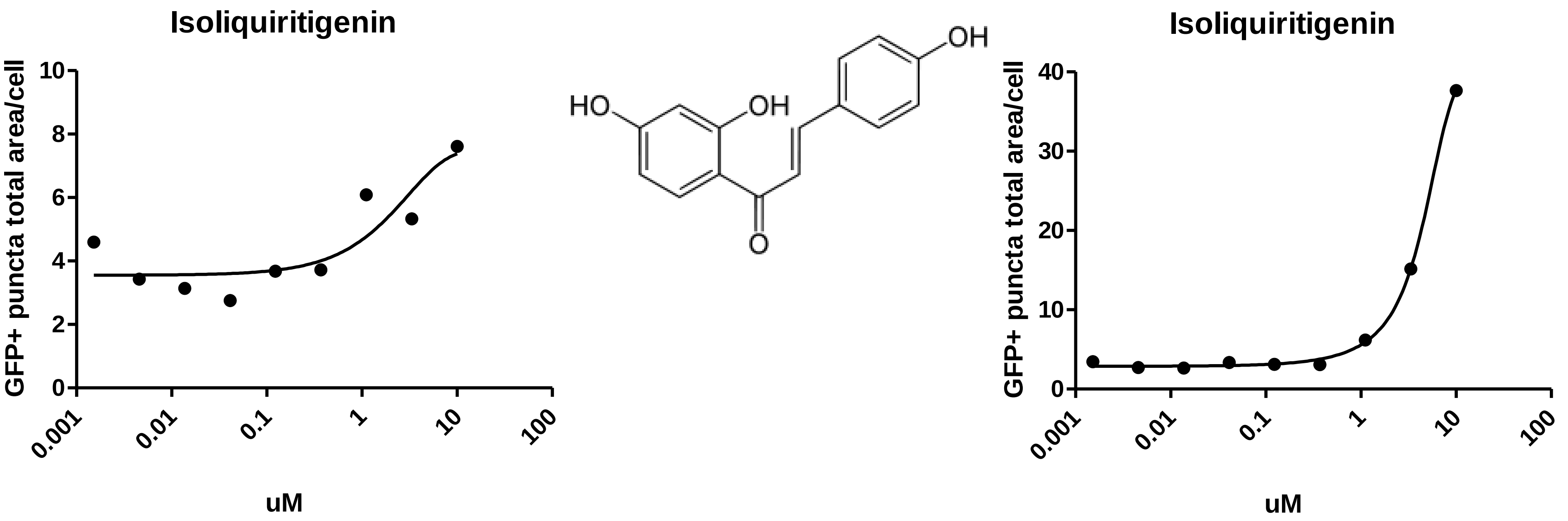
Isoliquiritigenin
GFP+ puncta total area/cell
10
8
6
4
2
0
0.001
0.01
0.1
1
10
100
uM
OH
HO
OH
O
Isoliquiritigenin
GFP+ puncta total area/cell
40
30
20
10
0
0.001
0.01
0.1
1
10
100
uM

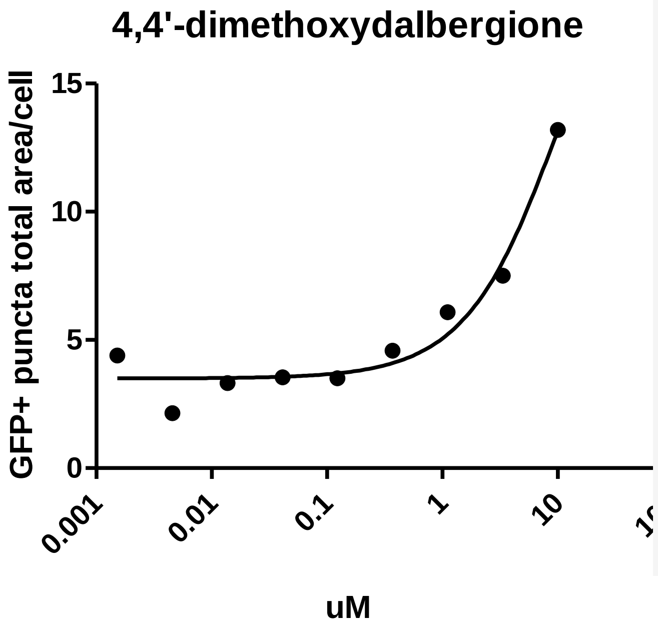
4,4'-dimethoxydalbergione
GFP+ puncta total area/cell
0
5
10
15
0.001
0.01
0.1
1
10
uM

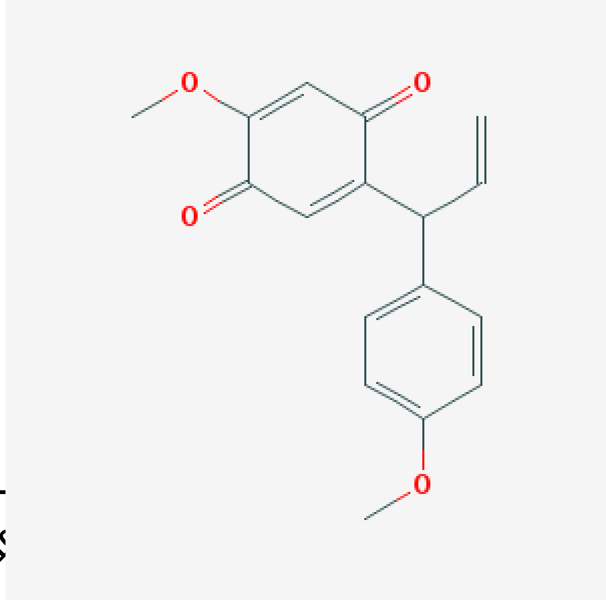
O
O
O
O

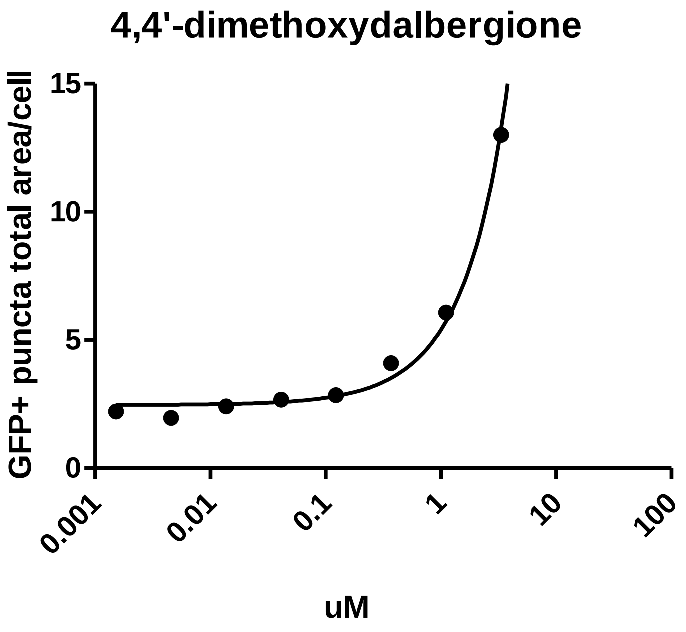
4,4'-dimethoxydalbergione
GFP+ puncta total area/cell
0
5
10
15
0.001
0.01
0.1
1
10
100
uM

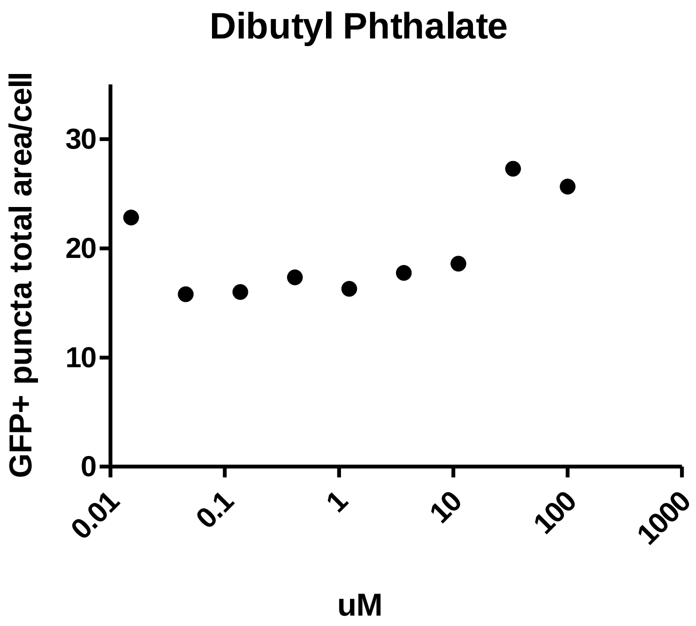
Dibutyl Phthalate
GFP+ puncta total area/cell
0
10
20
30
0.01
0.1
1
10
100
1000
uM

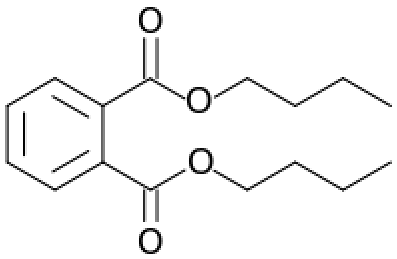
O
O
O
O

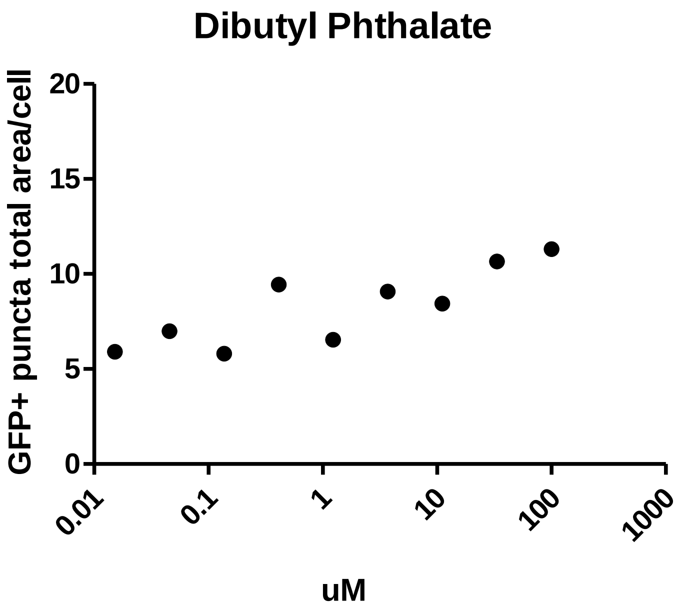
Dibutyl Phthalate
GFP+ puncta total area/cell
0
5
10
15
20
0.01
0.1
1
10
100
1000
uM

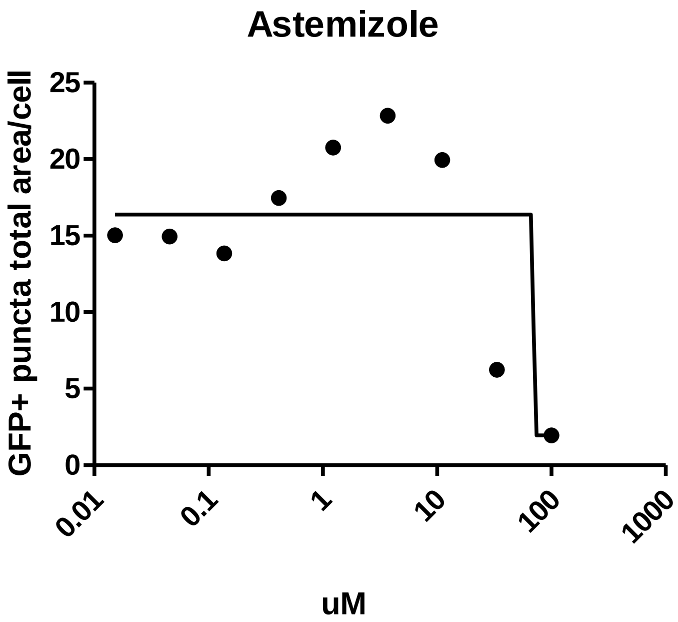
Astemizole
GFP+ puncta total area/cell
25
20
15
10
5
0
0.01
0.1
1
10
100
1000
uM

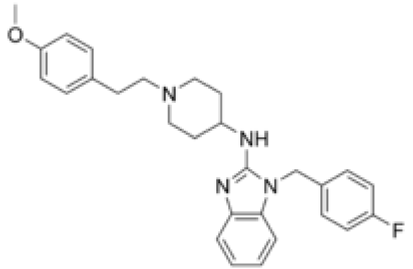
O
N
NH
N
N
F

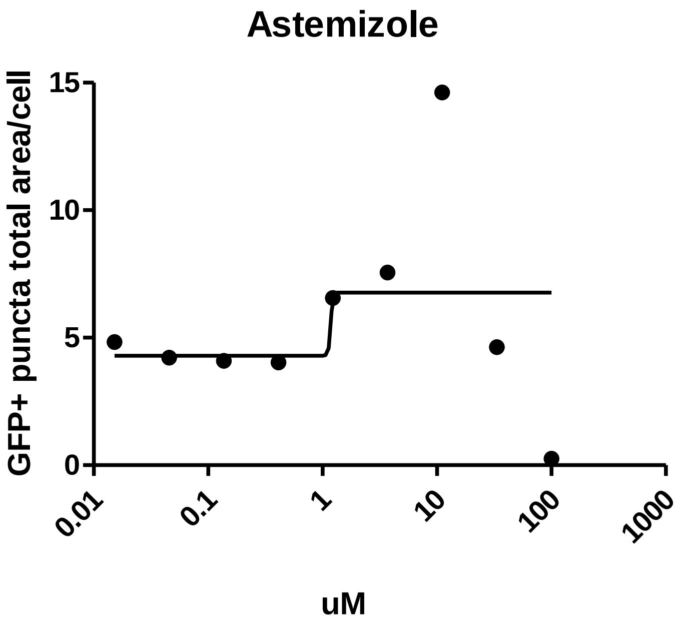
Astemizole
GFP+ puncta total area/cell
15
10
5
0
0.01
0.1
1
10
100
1000
uM

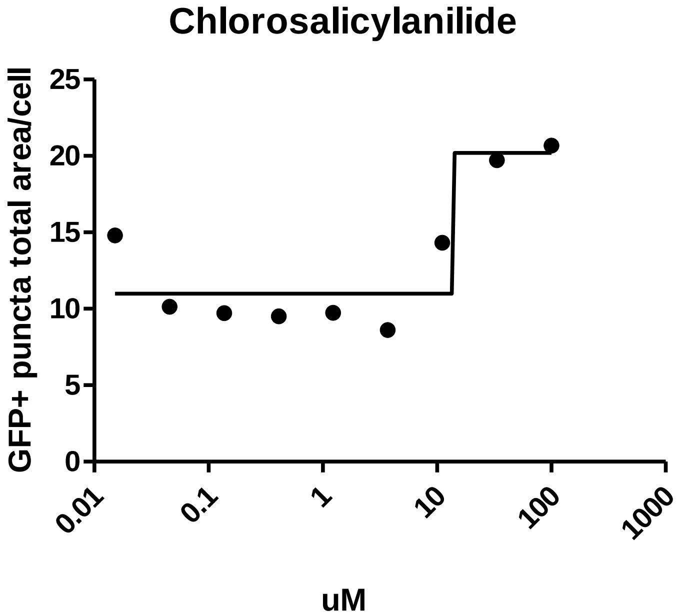
Chlorosalicylanilide
GFP+ puncta total area/cell
25
20
15
10
5
0
0.01
0.1
1
10
100
1000
uM

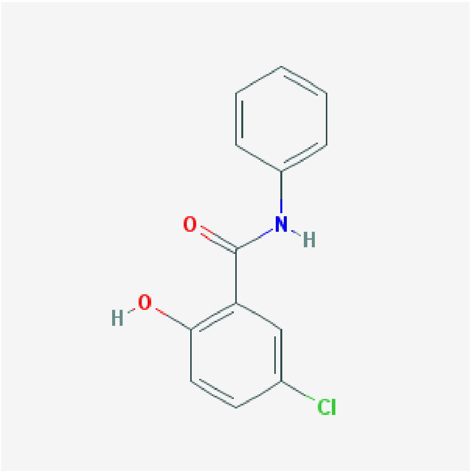
O
N
H
H
O
Cl

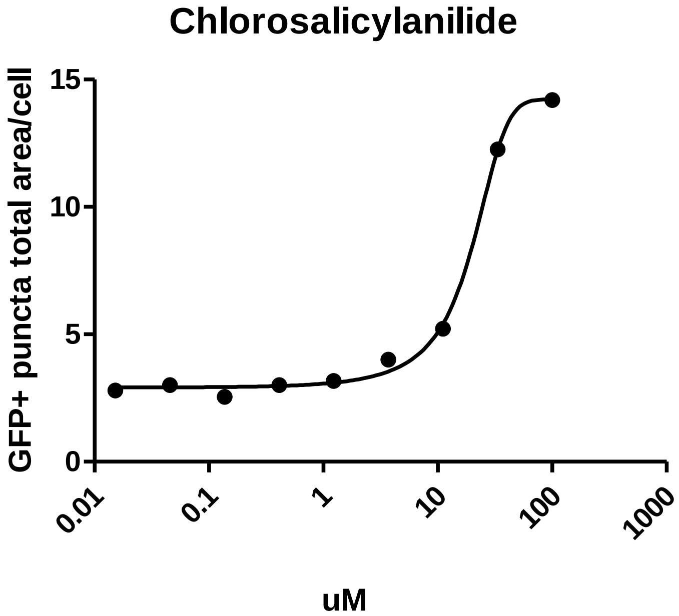
Chlorosalicylanilide
GFP+ puncta total area/cell
15
10
5
0
0.01
0.1
1
10
100
1000
uM

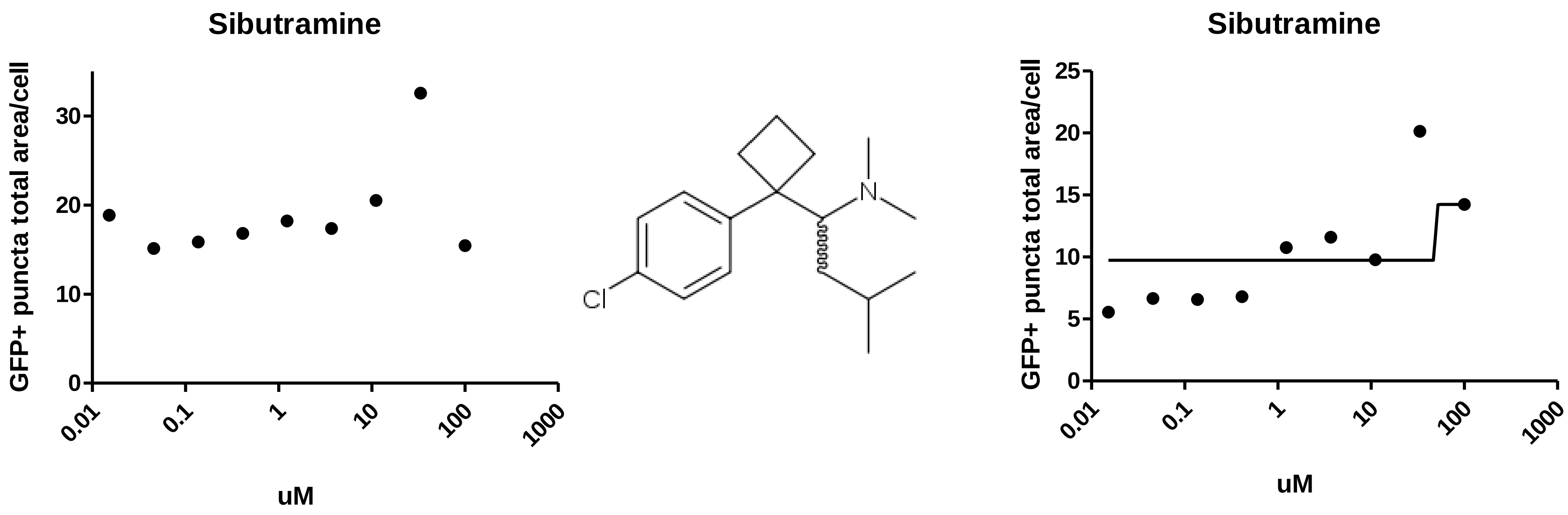
Sibutramine
GFP+ puncta total area/cell
uM
0
10
20
30
0.01
0.1
1
10
100
1000
Cl
N
Sibutramine
GFP+ puncta total area/cell
uM
0
5
10
15
20
25
0.01
0.1
1
10
100
1000

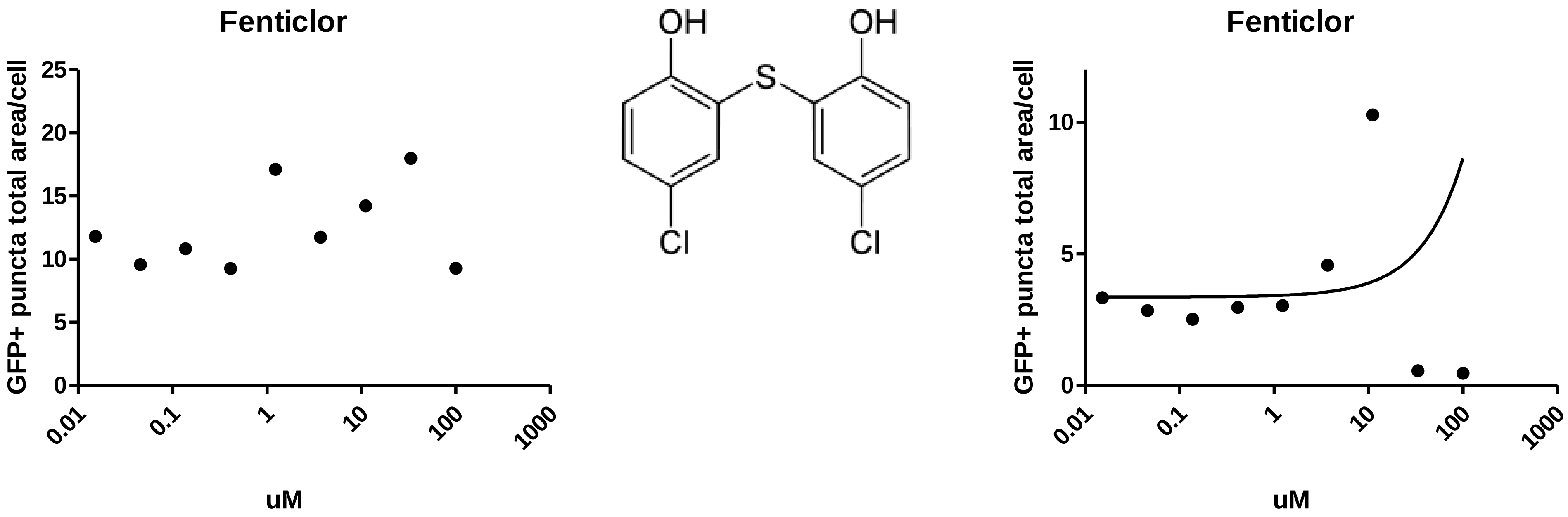
Fenticlor
GFP+ puncta total area/cell
uM
0
5
10
15
20
25
0.01
0.1
1
10
100
1000
OH
OH
S
Cl
Cl
Fenticlor
GFP+ puncta total area/cell
uM
0
5
10
0.01
0.1
1
10
100
1000

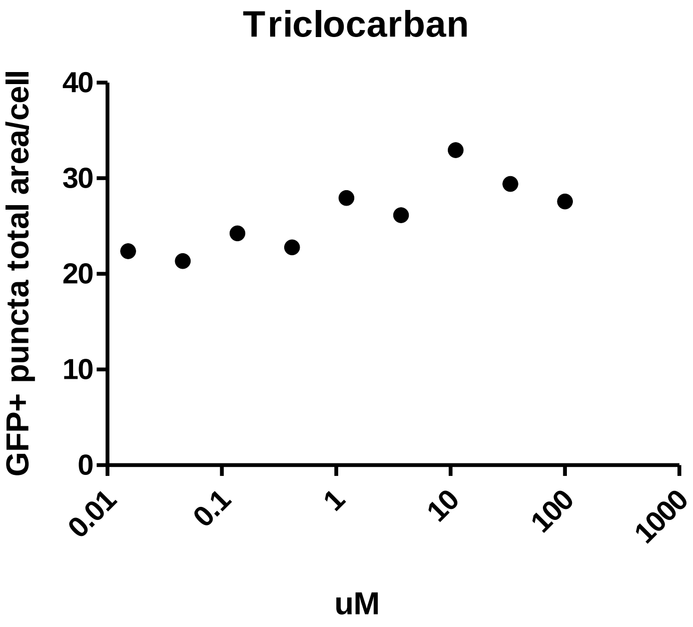
Triclocarban
GFP+ puncta total area/cell
0
10
20
30
40
0.01
0.1
1
10
100
1000
uM

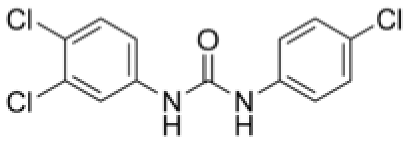
Cl
Cl
O
N
H
N
H
Cl

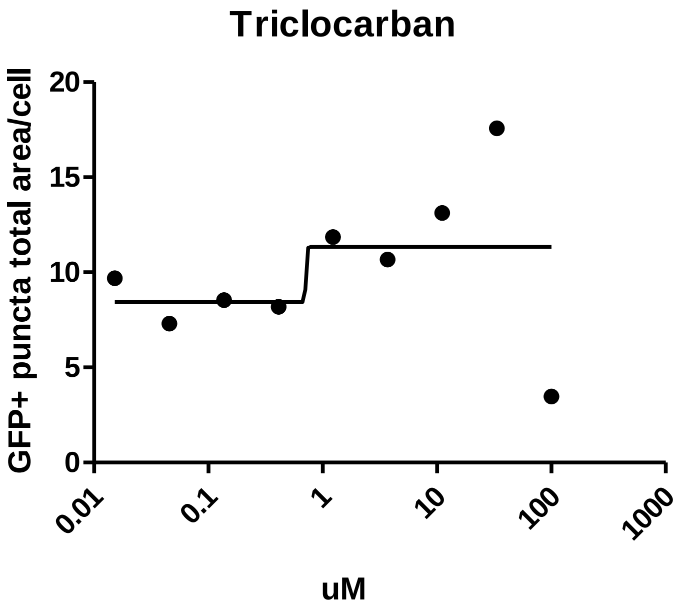
Triclocarban
GFP+ puncta total area/cell
0
5
10
15
20
0.01
0.1
1
10
100
1000
uM

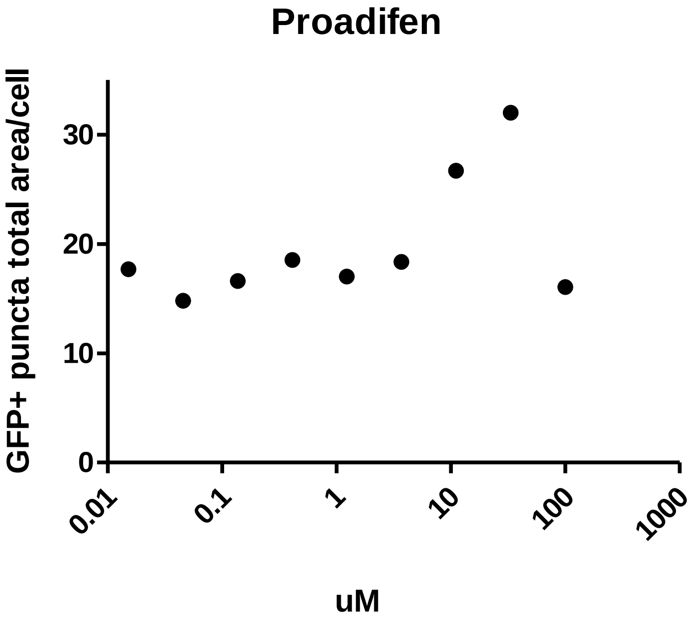
Proadifen
GFP+ puncta total area/cell
0
10
20
30
0.01
0.1
1
10
100
1000
uM

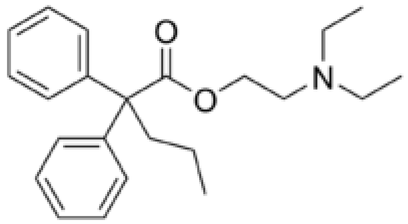
O
O
N

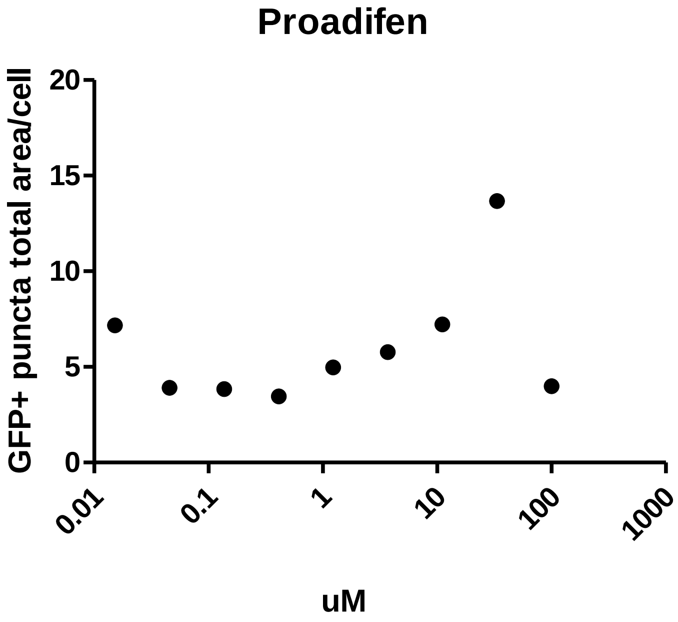
Proadifen
GFP+ puncta total area/cell
0
5
10
15
20
0.01
0.1
1
10
100
1000
uM

pp242 (mTOR inhibitor; positive control)

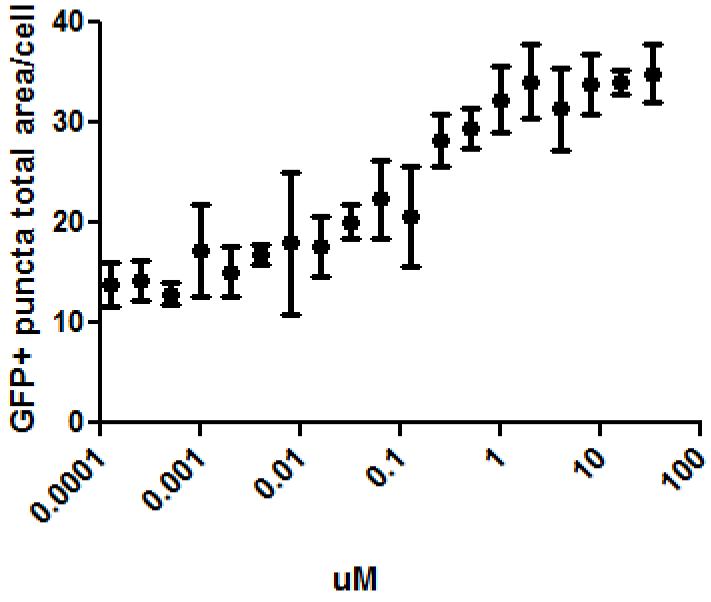

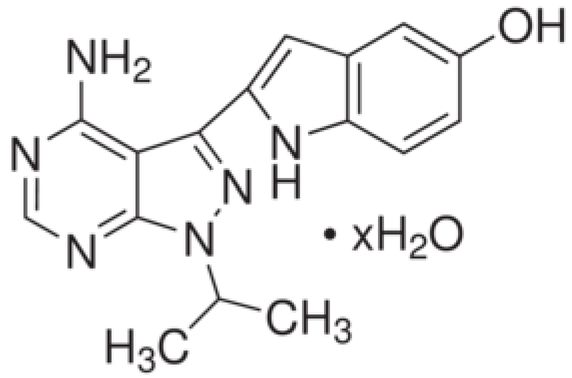

Supplementary Figure 3

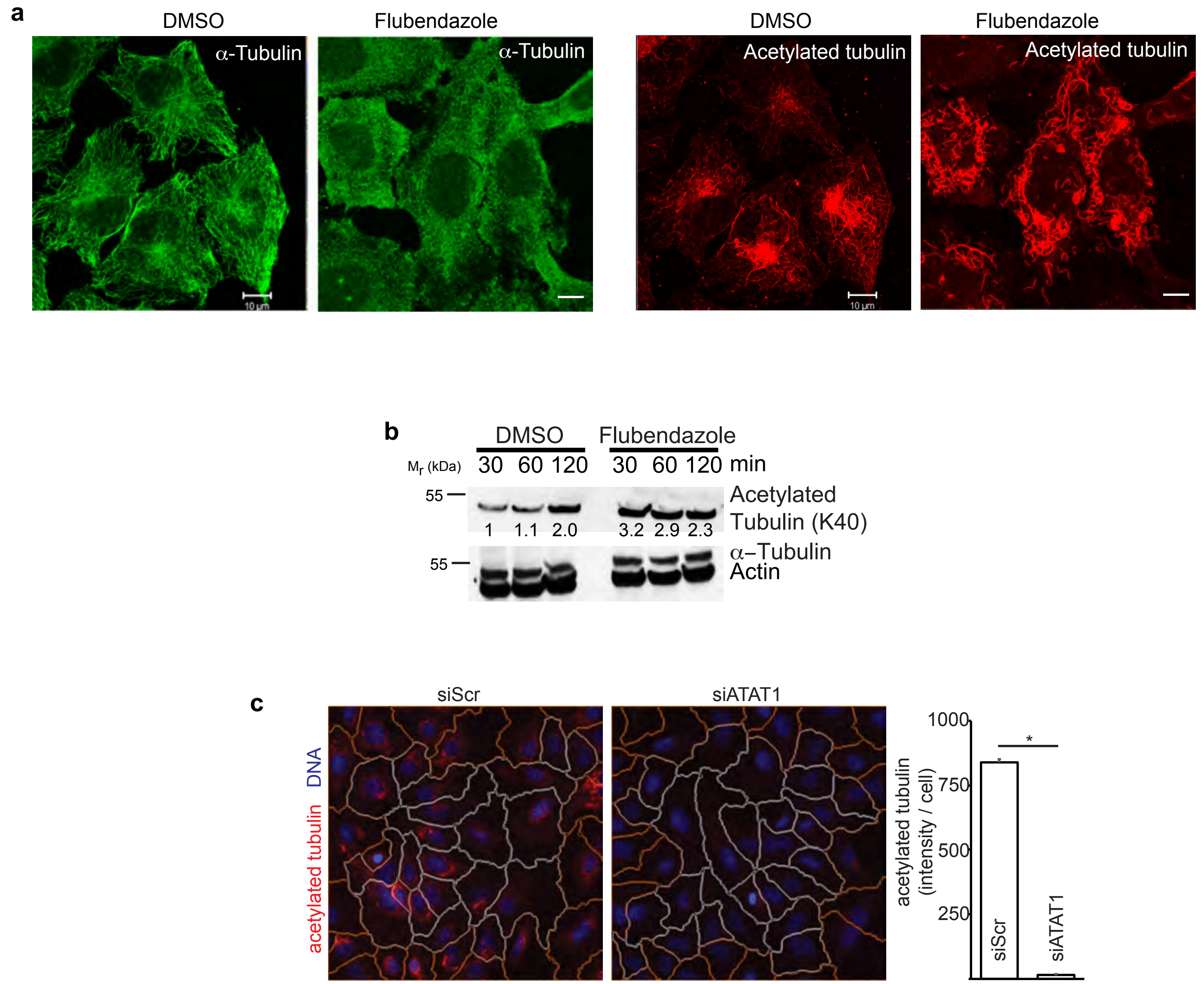

Supplementary Fig. 3. Flubendazole disrupts dynamic microtubules and increases cellular content of acetylated microtubules. (**a**) Confocal images, HeLa treated with DMSO or flubendazole (1 h, 5 μg/ml). Immunofluorescence, antibodies to acetylated tubulin (Sigma clone 6-11B-1) and α-tubulin (Abcam, YOL1/34). (**b**) Immunoblot analysis of tubulin acetylation in cells treated with flubendazole. (**c**) High content imaging analysis of acetylated tubulin abundance in HeLa cells subjected to control or ATAT1 knockdown. White mask, automatically determined cell outlines. Orange mask, cells rejected from analysis. *, $p < 0.05$ (t test), n = 3. Scale bars 10 μm.

Supplementary Figure 4

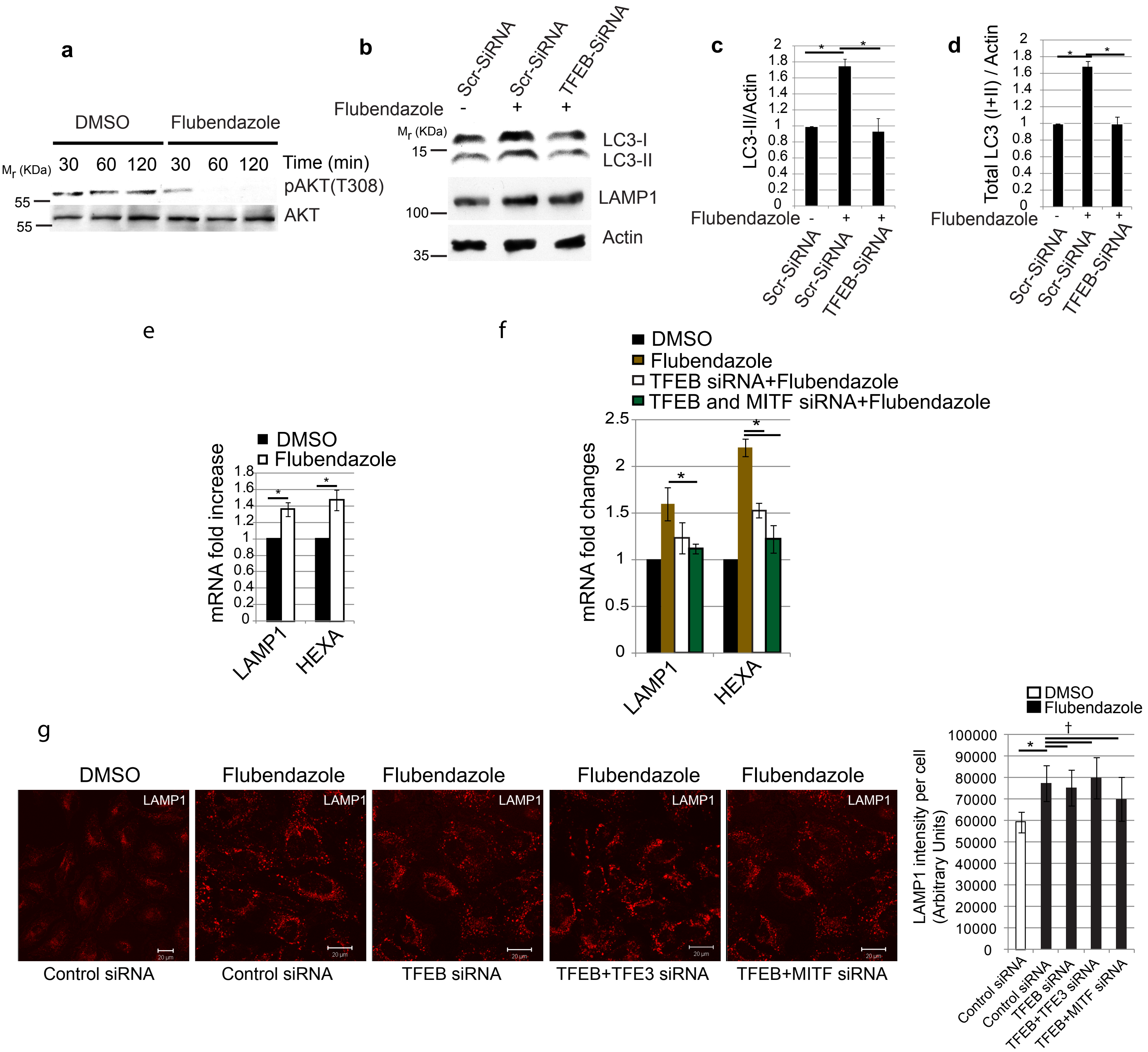

Supplementary Fig. 4. (**a**) Flubendazole inhibits Akt phosphorylation. Western blot of lysates from DMSO- or 5 µg/ml flubendazole-treated HeLa cells were probed with antibodies as indicated. (**b**) HeLa cells treated with DMSO or flubendazole (5 µg/ml) were lysed and subjected to immunoblotting with LC3B, LAMP1 and actin antibody. (**c, d**) Graph, quantification from three different experiments. ANOVA (one way analysis of variance); *, $p < 0.05$. (**e**) qRT-PCR analysis of the effects of flubendazole treatment on the expression TFEB target genes in HeLa cells. (**f**) qRT-PCR analysis of the effect of depletion of TFEB and MITF on flubendazole induced expression of TFEB target genes in HeLa cells. Statistics, student t test (e) and ANOVA (f). SD, *, $p < 0.05$; n=3. (**g**) Confocal images of immunofluorescence (LAMP1 antibody) in HeLa cells knocked down for TFEB alone or in combination with other TFE/MiTF members. Graph, mean LAMP1 intensity per cell ± SD (15-20 cells per condition analyzed using ImageJ, n=3); statistics, ANOVA (one way analysis of variance) ; *, $p<0.05$; † , $p \geq 0.05$. Scale bars, 20 µm.

Supplementary Figure 5

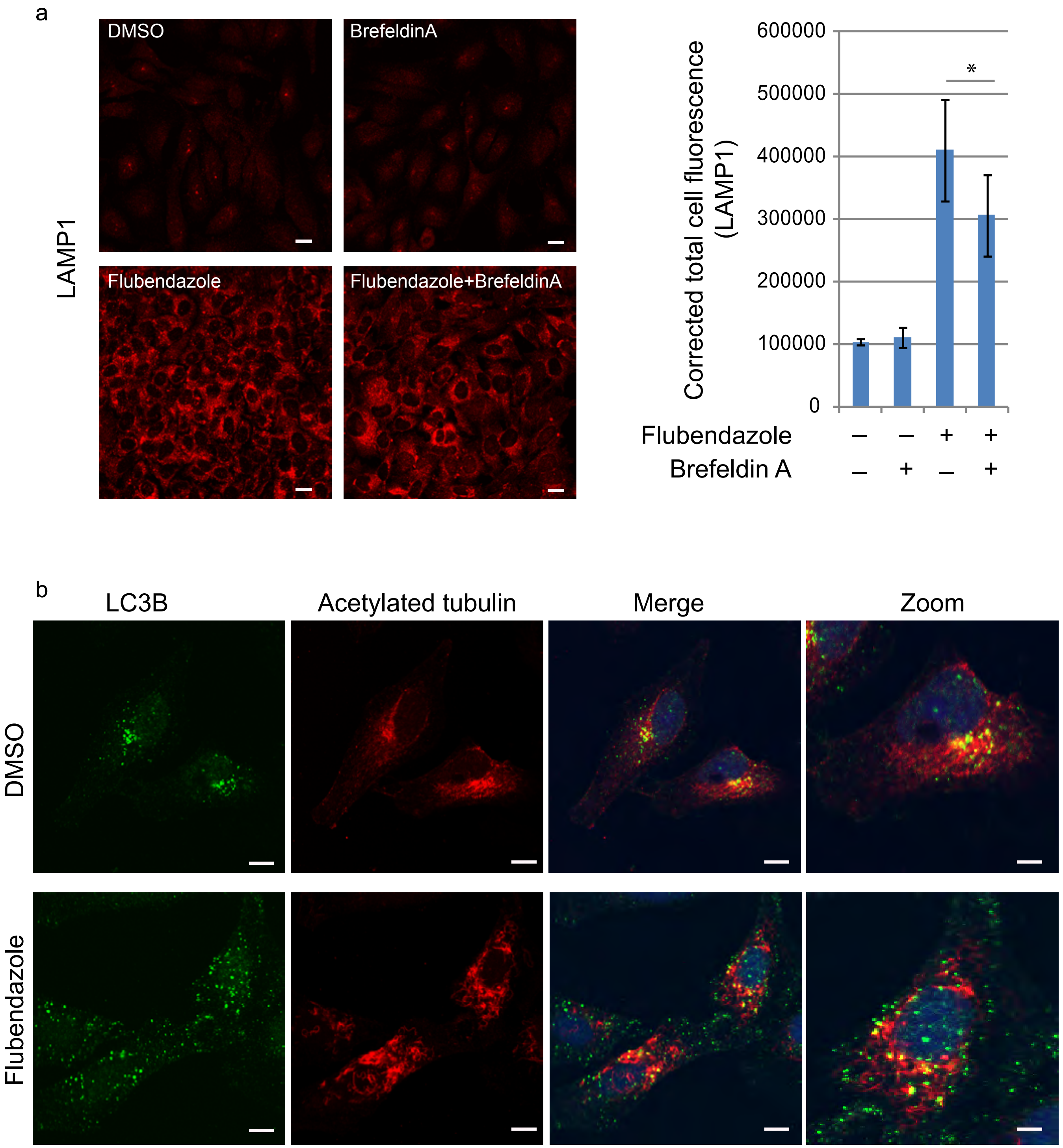

Supplementary Fig. 5. (a) Brefeldin A1 attenuates the effect of flubendazole on lysosome numbers as detected by anti-LAMP1 staining (red). Graph, mean corrected total cell fluorescence ± SD (25–35 cells measured using ImageJ); *$p < 0.05$ (t-test). Scale bar, 20 μm. (b) Autophagosomes co-localize with acetylated microtubules. Confocal images of HeLa cells treated with DMSO or flubendazole (1h, 5 μg/ml) and immuno-stained with antibodies to acetylated tubulin and LC3. Scale bars, 10 μm.

Supplementary Figure 6

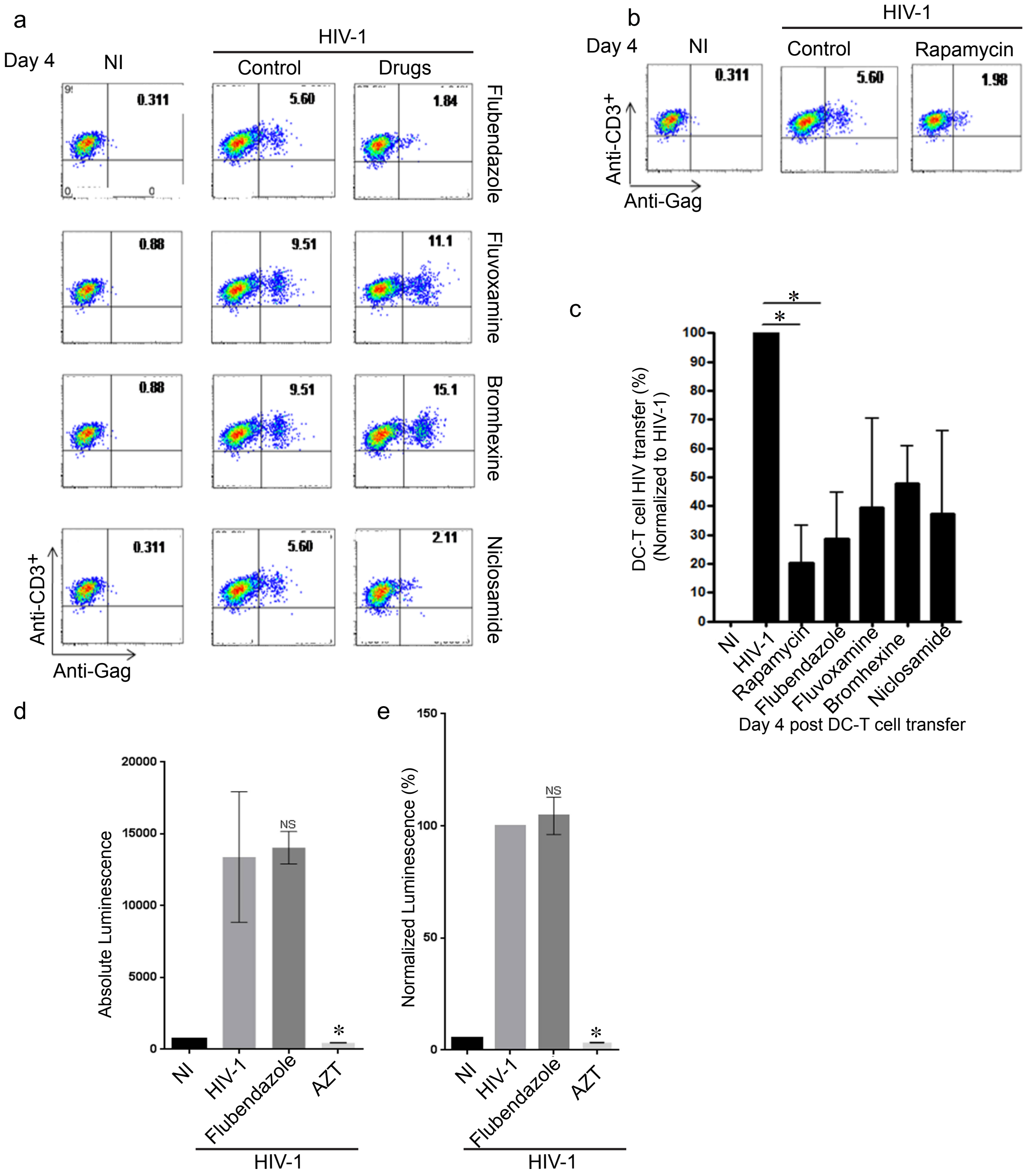

Supplementary Fig. 6. Effect of autophagy modulating drugs on transfer of HIV-1 from dendritic cells to CD4+T cells and on infection yield in a reporter cell line. (a, b) Flow cytometry analysis of DC-to-CD4+T cell mediated HIV-1 transfer, 4 days after treatment with drugs. (c) Quanitification analysis of flow cytometric analyses illustrated in panels a and b. (d,e) Absolute or normalized luminescence of Hela TZMBL reporter cell line treated with 5μM flubendazole or 0.1 mg/mL AZT for 2 h prior to infection with HIV-1 X4 for 48 h. Data, means ± SD (c) or SE (d,e) (n>3); *, $p<0.05$ (t test).

Supplementary Figure 7

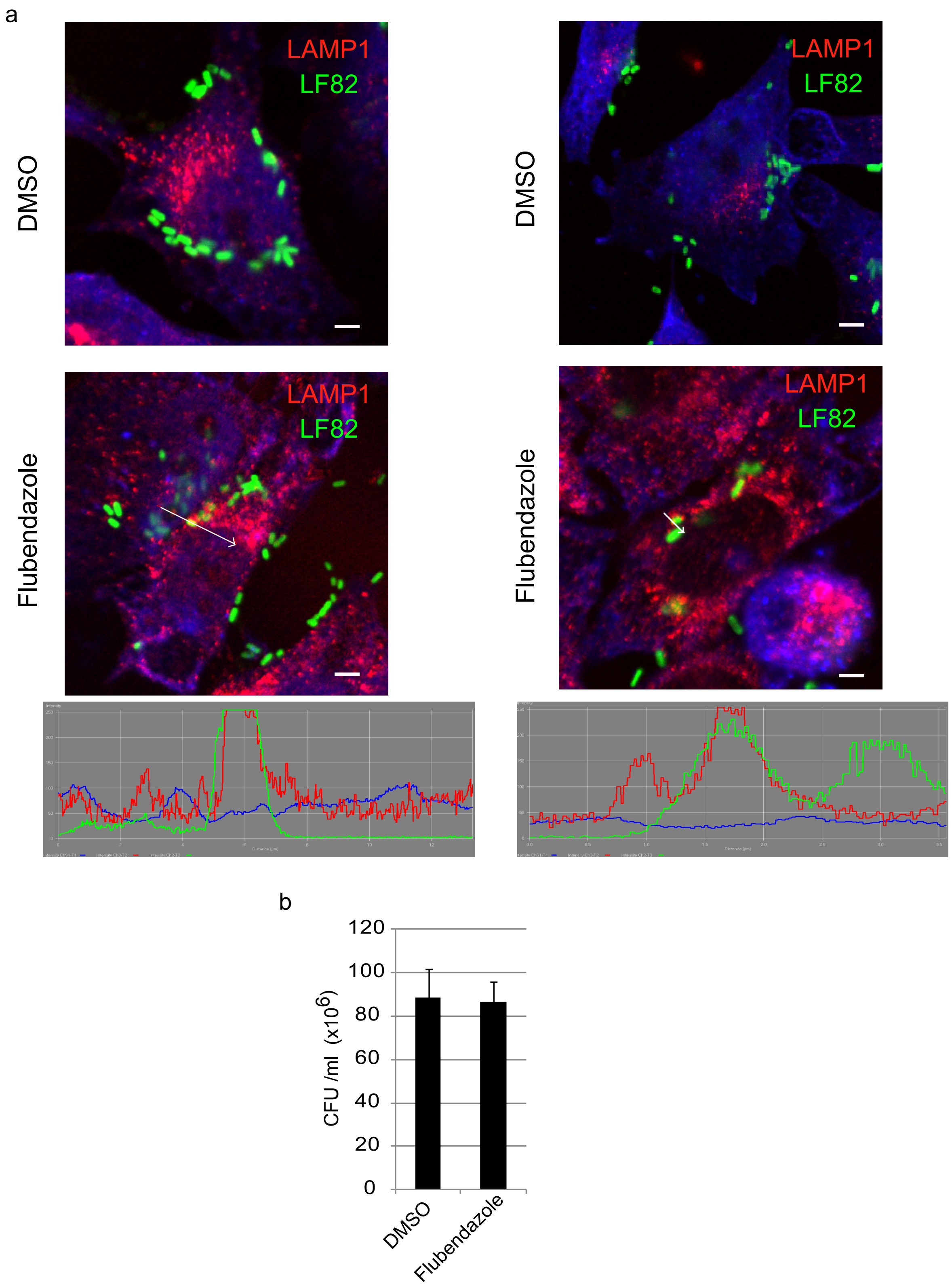

Supplementary Fig. 7. (a) Confocal image analysis of flubendazole effects on colocalization of *E.coli* LF82 and LAMP1. (b) Effect of flubendazole treatment (30 min, 5 µg/ml) on *E. coli* LF82 growth in liquid broth. Data, means ± SD (n=3). Scale bar, 10 µm.

Supplementary Figure 8

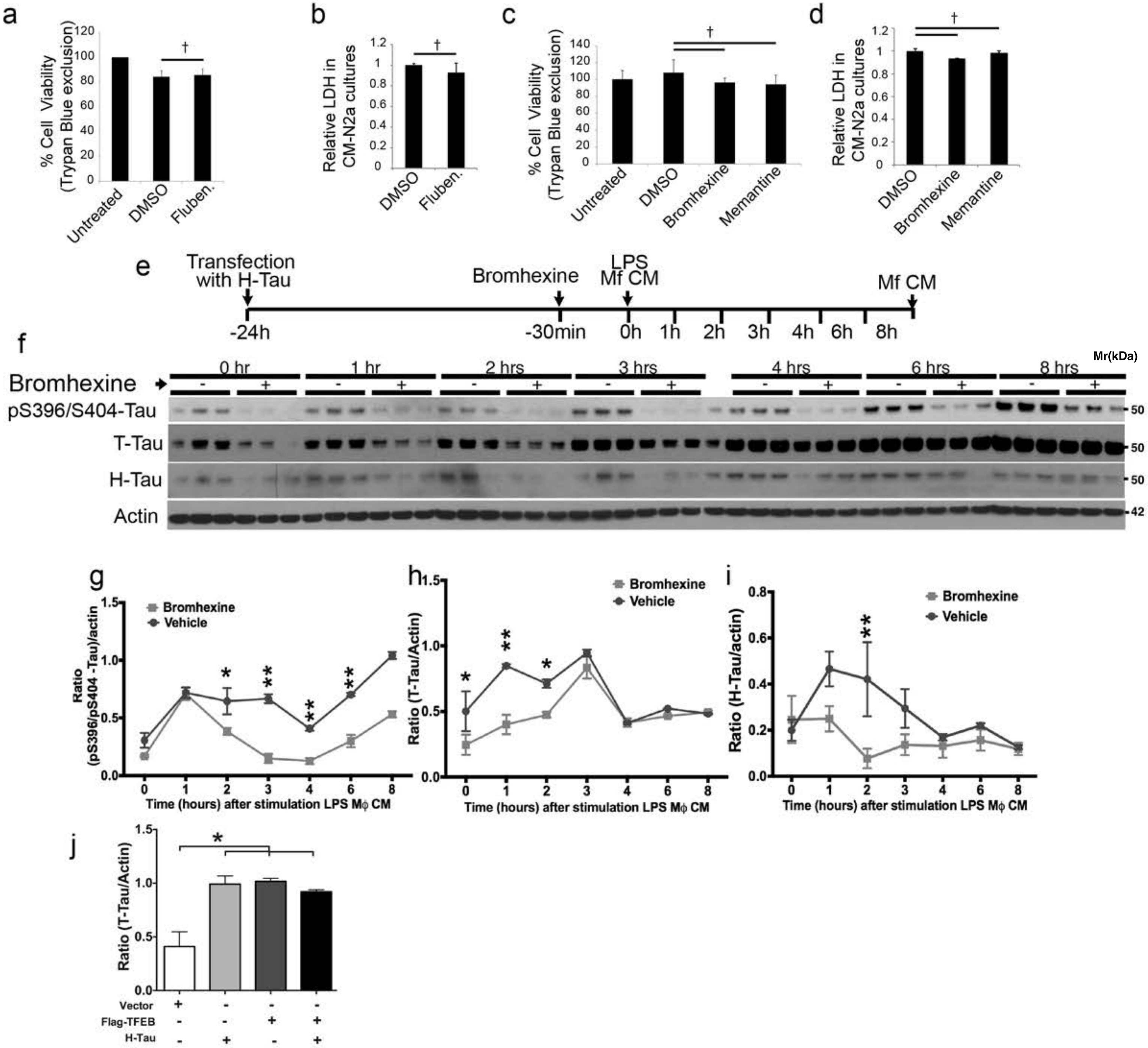

Supplementary Fig. 8. Dynamics of bromhexine-mediated clearance of tau as a function of time and assessment of cell viability. Cell death in cells treated with DMSO (vehicle) or drugs (as indicated) was determined by trypan blue exclusion (**a**, **c**) and LDH release assays (**b,d**) using identical conditions employed to study the tau clearance. (**e**) Schematic showing the experimental design. N2a cells were transfected with human tau (H-tau) carrying T231D/S235D disease-associated phosphorylation-mimicking mutation. After 24 h, N2a cells were pre-treated with bromhexine for 30 min followed by treatment with LPS-treated macrophage (Mf or MΦ) conditioned media (CM) for different time-points. N2a cells were lysed and processed for Western blot analysis. (**f**) Western blot showing reduction in the tau phosphorylated at S396/S404 (reactive to PHF1 antibody), human and endogenous mouse tau both recognized by Tau5 antibody (total tau; T-Tau), or only human tau (H-Tau, recognized by Tau12, a human tau-specific antibody). Actin, loading control. (**g-i**) Quantification. Data, means ± SEM (*$p<0.05$; **$p<0.01$; n=3; two-way ANOVA with Sidak's multiple comparison test). (**j**) Quantification of T-Tau/Actin ratios. Data, means ±SEM; $p<0.05$; n=3, one-way ANOVA with Tukey's multiple comparison test).

Supplementary Figure 9

Scanned Full Blots

Fig. 1b

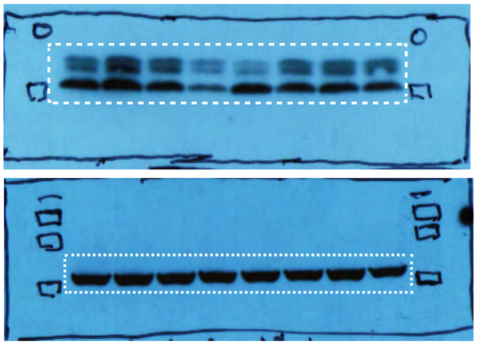

Fig. 1d

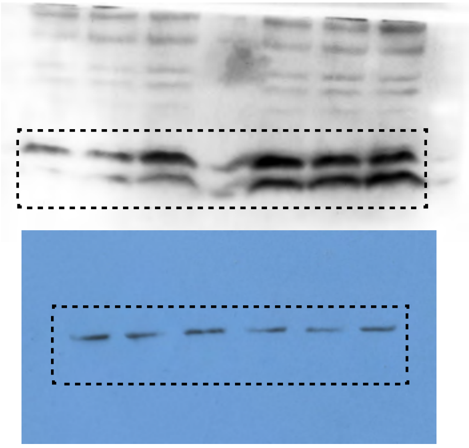

Fig. 1g

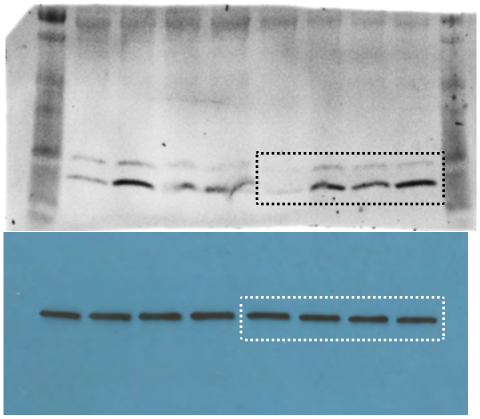

Fig. 1k

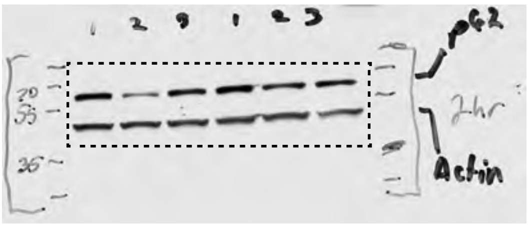

Fig. 2b

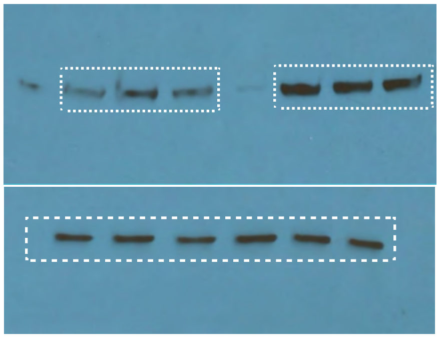

Fig. 2c

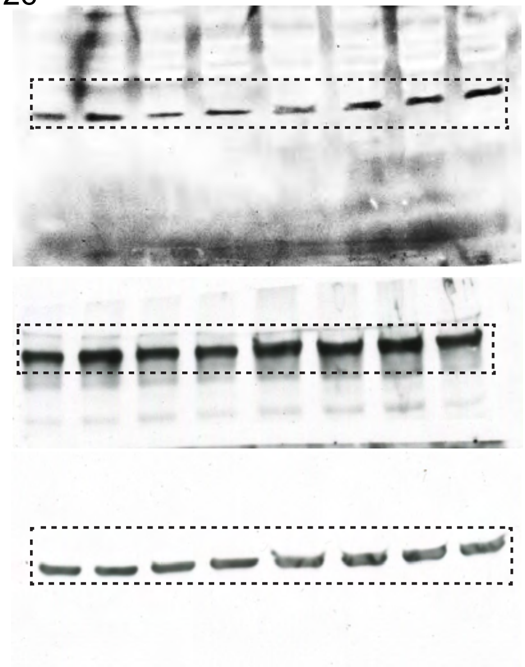

Supplementary Figure 10

Scanned Full Blots

Fig. 2e

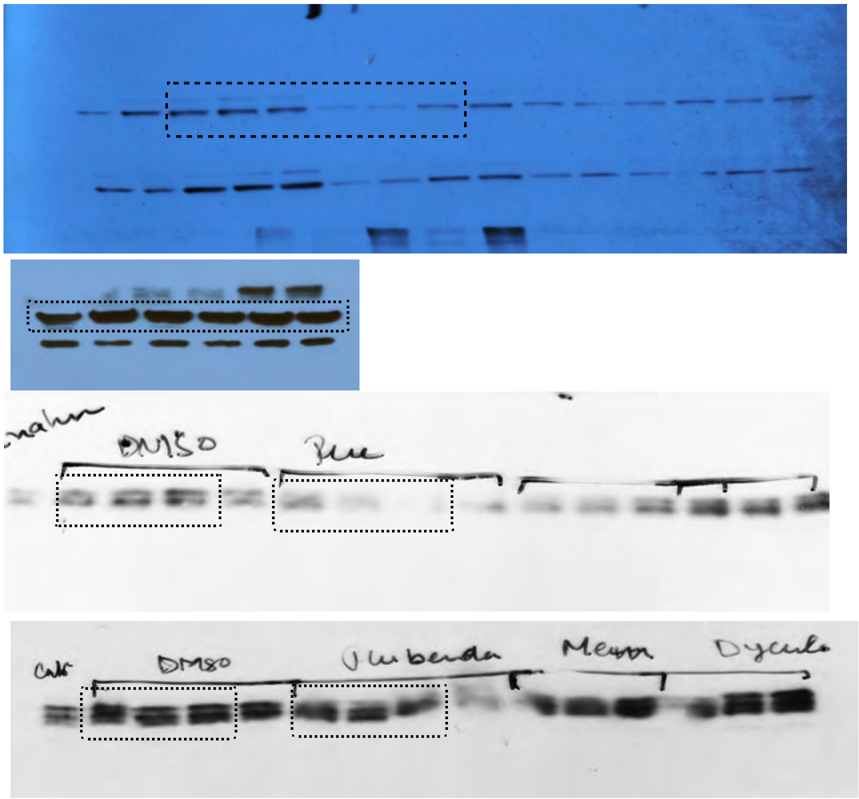

Fig. 3e

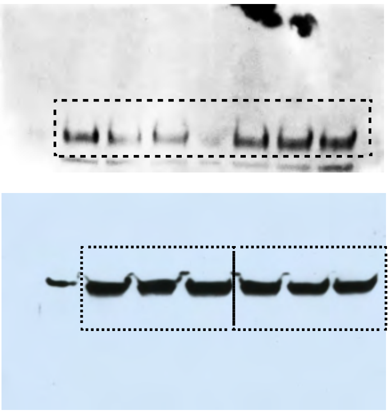

Fig. 4a

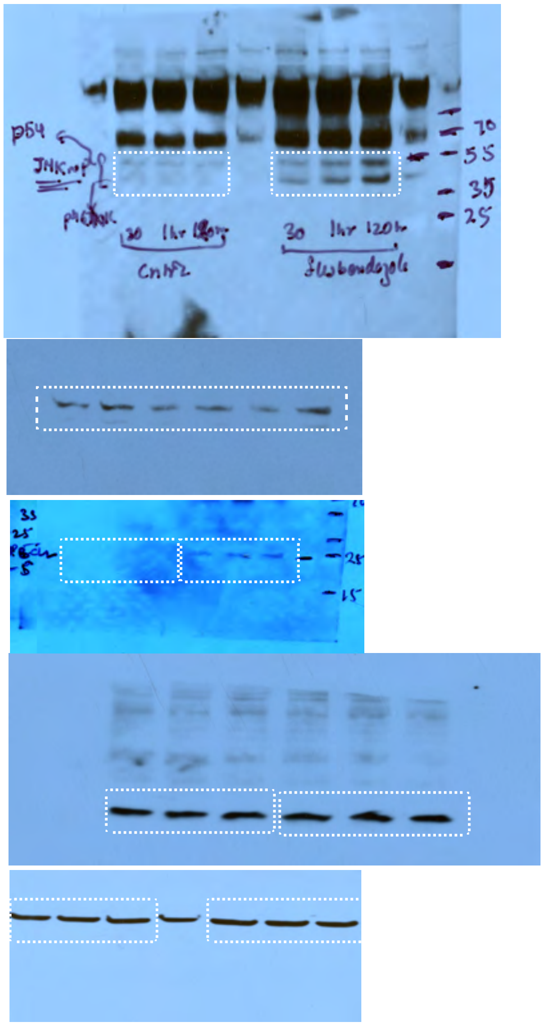

Fig. 4b

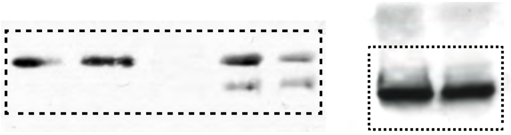

Supplementary Figure 11

Scanned Full Blots

Fig. 4c

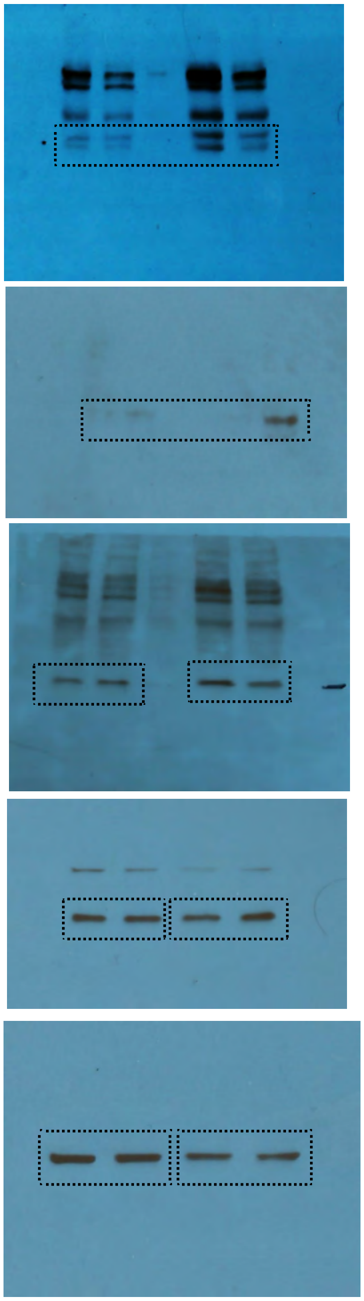

Fig. 4d

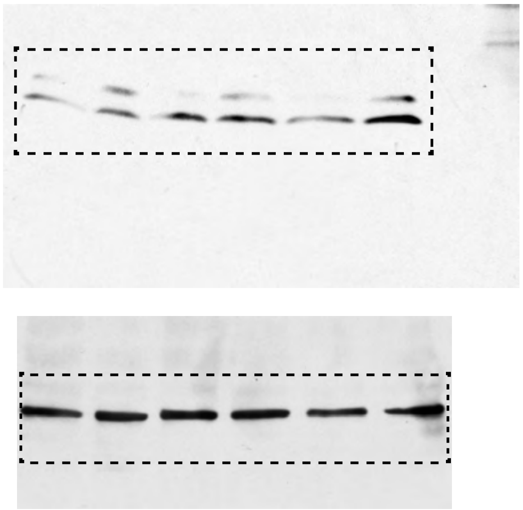

Supplementary Figure 12

Scanned Full Blots

Fig. 7b

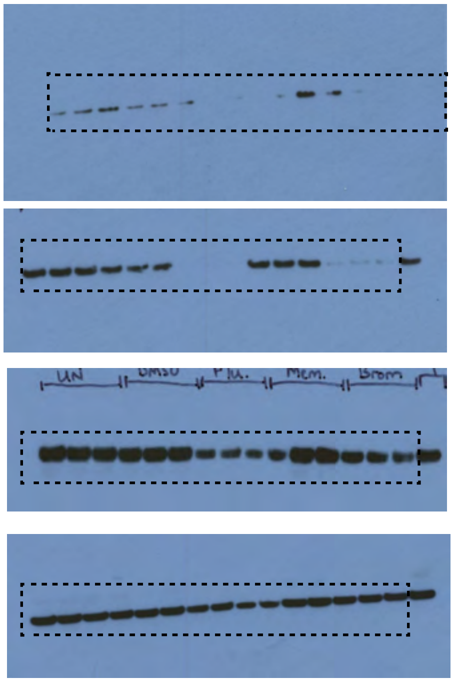

Fig. 7d

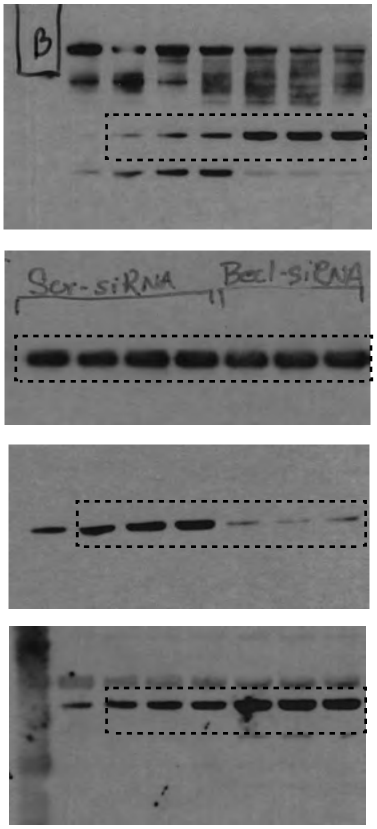

Fig. 7e

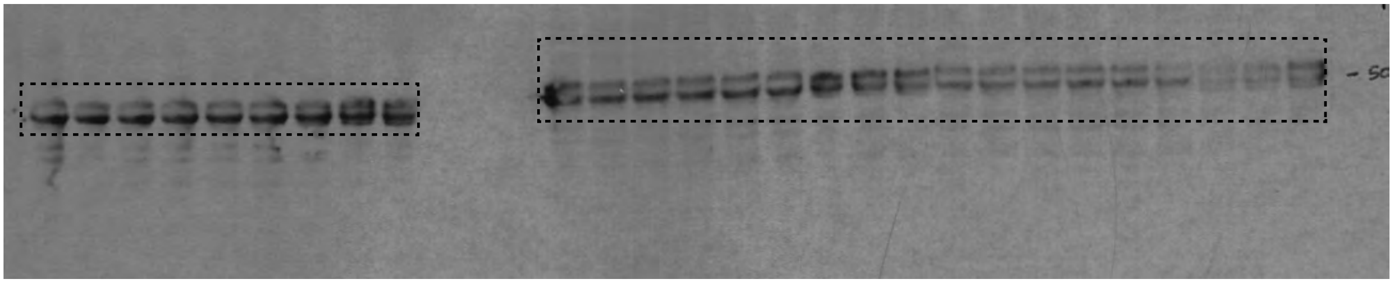

Supplementary Figure 13

Scanned Full Blots

Fig. 7f

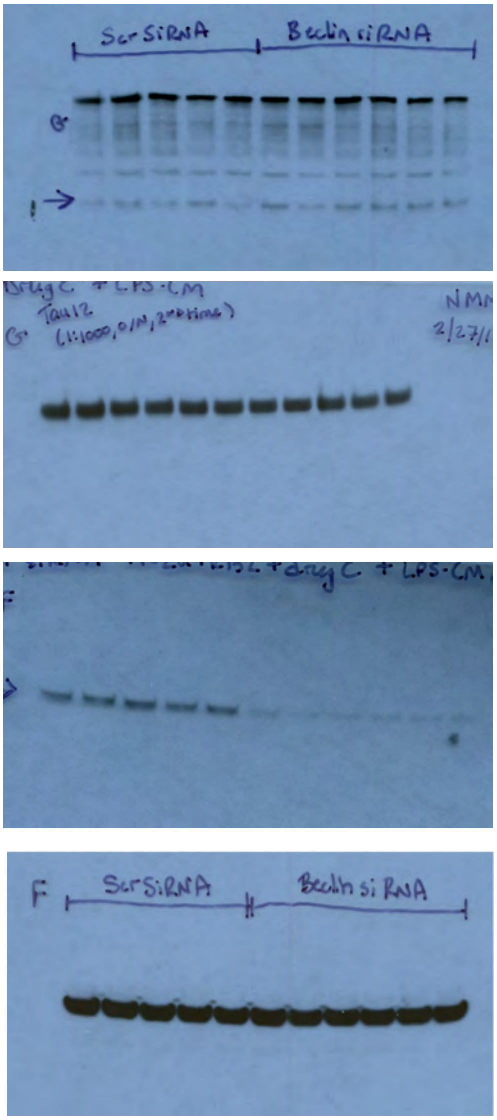

Fig. 7g

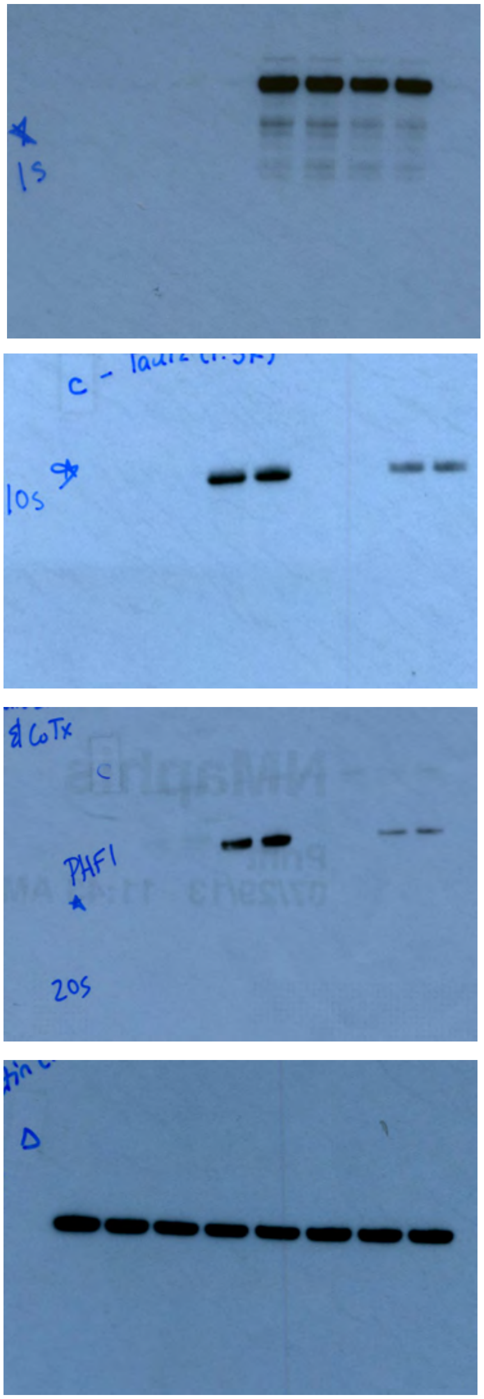

Supplementary Table 1. Initial screen summary: autophagy induction by GFP-LC3 puncta parameters (positive hits)

Compound[†]	Published Autophagy regulator	Autophagy Induction[a] GFP-LC3 puncta area fold increase average	GFP-LC3 puncta number fold increase average
1. Astemizole	(1)	6.66	3.78
2. Reserpine	(2)	6.6	4.35
3. Chrysophanol	No	6.6	3.14
4. Emetine	(3)	6.58	3.05
5. Chlorosalicylanilide	No	6.21	4.08
6. Oxiconazole	No	5.25	2.43
7. Sibutramine	No	5.24	4
8. Proadifen	No	5.20	2.86
9. Dihydroergotamine tartrate	No	5.05	3.35
10. Terfenadine	(4)	4.99	3.34
11. Triflupromazine	No	4.84	2.83
12. Amiodarone	(5)	4.49	3.25
13. Saponin	(6)	4.05	3.02
14. Vinblastine	(7)	3.95	2.11
15. Flubendazole	No	3.7	2.57

**16. Niclosamide**	(8)	3.65	1.96
**17. Hexachlorophene**	No	3.5	3.31
**18. Monensin**	(9)	3.3	2.13
**19. Phenylmercuric acetate**	No	3.3	0.19
**20. Tannic acid**	No	3.2	1.92
**21. Toremiphene citrate**	No	3.1	1.60
**22. Fenticlor**	No	2.96	1.54
**23. Pizotyline Malate**	No	2.8	2.31
**24. Piperacetazine**	No	2.77	2.09
**25. Bepridil**	(9)	2.7	2.05
**26. Oxyphencyclimine**	No	2.6	1.46
**27. Clomiphene citrate (Z,E)**	No	2.55	1.93
**28. Propafenone**	(9)	2.5	1.80
**29. Loperamide**	(9)	2.5	1.77
**30. GBR 12909**	No	2.5	1.74
**31. Glyburide**	No	2.45	0.90
**32. Sertraline**	(2)	2.4	1.75
**33. Nilvadipine**	No	2.4	1.81
**34. Hydroxychloroquine**	(10)	2.4	2.14

**35. Methotrimeprazine**	No	2.38	2.11
**36. Desipramine**	(11)	2.3	1.95
**37. Mepartricin**	No	2.21	2.21
**38. Fluvoxamine**	No	2.2	1.62
**39. Thiamylal Sodium**	No	2.2	1.88
**40. Trilocarban**	No	2.18	2.16
**41. Dicyclomine**	No	2.1	1.60
**42. Diphenidol**	No	2.08	2.01
**43. Ticlopidine**	(3)	2.05	1.43
**44. Maprotiline**	(12)	2.05	1.75
**45. Memantine**	No	2.05	1.54
**46. Karanjin**	No	2.05	1.36
**47. Melengestrol**	No	2	1.61
**48. Clovanediol diacetate**	No	2	1.62
**49. Bromhexine**	No	1.95	1.56
**50. Nerolidol**	No	1.95	2.32
**51. Fluoxetine**	(12)	1.94	1.64
**52. Helenine**	No	1.9	2.65
**53. Dehydroabietamide**	No	1.9	2.68

**54. Dibutyl Phthalate**	No	1.89	1.93
**55. 18-aminoabieta-8,11,13-triene sulfate**	No	1.85	3.32
**56. Podophyllin acetate**	No	1.85	2.86
**57. Berbamine**	(9)	1.8	2.76
**58. Rotenone**	(13)	1.8	1.52
**59. Rubescensin A**	No	1.8	1.57
**60. Zotepine**	No	1.8	1.56
**61. Morin**	No	1.8	2.41
**62. Pyrromycin**	No	1.8	6.29
**63. Pomiferin**	No	1.8	2.73
**64. Gardenin A**	No	1.75	1.58
**65. Ethoxyquin**	No	1.65	1.72
**66. Nocodazole**	(14)	1.65	1.36
**67. alpha-mangostin**	(15)	1.65	3.58
**68. Avocadene**	No	1.65	1.42
**69. Butylated hydroxytoluene**	No	1.6	1.29
**70. Physcion**	No	1.6	1.56
**71. Diperodon**	No	1.55	1.31
**72. Tetrandrine**	(16)	1.55	2.10

**73. Malathion**	No	1.55	1.30
**74. Isoliquiritigenin**	(17)	1.5	1.95
**75. Clofoctol**	No	1.4	1.38
**76. Isoreserpine**	No	1.4	1.90
**77. 4,4'-dimethoxydalbergione**	No	1.4	1.58
**78. 4-methyldaphnetin**	No	1.3	1.27
**79. Nortriptyline**	(18)	1.12	1.09
**80. Tetrachloroisophthalonitrile**	No	1.05	0.39

†Black, Prestwick Chemical Library screen; Red, Spectrum 2000 screen; Green, Johns Hopkins library screen.

1. Hu WW, *et al.* (2012) H1-antihistamines induce vacuolation in astrocytes through macroautophagy. *Toxicology and applied pharmacology* 260(2):115-123.
2. Park SJ, *et al.* (2012) Mitochondrial fragmentation caused by phenanthroline promotes mitophagy. *FEBS letters* 586(24):4303-4310.
3. Hundeshagen P, Hamacher-Brady A, Eils R, & Brady NR (2011) Concurrent detection of autolysosome formation and lysosomal degradation by flow cytometry in a high-content screen for inducers of autophagy. *BMC biology* 9:38.
4. Nicolau-Galmes F, *et al.* (2011) Terfenadine induces apoptosis and autophagy in melanoma cells through ROS-dependent and -independent mechanisms. *Apoptosis : an international journal on programmed cell death* 16(12):1253-1267.
5. Sarkar S, *et al.* (2007) Small molecules enhance autophagy and reduce toxicity in Huntington's disease models. *Nature chemical biology* 3(6):331-338.
6. Ellington AA, Berhow M, & Singletary KW (2005) Induction of macroautophagy in human colon cancer cells by soybean B-group triterpenoid saponins. *Carcinogenesis* 26(1):159-167.
7. Shen S, *et al.* (2011) Association and dissociation of autophagy, apoptosis and necrosis by systematic chemical study. *Oncogene* 30(45):4544-4556.
8. Balgi AD, *et al.* (2009) Screen for chemical modulators of autophagy reveals novel therapeutic inhibitors of mTORC1 signaling. *PloS one* 4(9):e7124.

9. Zhang L*, et al.* (2007) Small molecule regulators of autophagy identified by an image-based high-throughput screen. *Proceedings of the National Academy of Sciences of the United States of America* 104(48):19023-19028.
10. Ramser B*, et al.* (2009) Hydroxychloroquine modulates metabolic activity and proliferation and induces autophagic cell death of human dermal fibroblasts. *The Journal of investigative dermatology* 129(10):2419-2426.
11. Ma J*, et al.* (2013) Antidepressant desipramine leads to C6 glioma cell autophagy: implication for the adjuvant therapy of cancer. *Anti-cancer agents in medicinal chemistry* 13(2):254-260.
12. Cloonan SM & Williams DC (2011) The antidepressants maprotiline and fluoxetine induce Type II autophagic cell death in drug-resistant Burkitt's lymphoma. *International journal of cancer. Journal international du cancer* 128(7):1712-1723.
13. Chen Y, McMillan-Ward E, Kong J, Israels SJ, & Gibson SB (2007) Mitochondrial electron-transport-chain inhibitors of complexes I and II induce autophagic cell death mediated by reactive oxygen species. *Journal of cell science* 120(Pt 23):4155-4166.
14. Shen S*, et al.* (2012) Cytoplasmic STAT3 represses autophagy by inhibiting PKR activity. *Molecular cell* 48(5):667-680.
15. Chen JJ*, et al.* (2013) Inhibition of autophagy augments the anticancer activity of alpha-mangostin in chronic myeloid leukemia cells. *Leukemia & lymphoma*.
16. Gong K*, et al.* (2012) Autophagy-related gene 7 (ATG7) and reactive oxygen species/extracellular signal-regulated kinase regulate tetrandrine-induced autophagy in human hepatocellular carcinoma. *The Journal of biological chemistry* 287(42):35576-35588.
17. Chen G*, et al.* (2012) Mammalian target of rapamycin regulates isoliquiritigenin-induced autophagic and apoptotic cell death in adenoid cystic carcinoma cells. *Apoptosis : an international journal on programmed cell death* 17(1):90-101.
18. Sundaramurthy V*, et al.* (2013) Integration of chemical and RNAi multiparametric profiles identifies triggers of intracellular mycobacterial killing. *Cell host & microbe* 13(2):129-142.

Supplementary Table 2. LC3-II conversion assay

Compound	Statistical significance	Effect
Bromhexine	p<0.05	Inducer
Clomiphene citrate	NS	
Desipramine	p<0.05	Inducer
Dicyclomene	NS	
Dihydroergotamine Tartate	NS	
Diperodon	p<0.05	Inducer
Diphenidol	p<0.05	Inducer
Emetine	p<0.05	Inhibitor
Ethoxyquin	p<0.05	Inhibitor
Flubendazole	p<0.05	Inducer
Fluoxetine	p<0.05	Inducer
Fluvoxamine	NS	
GBR12909	p<0.05	Inhibitor
Hexaclorophene	p<0.05	Inducer
Maprotiline	NS	
Melangestol	p<0.05	Inducer
Memantine	NS	
Mepartricin	NS	
Methotrimeprazinet	NS	
Nivaldipine	NS	
Nortriptyline	NS	
Oxiconazole	NS	
Piperacetazine	NS	
pp242 (control)	p<0.05	Inducer
Poadifen	NS	
Sertraline	NS	
Ticlopidine	NS	
Triflupromazine	NS	
Zotopine	NS	

Supplementary Table 3

MT-targeting agents	MT-depolymerization	MT-acetylation/stablization	Autophagosome number	Lysosome number	Autophagic flux	Reference
**Nocodozole**	Yes	No	No (inhibit)	No	inhibit	[1 2]
**Taxol**	No	Yes	No	No	inhibit	[3 2]
**Vinblastine**	Yes (precipitation)	No	Yes	No	inhibit	[1 2]
**Vincristine**	Yes	No	No	No (size-yes)	inhibit	[4]
**Flubendazole**	Yes	Yes	Yes	Yes	Induce	This study

1. Kochl, R., Hu, X.W., Chan, E.Y. & Tooze, S.A. Microtubules facilitate autophagosome formation and fusion of autophagosomes with endosomes. *Traffic* **7**, 129-145 (2006).
2. Xie, R., Nguyen, S., McKeehan, W.L. & Liu, L. Acetylated microtubules are required for fusion of autophagosomes with lysosomes. *BMC cell biology* **11**, 89 (2010).
3. Veldhoen, R.A.*, et al.* The chemotherapeutic agent paclitaxel inhibits autophagy through two distinct mechanisms that regulate apoptosis. *Oncogene* **32**, 736-746 (2013).
4. Groth-Pedersen, L., Ostenfeld, M.S., Hoyer-Hansen, M., Nylandsted, J. & Jaattela, M. Vincristine induces dramatic lysosomal changes and sensitizes cancer cells to lysosome-destabilizing siramesine. *Cancer research* **67**, 2217-2225 (2007).
